# Supplementary material for: A post-translational modification of human Norovirus capsid protein attenuates glycan binding
Source: Nat Commun. 2019 Mar 21;10:1320. doi: 10.1038/s41467-019-09251-5 (PMC6428809; doi:10.1038/s41467-019-09251-5)
Supplement: Supplementary file 1 — Supplementary Information [file 41467_2019_9251_MOESM1_ESM.pdf]

SUPPLEMENTARY INFORMATION

A post-translational modification of human Norovirus capsid protein attenuates glycan binding

Mallagaray *et al.*

This PDF file includes:

Supplementary notes 1 - 6

Supplementary figures 1 - 26

Supplementary tables 1 -13

Supplementary references

Table of Contents

1. **Supplementary note 1:** D/H exchange protocol for Norovirus GII.4 Saga 2006 P domain 4

1.1 RP-HPLC 4

1.2 Electrophoresis 5

1.3 NMR experiments to evaluate efficiency of D/H exchange and protein integrity 5

1.4 Determination of unfolding rate, GuHCl concentration and unfolding time required for complete D/H backbone exchange 5

1.5 Preparation of master stock solutions and protein renaturing solutions for screening of refolding conditions 8

1.6 Screening refolding conditions 10

1.7 Evaluation of Saga P-dimers selectivity towards HBGAs after unfolding/refolding protocol and quantification of D/H exchange 12

1.8 Final D/H exchange protocol: 14

2. **Supplementary note 2:** NMR experiments 16

2.1 Optimization of sample conditions for 3D NMR experiments 16

2.1.1 ^{1}H and $^{1}H,^{15}N$ TROSY HSQC 16

2.1.2 1D [^{15}N, ^{1}H]-TRACT experiments 17

2.1.3 Spin-echo NMR experiments 18

2.1.4 Dynamic light scattering (DLS) 19

2.2 3D NMR spectroscopy 21

2.2.1 Experimental parameters 21

2.2.2 Chemical shift-based secondary structure prediction 21

2.2.3 Backbone amide chemical shifts of deamidated Saga P-dimers 22

2.3 Chemical shift titrations 23

2.3.1 NMR experimental conditions 23

2.3.2 Quantification of chemical-shift perturbations 23

2.3.3 Minimum threshold for significant chemical shift perturbations 23

2.3.4 Sample preparation and HBGA titrations 25

2.3.5 Titrations with methyl α-L-fucopyranoside 25

2.3.6 Chemical shift perturbations in the presence of blood group B trisaccharide 26

2.3.7 Noise reduction of chemical shift titration data 26

2.3.8 Covariance analysis and hierarchical clustering of binding isotherms for the titration of NN P-dimers with methyl α-L-fucopyranoside 28

2.3.9 Non-linear least squares global fitting yields dissociation constants K_D 29

2.3.10 Reevaluation of previously published titration data of methyl α-L-fucopyranoside binding to [U-^{2}H,^{15}N] Saga P-dimers 32

3 **Supplementary note 3:** Sequence and structural alignment of P-domains of GII.4 norovirus strains ... 34
3.1 Screening the Protein Data Bank for N373 in the reactive conformation ... 35
4 **Supplementary note 4:** Deamidation of P-dimers ... 39
4.1 Rate of deamidation of GII.4 Saga P-dimers ... 39
4.2 Deamidation of P-dimers of strains GII.4 MI001, GII.10 Vietnam 026, and GII.17 Kawasaki 308 40
5 **Supplementary note 5:** Crystallography ... 42
5.1 Ligand interaction diagram ... 42
5.2 Data collection and refinement statistics ... 43
6 **Supplementary note 6:** Mass spectrometry ... 44
6.1 Identification of deamidation sites ... 44
6.2 P-domain peptide coverage ... 47
6.3 HDX data summary tables ... 48
Supplementary references: ... 50

1. Supplementary note 1: D/H exchange protocol for Norovirus GII.4 Saga 2006 P domain

Initially, only ca. 44% of all backbone NH resonance signals could be assigned, precluding a comprehensive CSP analysis. Based on the Saga P-dimer crystal structure[1] it was evident that most of the assigned amino acids were located in water-exposed flexible loops whereas only very few were located in β-sheets, α-helixes, or at the dimer interface. Further inspection of ^{1}H,^{15}N TROSY HSQC spectra of P-dimers revealed that ca. 30% of all expected NH backbone signals were missing due to very slow exchange of backbone deuterons D^N to H^N. Deuterons originate from protein synthesis in fully deuterated minimal media, with D_2O as solvent. Therefore, completing the backbone assignment required exchange of backbone deuterons D^N with backbone protons H^N. ^{1}H,^{15}N TROSY HSQC spectra of samples incubated at 298 K for up to 72 h did not show any improvement of D^N/H^N exchange, and higher temperatures caused protein precipitation. Therefore, we resorted to the use of chaotropic reagents for unfolding. Unlabeled P-dimers were subjected to unfolding-refolding experiments as explained in the following.

For the development of the D/H exchange protocol we conducted a refolding screen based on a previously reported strategy [2]. The refolding efficiency and the quality of the refolded protein were analyzed primarily by RP-HPLC and SDS-PAGE, and in a second instance by NMR.

1.1 RP-HPLC

A Merck Hitachi D-7000 HPLC system equipped with a Merck-Hitachi L-7400 UV detector was used to quantify properly and improperly folded protein. A reverse-phase (RP) Eurospher 100 C18 (5 µm, 25 mm x 4 mm) column was equilibrated in 25% acetonitrile (ACN), 0.1 % trifluoroacetic acid (TFA) and 2 mM β-mercaptoethanol in H_2O at a flow rate of 1 ml/min. Samples were filtered prior injection, 20 µg of protein were injected, and protein was eluted with a linear gradient from 25% to 60% ACN in 0.1 % trifluoroacetic acid (TFA) and 2 mM β-mercaptoethanol for 40 min at a flow rate of 1 ml/min. The elution profile was monitored at 214 nm. RP-HPLC buffers were freshly prepared and degassed each day.

Properly and improperly folded protein eluted as two peaks. Respective retention times were determined using freshly expressed Saga P-dimers and Saga P-dimers incubated overnight in 4 M GuHCl / 10 mM β-mercaptoethanol. To calculate protein concentrations, a calibration curve was determined for freshly expressed Saga P-dimers using 10, 20, 30, 40, and 50 µg of protein. Gaussian or Pearson IV functions were fitted to the peaks using Origin2016G, and resulting areas were plotted against protein concentrations. A linear correlation between fitted areas and protein concentration was observed for the concentration range analyzed, resulting in an accuracy of ± 3.5% for a 95% confidence interval. Using this reference amounts of properly and improperly folded protein after protein refolding were calculated from RP-HPLC runs. Not eluted protein is defined as protein which is not recovered from RP-HPLC, and was calculated using Supplementary Equation (1):

$$\text{Not eluted protein} = \text{Injected protein} - \text{properly folded protein} - \text{improperly folded protein} \quad (1)$$

Not eluted traces of protein were removed after every run by cleaning the system with a solution containing 6 M GuHCl and 100 mM β-mercaptoethanol.

1.2 Electrophoresis

SDS-PAGE was used to monitor protein decomposition during the unfolding step and to assess the purity of the refolded protein. Saga P-dimers were analyzed on a 15% polyacrylamide gel under reducing conditions and stained with GelCode® blue stain agent (Thermo Scientific). The broad range marker #161-0317 from BioRad was used as reference.

1.3 NMR experiments to evaluate efficiency of D/H exchange and protein integrity

Samples containing refolded [*U*-^{2}H,^{15}N] Saga P-dimers were prepared at concentrations of 50-80 µM in 20 mM Na-phosphate buffer (pH 7.20), containing 100 µM DSS-*d6* and 8% D_2O (160 µl). For each sample a ^{1}H,^{15}N TROSY HSQC spectrum was acquired as follows. A standard Bruker pulse program (trosyetf3gpsi.2) yielding phase sensitive spectra was used applying 32 dummy scans and 48 scans at a spectral resolution of 7.8 Hz and 15.8 Hz for the direct and indirect dimensions, respectively (experiment time: 7 h and 7 min). The frequency offset and spectral width were 4.69 ppm and 16.0 ppm, respectively, for the ^{1}H dimension and 118.5 ppm and 40.0 ppm, respectively, for the ^{15}N dimension. Spectra were processed with TopSpin 3.5pl6. Spectra were acquired on a Bruker AV III 500 MHz NMR spectrometer equipped with a TCI cryogenic probe.

1.4 Determination of unfolding rate, GuHCl concentration and unfolding time required for complete D/H backbone exchange

To determine the unfolding curve of Saga P-dimers eleven samples containing 1 ml at 0.2 mg per ml Saga P-dimers, 1 mM TCEP, 20 mM sodium phosphate buffer (pH 7.30) and guanidine hydrochloride (GuHCl) (0, 1, 1.25, 1.5, 1.75, 2, 2.5, 3, 4, 5 and 6 M) were incubated overnight at 4 °C. Before measurement, samples were centrifuged for 5 min at 4°C at 15,000 g. An unfolding curve for Saga P-dimers was obtained using a Jasco FP-6200 spectrofluorometer employing an excitation wavelength of 280 nm and plotting tryptophane maximum emission against the GuHCl concentration.

The GuHCl concentration and incubation time required for complete backbone D/H exchange combined with the highest protein recovery were determined as follows (see Supplementary Figure 1):

First, Saga P-dimers (2 mg per ml) were incubated in 20 mM sodium phosphate buffer (pH 7.3), 10 mM TCEP and 0.02% NaN_3 at GuHCl concentrations of 0, 1.47, 2, 3, 4 and 6 M at 4 °C. Aliquots of 3 µl were taken every 24 h over a total of 6 d and frozen at -80°C. In addition, the percentage of folded protein in the unfolding solution was determined by RP-HPLC from 10 µl aliquots. After 6 d samples were refolded by dilution (1:20) into refolding buffer consisting of 0.5 M Tris (pH 7.20), 0.3 M NaCl, and 10 mM TCEP. The percentage of properly folded protein was determined by RP-HPLC. Based on these results, 4 M GuHCl was selected for protein unfolding.

Second, the minimum incubation time in GuHCl required for a complete backbone D/H exchange was determined. Three samples each containing 0.5 ml of [*U*-^{2}H,^{15}N] Saga P-dimers

at a protein concentration of 2 mg per ml were denatured in buffer containing 20 mM sodium phosphate (pH 7.30), 10 mM TCEP, and 0.02% NaN_3 using 4 M GuHCl at 4 °C for 2h, 4h, and 24h, respectively. Samples were refolded by dilution (1:20) into 0.5 M Tris buffer (pH 7.20), 0.3 M NaCl and 10 mM TCEP. Refolded protein was purified by size exclusion chromatography to remove aggregates. D/H backbone exchange was evaluated by NMR as described in 2.3.

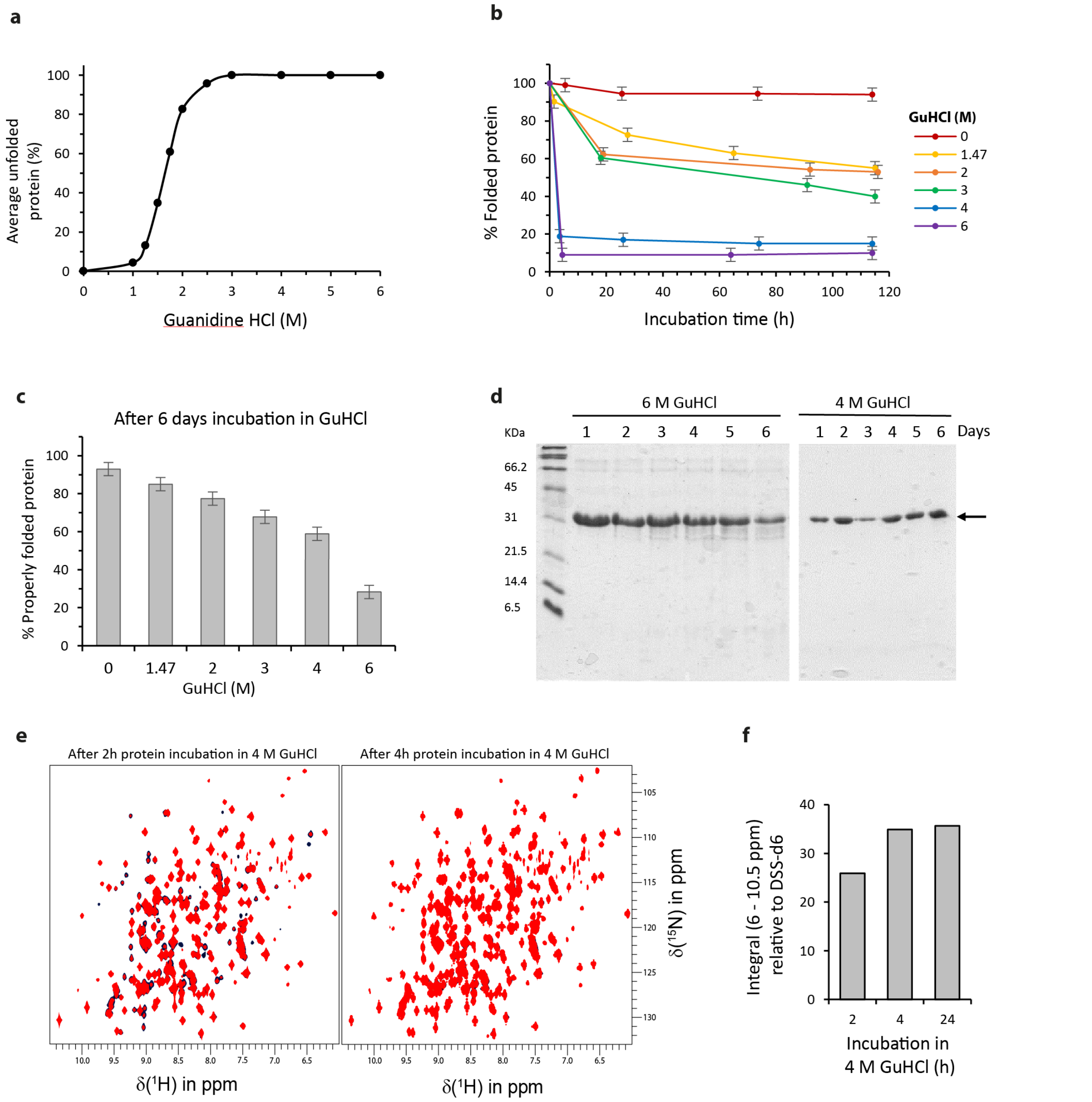

Supplementary Figure 1: Development of the D/H exchange protocol for *GII.4* Saga P-dimers. **a)** Unfolding curve for *GII.4* Saga P-dimers as a function of increasing GuHCl concentrations. At least 4 M GuHCl are required for complete protein unfolding (points are interpolated). **b)** Progression of unfolding with time at different GuHCl concentrations as obtained by RP-HPLC. As observed for the unfolding curve, only concentrations higher than 4 M GuHCl lead to complete protein unfolding. Note that for 4 M and 6 M GuHCl the protein was unfolded within the first 5 h. **c)** Percentage of properly folded protein obtained for samples from (b) after 6 d of incubation and refolding. Protein was refolded by dilution (1:20) in buffer containing 0.5 M Tris (pH 7.20), 0.3 M NaCl, and 10 mM TCEP. A 50% decrease in properly refolded protein is observed for 6 M GuHCl as compared to 4 M GuHCl. Errors in (b) and (c) were calculated from the RP-HPLC calibration curve for a 95% confidence interval. **d)** SDS-PAGE gels obtained for Saga P-dimers unfolded in 4 M and 6 M GuHCl as a function of time. The arrow indicates the band corresponding to Saga P monomers. Bands corresponding to protein decomposition were observed only for 6 M GuHCl, suggesting a higher protein stability under lower GuHCl concentrations. **e)** Section of ^{1}H,^{15}N TROSY HSQC spectra of [*U*-^{2}H,^{15}N] Saga P-dimers unfolded in 4 M GuHCl and refolded after 2h, 4h and 24h. *Left panel:* [*U*-^{2}H,^{15}N] Saga P-dimers refolded after 2h (red) and 24h (black). Right panel: [*U*-^{2}H,^{15}N] Saga P-dimers refolded after 4h (red) and 24h (black). **f)** Integrals relative to DSS-*d6* measured from 6 ppm to 10.5 ppm from 1D ^{1}H spectra for refolded [*U*-^{2}H,^{15}N] Saga P-dimers as a function of incubation time in 4 M GuHCl. No increase in signals for the backbone NH region was observed after 4h incubation, indicating a complete D/H exchange. NMR samples contained 70 µM of [*U*-^{2}H,^{15}N] *GII.4* Saga P-dimers and were prepared in buffer containing 75 mM sodium phosphate (pH 7.30), 100 mM NaCl and 100 µM DSS-*d6* in 10% D_2O. Spectra were acquired at 298 K.

1.5 *Preparation of master stock solutions and protein renaturing solutions for screening of refolding conditions*

Given the large amounts of protein required for protein NMR experiments and considering the cost of labeling proteins with stable isotopes, we decided to run a protein refolding screening in order to improve protein refolding yields. A set of master stock solutions was prepared as summarized in Supplementary Table 1 and stored at 4°C. Protein renaturing solutions were prepared from stock solutions as indicated in Supplementary Table 2 immediately before use and stored no longer than 30 d at 4°C. Note that since Saga P-dimers possess no disulfide bonds no redox system was added in this protocol.

Number	Name of stock sol.	Content	Concentration (M)
S.1	Acetate	Sodium acetate/acetic acid pH 4.5	0.91
S.2	Citrate	Sodium citrate/citric acid pH 5.30	0.91
S.3	MES	2-(N-morpholino)ethanesulfonic acid/HCl pH 6.13	0.91
S.4	Tris	Tris(hidroximetil)aminometano/HCl pH 7.20	0.91
S.5	TCEP	Tris(2-carboxyethyl)phosphine	0.1
S.6	NaSCN	Sodium thiocyanate	2
S.7	NaCl	Sodium chloride	1
S.8	NDSB 201	3-(1-Pyridinio)-1-ppropanesulfonate	2.3
S.9	Zwitt 3-12	Zwittergent 3-12	0.1% (w/v)
S.10	PEG 4000	Polyethylenglycol	0.25% (w/v)
S.11	D-Sucrose	D-Sucrose	1
S.12[A]	L-Arginine/MES	L-Arginine	0.91
S.13[A]	L-Histidine/MES	L-Histidine	0.91
S.14[A]	Betaine/MES	Betaine	0.91
S.15[A]	L-Lysine/MES	L-Lysine	0.91
S.16[A]	L-Proline/MES	L-Proline	0.91
S.17[B]	L-Proline/Tris	L-Proline	0.91
S.18[B]	Pro/Suc/Tris	L-Proline	0.91
		D-Sucrose	1

[A]Additives were directly dissolved in (S.3)-MES. [B]Dissolved in (S.4)-Tris.

Supplementary Table 1: Master stock solutions prepared for the refolding screen of Saga P-dimers. pH was calibrated after completely adding all additives. Solutions were prepared in Millipore(MP)-H_2O unless stated otherwise.

Influence of buffer and pH on protein refolding									
Number of renature solution	Name of renature solution	Mixing components (µl)				Final concentrations after refolding		
		Stock solution used	Vol. (µl)	S.5-TCEP (µL)	MP-H_2O (µl)	Additive	Conc. (M)	TCEP (mM)
R.1	Acetate pH 4.5	S.1-Acetate	274.7	10	190.3	Acetate	0.5	2
R.2	Citrate pH 5.3	S.2-Citrate	274.7	10	190.3	Citrate	0.5	2
R.3	MES pH 6.13	S.3-MES	274.7	10	190.3	MES	0.5	2
R.4	Tris pH 7.2	S.4-Tris	274.7	10	190.3	Tris	0.5	2

Influence of additives on protein refolding										
Number of renature solution	Name of renature solution	Mixing components (µl)					Final concentrations after refolding			
		Stock solution used	Vol. (µl)	S.5-TCEP (µl)	S.3-MES (µl)	MP-H_2O (µl)	Additive	Conc.	TCEP (mM)	MES (M)
R.5	NaSCN	S.6-NaSCN	75	10	274.7	115.3	NaSCN	0.3 M	2	0.5
R.6	NaCl	S.7-NaCl	150	10	274.7	40.3	NaCl	0.3 M	2	0.5
R.7	TCEP	-	-	50	274.7	150.3	TCEP	10 mM	10	0.5
R.8	NDSB 201	S.8-NDSB 201	100	10	274.7	90.3	NDSB 201	0.46 M	2	0.5
R.9	Zwitt 3-12	S.9-Zwitt 3-12	100	10	274.7	90.3	Zwitt 3-12	0.02% (w/v)	2	0.5
R.10	Glycerol	Glycerol 100%	150	10	274.7	40.3	Glycerol	30% (v/v)	2	0.5
R.11	PEG 4000	S.10-PEG 4000	100	10	274.7	90.3	PEG 4000	0.05% (w/v)	2	0.5
R.12	D-Sucrose	S.11-D-Sucrose	100	10	274.7	90.3	D-Sucrose	0.2 M	2	0.5
R.13	L-Arginine	S.12-L-Arginine/MES	274.7	10	-	190.3	L-Arginine	0.5 M	2	0.5
R.14	L-Histidine	S.13-L-Histidine/MES	274.7	10	-	190.3	L-Histidine	0.5 M	2	0.5
R.15	Betaine	S.14-Betaine/MES	274.7	10	-	190.3	Betaine	0.5 M	2	0.5
R.16	L-Lysine	S.15-L-Lysine/MES	274.7	10	-	190.3	L-Lysine	0.5 M	2	0.5
R.17	L-Proline	S.16-L-Proline/MES	274.7	10	-	190.3	L-Proline	0.5 M	2	0.5

Best combined conditions										
Number of renature solution	Name of renature solution	Mixing components (µl)					Final concentrations after refolding (M)			
		S.17-L-Proline/Tris	S.7-NaCl	S.11-D-Sucrose	S.18- Pro/Suc/Tris	MP-H_2O	L-Proline	NaCl	D-Sucrose	Tris
R.18[A]	L-Pro/NaCl	274.7	150	-	-	40.3	0.5	0.3	-	0.5
R.19[A]	L-Pro/D-Suc	-	-	-	274.7	190.3	0.5	-	0.2	0.5
R.20[A]	L-Pro/NaCl/D-Suc	-	150	-	274.7	40.3	0.5	0.3	0.2	0.5

[A]Samples contained 10 µl of stock solution (S.5)-TCEP, which translates into 2 mM TCEP after refolding.

Supplementary Table 2: Protein renature solutions prepared for protein refolding screening based on stock solutions from Supplementary Table 1. Solutions were stored no longer than 30 d at 4 °C.

1.6 Screening refolding conditions

Saga P-dimers were incubated for 4h at 4 mg per ml in 20 mM sodium phosphate buffer (pH 7.30) containing 4 M GuHCl and 2 mM TCEP at 4 °C for 4 h. For each refolding buffer, 25 µl of denatured protein was refolded by dilution (1:20). Refolding buffers were transferred in four pulses of 25, 50, 100 and 300 µl into the solution of denatured protein every 15 min during 1h. Each of the first 3 pulses corresponded to a two-fold dilution, which permitted slowing down the refolding process as compared to dropwise dilution, as explained in ref.[2]. At the end of the refolding process each sample contained 500 µl with a protein concentration of 0.2 mg per ml and the GuHCl concentration reduced to 0.3 M. The fractions of properly folded, improperly folded and not eluted protein were determined by RP-HPLC from a single 100 µl sample injection (Supplementary Table 3 and Supplementary Figure 2). MES was selected as buffer for the study of the influence of additives in order to elucidate their influence on protein folding. Buffers and flasks in contact with unfolded protein were precooled at 4 °C for at least 30 min.

Design of experiment number	Renature solution	Refolding yield assessed by RP-HPLC (%)		
		Properly folded protein	Improperly folded protein	Not eluted from RP-HPLC
**Influence of buffer and pH on protein refolding**				
1	R.1-Acetate pH 4.5	2	0	98
2	R.2-Citrate pH 5.3	5	0	95
3	R.3-MES pH 6.13	46	24	30
4	R.4-Tris pH 7.2	70	0	30
**Influence of additives on protein refolding**				
5[A]	R.3-MES pH 6.13	10	0	90
6	R.5-NaSCN	15	0	85
7	R.6-NaCl	62	25	13
8	R.7-TCEP	51	25	23
9	R.8-NDSB 201	40	32	28
10	R.9-Zwitt 3-12	31	29	39
11	R.10-Glycerol	52	3	46
12	R.11-PEG 4000	38	26	35
13	R.12-D-Sucrose	44	31	25
14	R.13-L-Arginine	44	44	12
15	R.14-L-Histidine	74	0	26
16	R.15-Betaine	71	0	29
17	R.16-L-Lysine	33	0	67
18	R.17-L-Proline	76	0	24
**Best combined conditions**				
19	R.18-L-Pro/NaCl	66	0	34
20	R.19-L-Pro/D-Suc	62	0	38
21	R.20-L-Pro/NaCl/D-Suc	80	0	20
22[B]	R.20-L-Pro/NaCl/D-Suc	85	0	15

[A]Protein unfolded with 4 M GuSCN. [B]Refolding buffer was added in a single pulse.

Supplementary Table 3: Refolding yields obtained under the different renaturing solutions explored during the screening. The yield of properly refolded protein is given in percent.

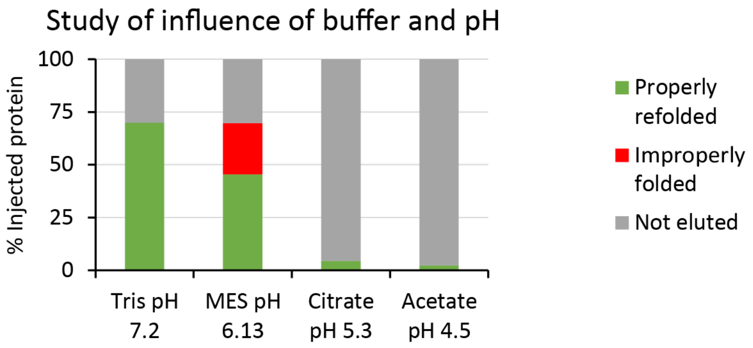

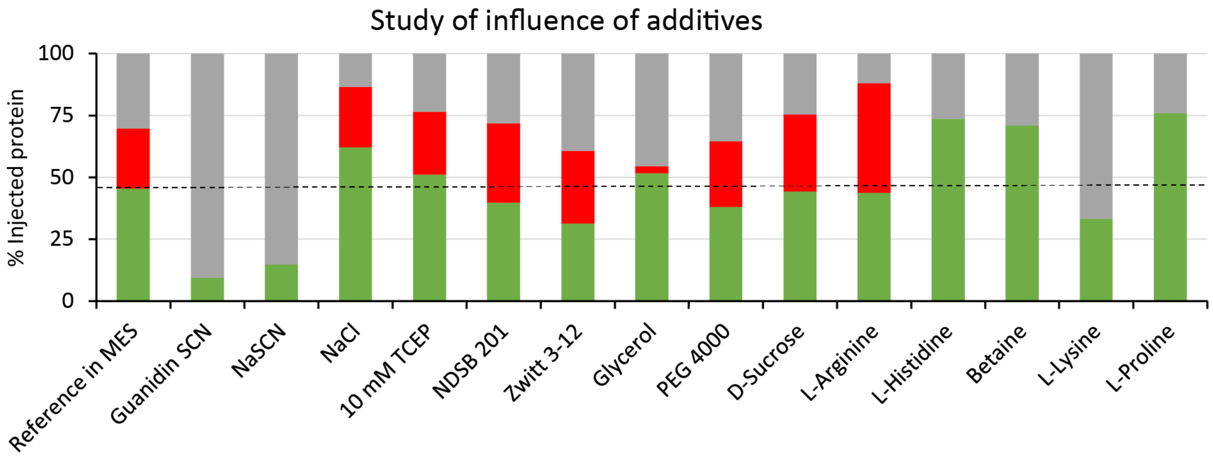

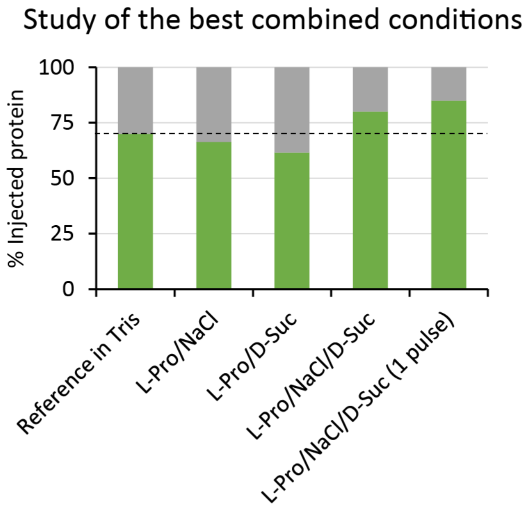

Supplementary Figure 2: Barplots showing the results of the refolding screening for Saga P-dimers as asessed by RP-HPLC. The influence of buffer and pH (top), commonly used refolding additives (center) and combinations of some additives (bottom) are displayed. Green, red and grey indicates properly refolded, improperly folded and not eluted protein, respectively. Dashed lines indicate the percentage of properly folded protein in the reference samples.

1.7 *Evaluation of Saga P-dimers selectivity towards HBGAs after unfolding/refolding protocol and quantification of D/H exchange*

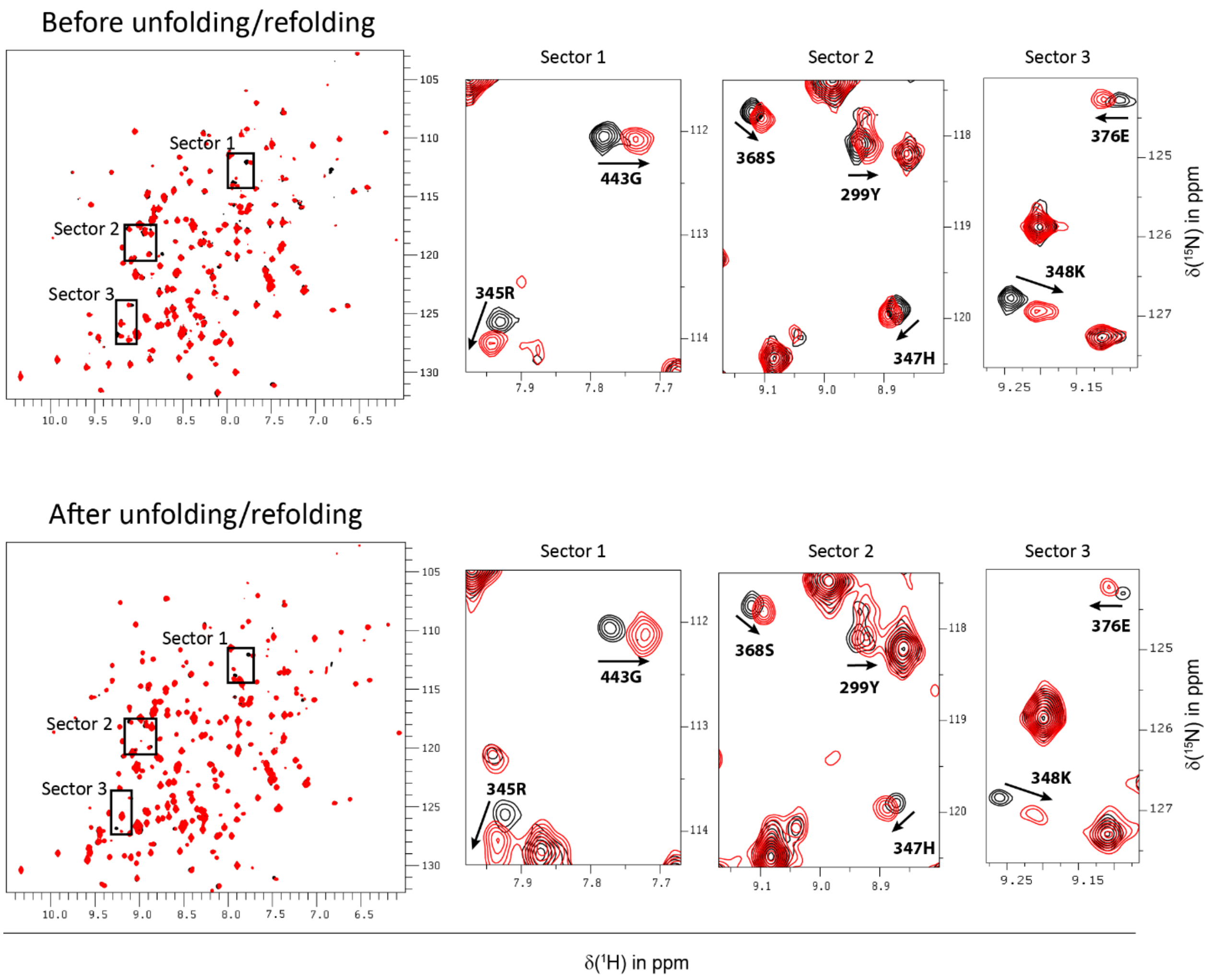

Supplementary Figure 3: Refolded *GII.4* Saga P dimers show unaltered HBGA recognition. $^{1}H,^{15}N$ TROSY HSQC spectra of 100 µM [U-$^{2}H,^{15}N$] *GII.4* Saga P-dimers in the absence (black) and in the presence (red) of 4 mM B-tetrasaccharide Type I before (top) and after (bottom) unfolding/refolding. Samples were prepared in 75 mM sodium phosphate buffer (pH 7.30) containing 100 mM NaCl, 100 µM DSS-*d6* NaCl, and 10% D_2O.

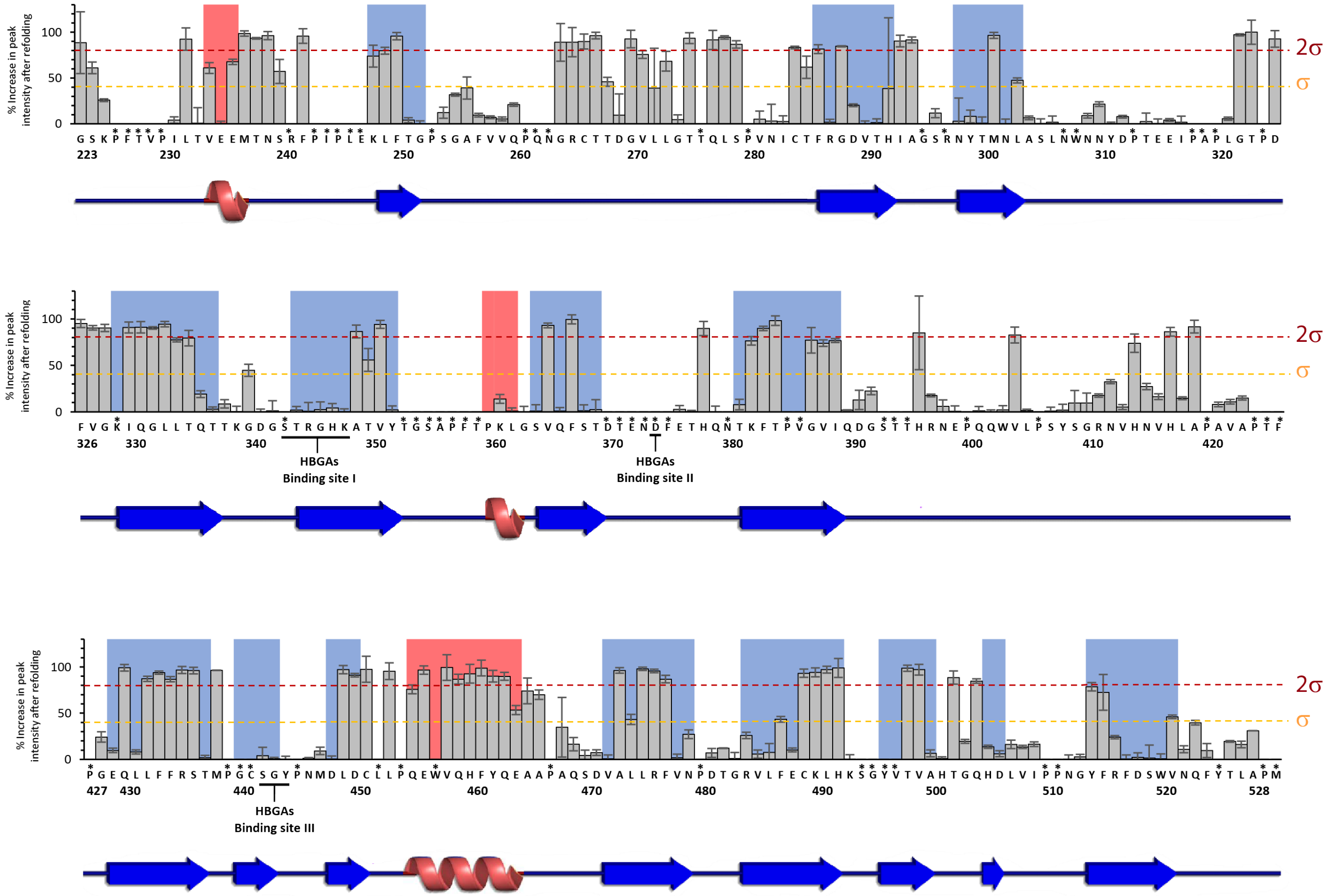

Supplementary Figure 4: Increase on peak intensity for $^{1}H,^{15}N$ backbone amide cross-peaks in $^{1}H,^{15}N$ TROSY HSQC spectra after protein unfolding and refolding aligned with the secondary structure of *GII.4* Saga P monomer (PDB code 4X06). Increase in peak intensities were calculated from two consecutive measurements per sample as the difference in the average intensities before and after protein refolding normalized to the highest peak (E316). Errors were calculated as the standard deviation from two consecutive measurements. Residues for which backbone assignments are not available are indicated by asterisks. Red and orange dashed lines indicate an increase over 80% (2σ) and 40% (σ) in peak intensity, respectively. α-helices and β-sheets are shown in red and blue, respectively. Canonical HBGAs binding sites for corresponding P-dimers are indicated as reported by ref[3].

1.8 Final D/H exchange protocol:

In order to reduce protein precipitation, all lab equipment and solutions were pre-cooled at 4 - 6 °C at least one hour prior to use. Each step was conducted at 4 - 6 °C.

Preparation of buffers and protein:

1. **Preparation of P domains for unfolding/refolding protocol:**

   P domains were transferred into 20 mM sodium phosphate buffer (pH 7.30) and concentrated to a final 7.0 mg per ml P-dimers concentration.

2. **Preparation of buffers and solutions:**

   The following quantities have been calculated for 1 ml at 7.0 mg per ml P-dimers solution.

   Unfolding solution:

   Dissolve 13.4 g GuHCl, 2.1 g Tris and 0.61 g NaCl in 30 ml MP-H_2O. Calibrate the pH to 7.30 using HCl 25%, add MP-H_2O up to a final 35 ml volume, sterile filter the solution and store at 4 °C. Add 24 µl of 2-mercaptoethanol immediately prior to use.

   Stabilizing solution:

   Dissolve 10.0 g GuHCl, 2.1 g Tris, 4.0 g L-proline and 4.8 g of D-sucrose in 30 ml MP-H_2O. Calibrate the pH to 7.30 using HCl 25%, add MP-H_2O up to a final 35 ml volume, sterilely filter the solution and store at 4 °C. Add 24 µl of β-mercaptoethanol immediately prior to use.

   Dialysis buffers:

   Refolding 1:

   Dissolve 121.1 g Tris, 115.1 g L-proline and 136.9 g D-sucrose in 1.7 l MP-H2O, calibrate the pH to 7.30 using HCl 25% and add MP-H_2O up to a final 2 l volume. Store at 4 °C. Add 1.37 ml of β-mercaptoethanol immediately prior to use.

   Refolding 2:

   Dissolve 60.6 g Tris, 57.6 g L-proline and 68.5 g D-sucrose in 1.7 l of MP-H_2O, calibrate the pH to 7.30 using HCl 25% and add MP-H_2O up to a final 2 l volume. Store at 4 °C. Add 0.68 ml of β-mercaptoethanol immediately prior to use.

Washing:

Dissolve 36.4 g Tris and 157.8 g NaCl in 5.7 L MP-H_2O, calibrate the pH to 7.30 using HCl 25% and add MP-H_2O up to a final 6 l volume. Divide into 3 vessels of 2 l each and store at 4 °C. Add 0.42 ml of β-mercaptoethanol per 2 l immediately prior to use.

Unfolding/refolding protocol:

1. **Protein unfolding:**

   The solution containing the P domains was transferred into a vessel large enough to accommodate 2x the volume of the unfolding solution. The mixture was incubated at 4 °C for 4 h. The vessel should not be moved or agitated during this first step.

2. **Refolding:**

   The stabilizing solution was slowly added to the previous solution, and the mixture was stepwise dialyzed against the following buffers at 4 °C (Supplementary Table 4). **Important:** Use a dialysis bag with a volume 2x the volume loaded. Due to osmotic pressure the volume in the bag will double during the procedure.

Order	Dialysis buffer	Volume (L)	Time
1	Refolding 1	2	Overnight
2	Refolding 2	2	8 h
3	Washing	2	Overnight
4	Washing	2	8 h
5	Washing	2	48 h

Supplementary Table 4: Stepwise dialysis scheme

3. **Protein purification:**
   P domains were concentrated to a volume of 5-8 ml using 15 ml Vivaspin filters (MWCO 30 kDa), purified by size-exclusion chromatography in 20 mM sodium phosphate buffer (pH 7.30) as described in Methods section of the main text and stored at 4 °C. Average yield: 81 %.

2. Supplementary note 2: NMR experiments

NMR spectra were recorded either on a Bruker AV III 500 MHz NMR spectrometer equipped with a TCI cryogenic probe (University of Lübeck), or on Bruker 600 MHz and 900 MHz machines located at the Bijvoet Center in Utrecht (The Netherlands). If not specified otherwise spectra were recorded at 298 K.

2.1 Optimization of sample conditions for 3D NMR experiments

A 160 µl sample containing 600 µM of refolded [*U*-^{2}H,^{15}N] Saga P-dimers was prepared in buffer consisting of 20 mM sodium phosphate (pH 7.30), 100 µM DSS-*d6*, and 10% D_2O. The sample was diluted stepwise to protein concentrations of 500, 400, 300, 200, 100 and 50 µM adding the same buffer. At each concentration the sample was equilibrated for 20 min at 298 K followed by the acquisition of a 1D ^{1}H NMR, ^{1}H,^{15}N TROSY HSQC, 1D ^{1}H,^{15}N TRACT and five 1-1 echo 1D NMR experiments. All experiments were completed within a single day. The same protocol employing selected protein concentrations was applied at lower pH values and for buffer containing 75 mM sodium phosphate and 100 mM NaCl (pH 7.30). Precipitation was observed at protein concentrations of 500 µM and 600 µM only for the high salt buffer.

2.1.1 ^{1}H and ^{1}H,^{15}N TROSY HSQC

^{1}H,^{15}N TROSY HSQC spectra were acquired on a Bruker AVIII 500 (see above). A standard phase sensitive pulse sequence from the Bruker pulse program library (trosyetf3gpsi.2) was used with 32 dummy scans, 8 scans (experiment time: 1 h and 12 min) and a spectral resolution of 15.6 Hz and 15.8 Hz for the direct and the indirect dimension, respectively. The frequency offset and spectral width were 4.69 ppm and 16.0 ppm, respectively, for the ^{1}H dimension and 118.5 ppm and 40.0 ppm, respectively, for the ^{15}N dimension. The receiver gain was kept constant at 256 through all the experiments allowing for a direct comparison of peak intensities. Spectra were processed with TopSpin 4.0.2 and analyzed with the CCPNMR 2.4.2 software suite[4].

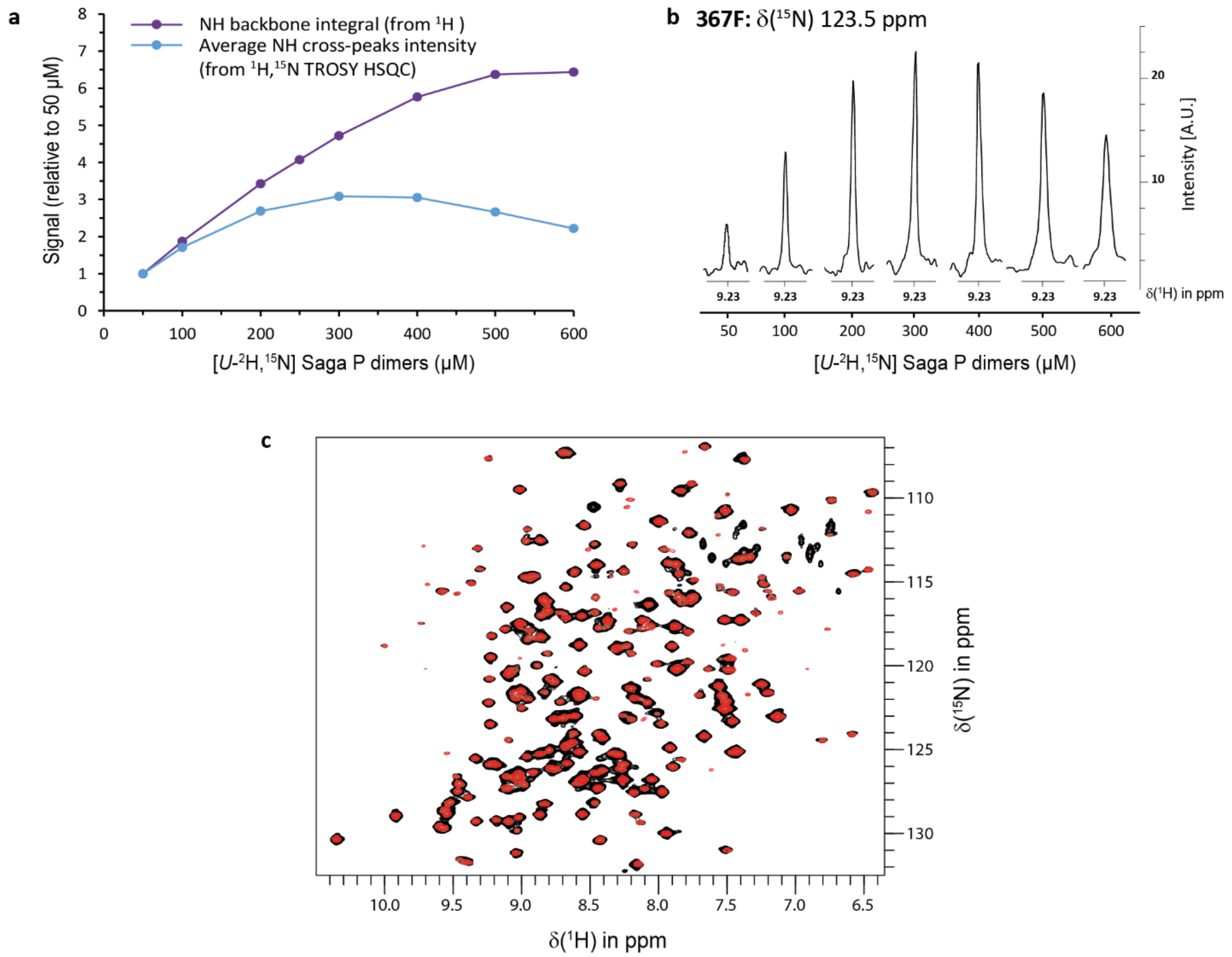

Supplementary Figure 5: *GII.4* Saga P dimers aggregate over 200 µM protein conc**. a)** Evolution of NMR signal with increasing Saga P-dimer concentrations. Violet corresponds to the sum integral over the range of 10.5 – 6 ppm in the ^{1}H NMR spectrum. Blue corresponds to the average NH cross-peak intensity in ^{1}H,^{15}N TROSY HSQC experiments. **b)** ^{1}H,^{15}N TROSY HSQC cross-section corresponding to amino acid 367F at increasing protein concentrations. A decrease in peak intensity accompanied by line broadening can be observed at concentrations higher than 300 µM Saga P-dimers. **C)** Section of ^{1}H,^{15}N TROSY HSQC spectra of samples containing 100 µM (red) and 600 µM (black) [*U*-^{2}H,^{15}N] *GII.4* Saga P-dimers. No CSPs due to protein-protein interactions could be observed. Spectra were recorded using 20 mM sodium phosphate buffer at pH 7.30 (see text).

2.1.2 1D [^{15}N, ^{1}H]-TRACT experiments

Effective rotational correlation times (τ_c) were calculated from [^{1}H,^{15}N]-TRACT experiments using the pulse sequence described in ref.[5]. 25 relaxation delays (Δ_i) were used to obtain corresponding magnetization decay curves: 0, 4, 8, 12, 16, 20, 24, 28, 32, 36, 40, 50, 40, 70, 80, 100, 120, 140, 180, 200, 240, 300, 360, and 400 ms, with 16 transients per Δ_i value (16 min). The frequency offset and spectral width were 4.69 ppm and 16.0 ppm, respectively. 1024 points were acquired per experiment, with a FID resolution of 7.82 Hz. Two experiments with phase cycle-selection either for the α- or for the β-spin states of ^{15}N were acquired consecutively. Spectra were processed with TopSpin 4.0.2, and R_α and R_β relaxation rates were obtained by independently fitting Supplementary Equation (2) to the decaying α- or β-spin signal intensities *I* using the Origin 2016G software package.

$$I = e^{(-R_{\alpha,\beta}\Delta_i)} \quad (2)$$

Finally, τ_c was calculated as the difference between R_β and R_α using Supplementary Equation (3) (taken from ref.[5]):

$$R_\beta - R_\alpha = 2\frac{\mu_0\gamma_H\gamma_N h}{16\pi^2\sqrt{2}r_{HN}^3} * \frac{\gamma_N B_0 \Delta\delta_N}{3\sqrt{2}} * \left(1.6\tau_C + \frac{1.2\tau_C}{1+\tau_C^2\omega_N^2}\right) * 3\cos^2(\theta - 1) \quad (3)$$

Where μ_0 is the permeability of the vacuum, γ_H and γ_N are the gyromagnetic ratios of ^{1}H and ^{15}N, respectively, h is the Plank constant, r_{HN} is the ^{15}N-^{1}H internuclear distance (1.02 Å), B_0 is the external magnetic field strength, $\Delta\delta_N$ is the difference of the two principal components of the axially symmetric ^{15}N chemical shift tensor (160 ppm), and ω_N is the angular Larmor frequency of ^{15}N at a given B_0. Θ was assumed to be 17°.

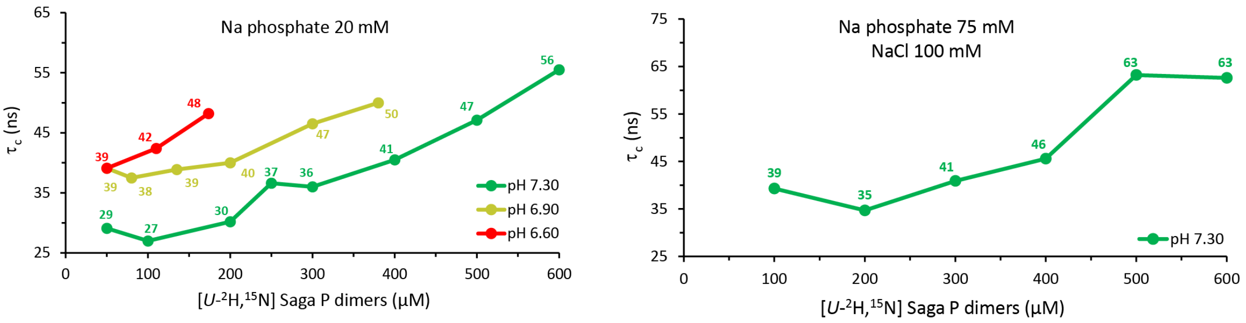

Supplementary Figure 6: Effective rotational correlation times (τ_c) calculated from ^{1}H,^{15}N TRACT experiments for refolded [*U*-^{2}H,^{15}N]-Saga P-dimers in 20 mM sodium phosphate buffer (*left*) and in 75 mM sodium phosphate buffer with 75 mM NaCl (*right*). Only the spectral region corresponding to well-structured protein secondary motifs (9.8 – 8 ppm) was integrated in order to reduce the influence of flexible residues on the measured τ_c. All experiments were acquired at 298 K and 500 MHz.

2.1.3 Spin-echo NMR experiments

Cumulative transverse relaxation times T_2 of backbone H^N signals were measured using 1-1 echo 1D NMR experiments[6] from refolded [*U*-^{2}H,^{15}N] Saga P-dimers at 500 MHz and 298 K. All samples contained 100 µM DSS-*d6* as internal reference and 10% D_2O. A delay Δ was introduced to allow for measuring signal decay due to transverse relaxation. The relaxation of transverse magnetization follows Supplementary Equation (4):

$$-R_{xy}t = \ln\left(\frac{M_x(t)}{M_x(0)}\right) \quad (4)$$

Where R_{xy} is the rate constant for transverse relaxation, M_x is the x-magnetization, and t is time. This equation can be rewritten as Supplementary Equation (5):

$$R_{xy}2(\Delta_A - \Delta_B) = -\ln\left(\frac{I_{\Delta A}}{I_{\Delta B}}\right) \quad (5)$$

Where $I_{\Delta A}$ and $I_{\Delta B}$ are the respective intensities of the cumulative H^N signals for two arbitrary delays Δ_A and Δ_B, and *t* is defined as twice the difference between two delays according to the pulse sequence. Using five different delays Δ of 0.1, 0.8, 1.5, 2 and 3 ms, backbone H^N relaxation times T_2 were obtained as the inverse of the slope of the fitted natural logarithm of the intensity ratios to the corresponding difference of delays according to Eq. 5 (see Supplementary Figure 7 and Supplementary Table 5). The effect of protein concentration, pH and salt content on backbone H^N transverse relaxation times T_2 for [U-^{2}H,^{15}N] Saga P-dimers is summarized in Supplementary Figure 8.

Δ_A (ms)	Δ_B (ms)	$2(\Delta_A-\Delta_B)$ (ms)	$-\ln(I_{\Delta A}/I_{\Delta B})$
0.8	0.1	1.4	0.205
2	0.8	2.4	0.293
1.5	0.1	2.8	0.390
2	0.1	3.8	0.507
3	0.8	4.4	0.527
3	0.1	5.8	0.732

Supplementary Table 5: Parameters for measurement of T_2.

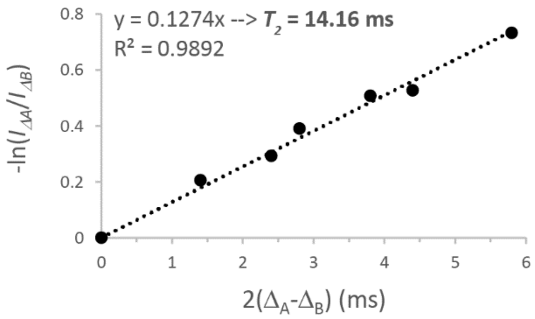

Supplementary Figure 7: Example showing the procedure used to calculate backbone H^N T_2 from 1-1 echo 1D NMR experiments. Six differences of delays $2(\Delta_A-\Delta_B)$ were calculated from the five Δ_i delays acquired, and Supplementary Equation (5) was fitted to the corresponding experimental values of $-\ln(I_{\Delta A}/I_{\Delta B})$. $R^2 > 0.96$ were observed for all fits.

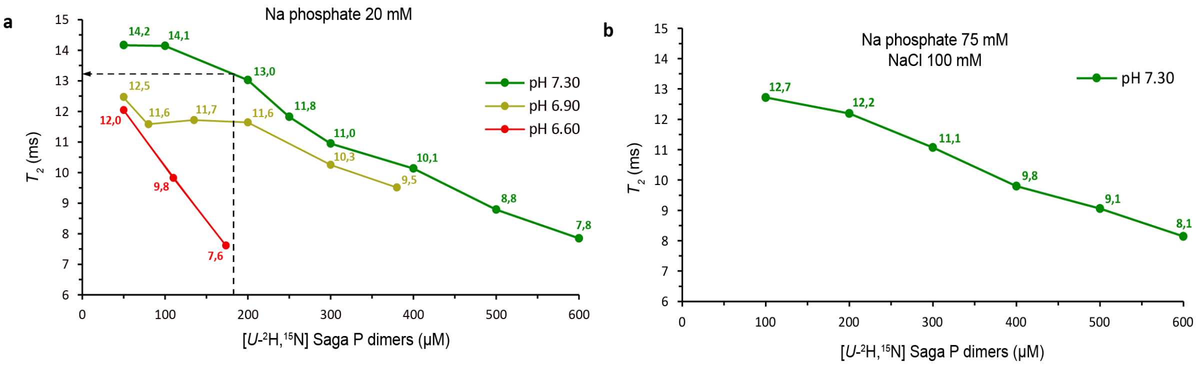

Supplementary Figure 8: Cumulative transverse relaxation times T_2 of backbone H^N signals obtained for Saga P-dimers in **a)** 20 mM sodium phosphate buffer and in **b)** in 75 mM sodium phosphate buffer containing 100 mM NaCl. The dashed line at a concentration of Saga P-dimers at 180 µM in panel **a)** indicates the shortest H^N backbone T_2 (ca. 13.3 ms) required for complete magnetization transfer in out-and-back HN(CO)CACB NMR experiments for 20 mM sodium phosphate buffer, pH 7.30 at 500 MHz.

2.1.4 Dynamic light scattering (DLS)

DLS was used as an alternative technique to investigate the influence of salt and protein concentrations on potential protein aggregation. Before measurement, samples were centrifuged for 10 min at 4°C at 16,100x*g*. Measurements were performed in a Laser-Spectroscatter 201 (RiNA GmbH) using a pre-specified solvent profile for water. Viscosity and refractive index values of 1.007 cP and 1.65, respectively, were used. DLS measurements were

performed at 22 °C at a scattering angle of 90°. For each sample, 10 consecutive readings over 30 s intervals were recorded. Two samples containing 600 µM and 400 µM Saga P-dimers were prepared in 20 mM sodium phosphate buffer and in 75 mM sodium phosphate containing 100 mM NaCl. Samples were incubated for 1 h at 298 K., centrifuged and then measured. Samples were then diluted to half concentration, incubated for 1 h at 298 K, centrifuged and DLS measurements were repeated under identical conditions. Heat maps and mean radius distribution diagrams were created by the DLS software supplied along with SpectroSize^{TM}300 (Supplementary Figure 9).

Na phosphate 20 mM, pH 7.30:

A) 300 µM Saga P dimers

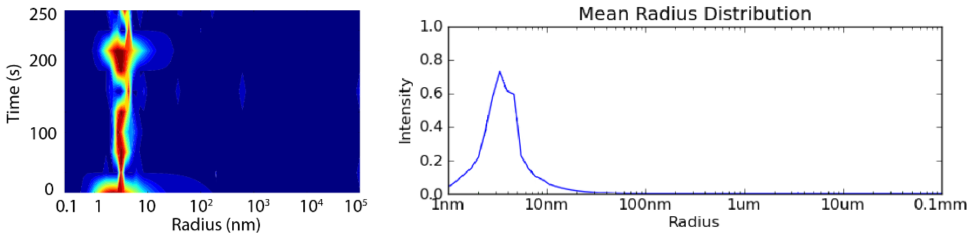

B) 600 µM Saga P dimers

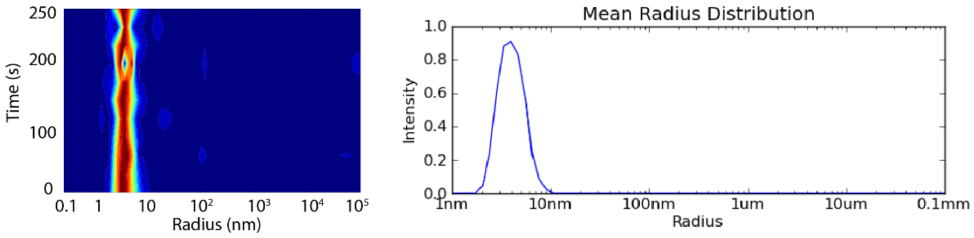

Na phosphate 75 mM, NaCl 100 mM, pH 7.30:

A) 200 µM Saga P dimers

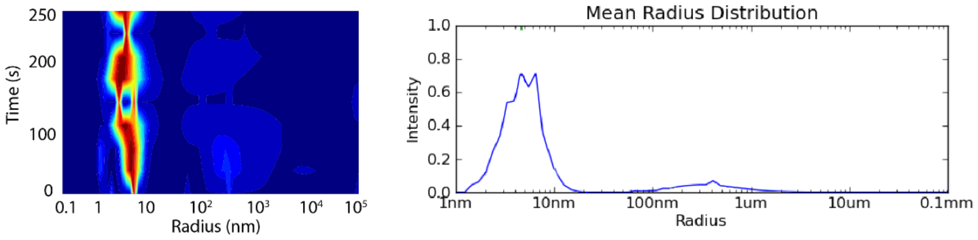

B) 400 µM Saga P dimers

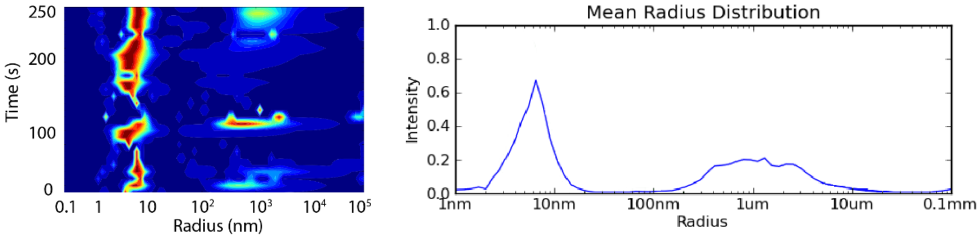

Supplementary Figure 9: Heat maps and mean radius distribution diagrams obtained from 10 measurements for Saga P-dimer samples at high and low protein concentrations and at high and low salt concentrations. The heat maps reveal a hydrodynamic radius of 5 nm for Saga P-dimers when diluted in 20 mM sodium phosphate. At high salt concentration, Saga P-dimers form higher molecular weight aggregates in a protein concentration dependent manner.

2.2 3D NMR spectroscopy

2.2.1 Experimental parameters

Experiment	Pulse program (Bruker)	Field	F3 (TD; O1; SW)	F2 (TD; O1; SW)	F1 (TD; O1; SW)	NS
**HNCO**	trhncogp3d	900 MHz	1024;<br>4.71 ppm;<br>14 ppm	64;<br>117 ppm;<br>31 ppm	128;<br>176 ppm;<br>18 ppm	16
**HN(CA)CO**	trhncacogp3d	900 MHz	2048;<br>4.71 ppm;<br>14 ppm	64;<br>117 ppm;<br>31 ppm	96;<br>176 ppm;<br>18 ppm	32
**HN(CA)CO**	trhncacogp2h3d	500 MHz	2048;<br>4.65 ppm;<br>13 ppm	64;<br>117.5 ppm;<br>32 ppm	128;<br>174 ppm;<br>16 ppm	32
**HNCA**	trhncagp2h3d2	900 MHz	2048;<br>4.65 ppm;<br>13 ppm	64;<br>117 ppm;<br>31 ppm	128;<br>54 ppm;<br>30 ppm	32
**HN(CO)CA**	trhncocagp2h3d	500 MHz	2048;<br>4.71 ppm;<br>43 ppm	64;<br>117.5 ppm;<br>32 ppm	128;<br>54 ppm;<br>30 ppm	16
**HNCACB**	trhncacbetgp2h3d	900 MHz	2048;<br>4.71 ppm;<br>14 ppm	64;<br>117 ppm;<br>31 ppm	96;<br>40 ppm;<br>80 ppm	32
**HN(CO)CACB**	trhncocacbetgp2h3d	900 MHz	2048;<br>4.71 ppm;<br>14 ppm	64;<br>117 ppm;<br>31 ppm	128;<br>40 ppm;<br>80 ppm	16
**HN(CO)CACB**	trhncocacbgp2h3d	500 MHz	2048;<br>4.65 ppm;<br>13 ppm	64;<br>117.5 ppm;<br>32 ppm	128;<br>40 ppm<br>80 ppm	16

Supplementary Table 6: Compilation of 3D experiments for backbone assignment. TD denotes the number of increments, O1 is the center of the spectral window, SW is the sweep width, and NS is the number of transients. The relaxation delay was set to 1 s for all experiments.

2.2.2 Chemical shift-based secondary structure prediction

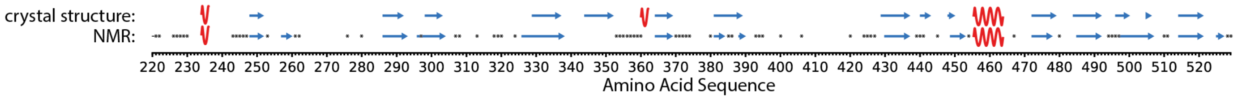

Supplementary Figure 10: Comparison of secondary structure elements as found in a respective crystal structure of *GII.4* Saga P-dimers (pdb: 4x06) and as predicted using the TALOS-N web server based on NMR chemical shift information. α-helices are depicted in red, β-sheets in blue. Asterisks denote residues where no chemical shift information was available.

2.2.3 Backbone amide chemical shifts of deamidated Saga P-dimers

Residue	Amino acid	δ(^{1}H) in ppm	δ(^{15}N) in ppm
**290**	Val	9.76	112.9
**293**	Ile	7.84	129.8
**294**	Ala	8.24	132.2
**296**	Ser	7.87	115.9
**298**	Asn	8.56	119.4
**299**	Tyr	9.03	118
**300**	Thr	9.01	121.5
**301**	Met	9.58	128.6
**302**	Asn	8.87	128.2
**329**	Lys	9.8	125.9
**330**	Ile	8.65	124.1
**333**	Leu	7.8	122.5
**334**	Leu	8.89	126.2
**335**	Thr	9.33	114.4
**336**	Gln	8.85	121.5
**337**	Thr	7.1	122.6
**339**	Lys	8.4	130.1
**345**	Arg	7.94	114.2
**347**	His	8.82	118.4
**349**	Ala	8.78	128.8
**350**	Thr	8.84	117
**367**	Phe	9.24	123.3
**368**	Ser	9.11	116.7
**369**	Thr	8.17	115.3
**370**	Asp	8.71	118.9
**371**	Thr	7.53	111
**372**	Glu	8.18	122.1
**373**	Asn	8.41	125.4
**374**	Asp	8.16	126
**375**	Phe	7.23	117.1
**376**	Glu	8.91	123
**377**	Thr	8.63	114.5
**378**	His	9.56	115.4
**381**	Thr	9.5	125.1
**382**	Lys	9.3	127.8
**383**	Phe	9.42	127
**388**	Val	8.88	112.5
**442**	Ser	7.06	116
**443**	Gly	7.73	112.1
**446**	Asn	8.28	118.9

Supplementary Table 7: Backbone amide chemical shifts of deamidated Saga P-dimers in 20 mM sodium phosphate buffer, pH* 7.30 as identified during the assignment process. Note, that only those residues have been listed where signals could be clearly distinguished from those of the non-deamidated protein.

2.3 Chemical shift titrations

2.3.1 NMR experimental conditions

$^{1}H,^{15}N$ TROSY HSQC spectra were acquired on a Bruker AVIII 500 (see above). A standard phase sensitive pulse sequence from the Bruker pulse program library was used with 32 dummy scans and 20 scans (3 h 52 min). The frequency offset and spectral width were set at 4.69 ppm and 16.0 ppm for the ^{1}H dimension and 118.5 ppm and 40.0 ppm for the ^{15}N dimension, respectively. A total of 2048 x 430 points were acquired for the direct and indirect dimensions, respectively. Spectra were processed using TopSpin 4.0.2. Data were apodized with a Qsine window function. FIDs were zero-filled to give a final 4096 x 1024 data matrix prior to Fourier-transformation. Spectra were manually phased and peak positions were automatically extracted using a local extrema algorithm with a non-diagonal search within CCPNMR 2.4.2 software suite.

2.3.2 Quantification of chemical-shift perturbations

Combined chemical shift perturbations (CSPs) were calculated as Euclidean distances in Hz according to Supplementary Equation (6) (see ref.[7]):

$$\Delta v_{\mathrm{eucl}}\ (\mathrm{Hz}) = \sqrt{(\Delta^{1}H * 500)^{2} + (\Delta^{15}N * 50.66)^{2}} \qquad (6)$$

Where Δv are the combined CSPs, and $\Delta^{1}H$ and $\Delta^{15}N$ are the differences of ^{1}H and ^{15}N chemical shifts, respectively, between the first point and the nth point of a titration.

2.3.3 Minimum threshold for significant chemical shift perturbations

Modern spectrometers allow the measurement of small chemical shift perturbations from $^{1}H,^{15}N$ HSQC and $^{1}H,^{13}C$ HMQC NMR experiments with high accuracy and precision, as it has been shown recently [8, 9, 10]. Differences in peak position in two consecutively acquired spectra of the same sample under identical conditions can be introduced during data acquisition due to small variations in sample conditions such as pH drift, volume variation, or temperature instability. Differences may also be due to difficulties in reproducibly determining peak positions of broadened or overlapping signals, especially when using peak picking algorithms. Obviously, precision is the critical factor for the significance of small chemical shift perturbations. A recent study has shown that a precision of 0.004 ± 0.003 ppm (combined chemical shift differences, which translates into ca. 2.8 ± 2.1 Hz) can be achieved for four identical samples at 500 MHz, being only slightly lower than the combined digital resolution[11]. Yet another study found an experimental precision from duplicate experiments of 0.004 ppm at 900 MHz[12]. In our NMR experiments the theoretical limit of precision in Euclidean distances is 2.10 Hz (combined spectral resolution). However, a simple comparison of consecutively acquired $^{1}H,^{15}N$ TROSY HSQC spectra with the same sample shows random fluctuations in peak positions larger than this theoretical threshold. Therefore, it was necessary to experimentally

determine the precision of our measurements defining an appropriate threshold for the determination of significant CSPs from ligand titrations and/or amino acid substitutions.

To quantify the precision for our NMR measurements we prepared a 160 µl sample containing 110 µM of refolded [*U*-^{2}H,^{15}N] Saga P-dimers in 75 mM sodium phosphate buffer, pH 7.30, containing 100 mM NaCl, 100 µM DSS-*d6*, 400 µM imidazole, and 10% D_2O. Ten ^{1}H,^{15}N TROSY HSQC spectra were consecutively acquired over two days. To better simulate errors introduced due to sample manipulation during ligand titration, the sample was removed from the magnet after every measurement, 2 µl of buffer were added, and the sample was reintroduced in the magnet and automatically locked, matched and tuned using IconNMR (Bruker). Spectra were processed in TopSpin 4.0.2, and peaks were automatically picked in CCPNMR 2.4.2. Peaks assigned for the wild-type species (NN P-dimers) were selected for the next step. Comparison of peak positions in the individual spectra yielded deviations, which were expressed as Euclidean chemical shift differences. Euclidean chemical shift differences were calculated for all possible pairwise permutations of the ten measurements for a total of 220 peaks, affording a 220 x 45 noise matrix. A histogram showing the distribution of the size of the Euclidean chemical shift differences is shown in Supplementary Figure 11. Fitting a generalized extreme value (GEV) function (Supplementary Equation (7)) to the experimental data[13, 14] provided the probability density function associated with the distribution of the error observed in the measurements (red curve in Supplementary Figure 11).

$$y = f(x|k,\mu,\sigma) = \begin{cases} \left(\frac{1}{\sigma}\right)\exp\left(-\left(1+k\frac{(x-\mu)}{\sigma}\right)^{-\frac{1}{k}}\right)\left(1+k\frac{(x-\mu)}{\sigma}\right)^{-1-\frac{1}{k}} & \text{for } k \neq 0 \\ \left(\frac{1}{\sigma}\right)\exp\left(-\exp\left(-\frac{(x-\mu)}{\sigma}\right)-\frac{(x-\mu)}{\sigma}\right) & \text{for } k = 0 \end{cases} \quad (7)$$

Where μ, σ and k are the scale, location and shape parameter, respectively. The location parameter σ must be > 0, whilst the other two parameters can take any real value. *y* denotes the frequency of the Euclidean distance, and *x* is the Euclidean distance. Of note, the definition range of the GEV distribution function depends on *k* according to Supplementary Equation (8), (9):

$$1 + k\frac{(x-\mu)}{\sigma} > 0 \qquad \text{for } k \neq 0 \quad (8)$$

$$-\infty < x < +\infty \qquad \text{for } k = 0 \quad (9)$$

For our noise matrix the parameters were μ = 0.520515, σ = 0.427997 and k = 0.38217. We have used a corresponding cumulative density function (blue curve in Supplementary Figure 11) to define the significance level for Euclidean chemical shift differences. Setting the

confidence level at 99.5% leads to a significance threshold of 7.88 Hz (Arrow in Supplementary Figure 11). All calculations were performed in Matlab R2016b. It is important to note that this methodology only applies to processes (ligand binding or conformational transitions) that are fast on the chemical shift time scale. This is the case for the vast majority of peaks in *GII.4* P-dimers.

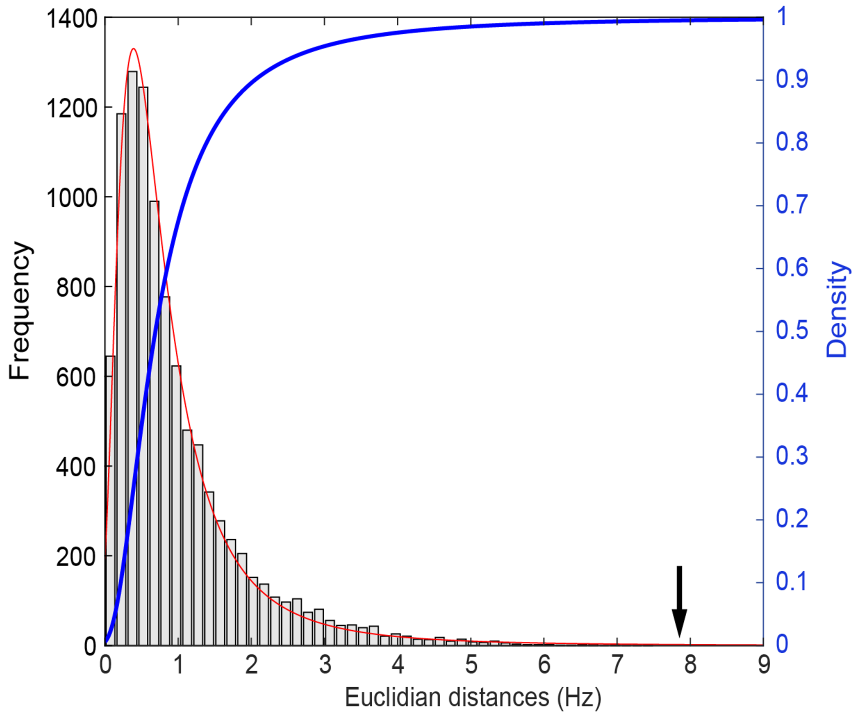

Supplementary Figure 11: Bar plot showing the histogram of the noise matrix, the GEV probability density function (red, left axis) and the corresponding cumulative density function (blue, right axis). The black arrow indicates the 99.5% confidence level.

2.3.4 Sample preparation and HBGA titrations

Freshly expressed and unfolded/refolded [*U*-^{2}H,^{15}N] Saga P-dimers containing a minimum 92% of NN P-dimer species was used for titrations over NN P-dimers. For titrations over iDiD P-dimers, NN P-dimers were allowed to deamidate at 25 °C until less than 5% of iDN and NN P-dimer species were observed (as quantified by IEX). Imidazole signals were calibrated and used as internal pH reporter as described previously[15].

2.3.5 Titrations with methyl α-L-fucopyranoside

Two 160 µl NMR samples were prepared in 75 mM sodium phosphate buffer at pH 7.30 and 100 mM NaCl, containing 200 µM of DSS-*d6*, 300 µM imidazole, 0.04% NaN_3, 10% D_2O and 100 µM of NN or iDiD refolded [*U*-^{2}H,^{15}N] Saga P-dimers, respectively.

For NN P-dimers, methyl α-L-fucopyranoside was titrated at 20 increasing concentrations corresponding to 0, 0.5, 1, 2, 3, 4, 5, 6, 7, 8, 9, 10, 11, 12, 15, 20, 40, 80, 120 and 160 mM. At the end of the titration, the content of NN P-dimer species was over 70% (as estimated from the 2D ^{1}H,^{15}N-HSQC-TROSY NMR spectrum), which still allows for a clear peak identification.

For iDiD P-dimers, methyl α-L-fucopyranoside was titrated in 17 increasing concentrations: 0, 1, 3, 5, 7, 9, 11, 13, 15, 20, 25, 30, 50, 90, 140, 180, 220 mM. The total protein concentration was kept constant during both titrations. The largest pH drift observed during the titrations was 0.03 and 0.04 pH units for the samples containing NN and iDiD P-dimers, respectively. pH titrations with [U-^{2}H,^{15}N] Saga P-dimers (data not shown) did not yield significant CSPs (> 7.88 Hz) for such small pH variations. Therefore, CSPs observed upon ligand binding are not affected by such small pH drifts.

2.3.6 Chemical shift perturbations in the presence of blood group B trisaccharide

Two 160 µl NMR samples containing either 115 µM of NN or 105 µM of iDiD refolded [U-^{2}H,^{15}N] Saga P-dimers were prepared in the same conditions as described for methyl α-L-fucopyranoside titrations. In both cases, samples were measured at 0 and at 20 mM B trisaccharide concentrations, keeping the protein concentration constant. No significant pH variation was observed after addition of B trisaccharide.

2.3.7 Noise reduction of chemical shift titration data

The ability of singular value decomposition (SVD) to identify orthogonal directions of maximum variance in large datasets makes it an excellent method to filter noise introduced during data acquisition and/or data processing[16, 17], and has also been applied by us[18]. Therefore, we used SVD for denoising ^{1}H,^{15}N-HSQC-TROSY spectra prior to CSP analysis as explained in the following.

Briefly, we determined chemical shifts of backbone NH signals, δ_{H} and δ_{N}, from the series of ^{1}H,^{15}N-HSQC-TROSY spectra with increasing amounts of methyl α-L-fucopyranoside. For each spectrum we created two one-dimensional column vectors, containing the δ_{H} and δ_{N} values $\mathbf{d} = \{\delta_{\mathrm{H}1}, \ldots, \delta_{\mathrm{H}m}, \delta_{\mathrm{N}1}, \ldots, \delta_{\mathrm{N}m}\}$, with m being the peak number in the spectrum. For a series of n ligand concentrations, a $2m$ x n chemical shift data matrix is obtained, representing the complete titration. Titrations employing NN and iDiD P-dimers were analyzed separately. In both cases, SVD analysis identified three non-noise components with autocorrelation factors over 0.5 and with a smooth behavior of the corresponding shapes of the $\mathbf{v}_i$ vectors (Supplementary Figure 12). The low or even negative autocorrelation factors for the fourth and larger components in both titrations clearly indicate noise. As an exception, the sixth component from the titration employing NN P-dimers shows a sizable autocorrelation factor of 0.53. However, based on the jagged shape of $\mathbf{v}_6$ and due to the low weight of its associated Eigenvalue (it represents only a 0.002% of the total data) we excluded this component from spectral reconstruction.

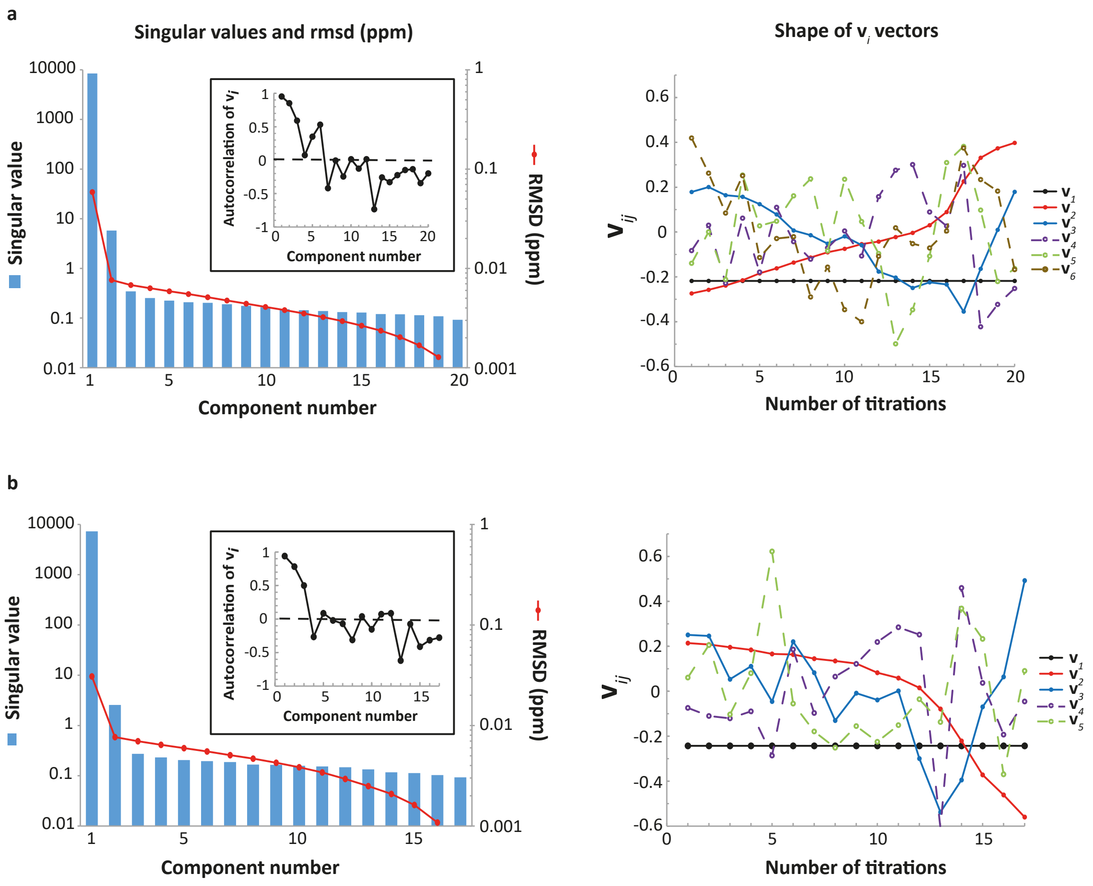

Supplementary Figure 12: Size and autocorrelation of individual components (*left*) and shape of the associated Eigenvectors (*right*) for the titration of methyl α-L-fucopyranoside titration to **a)** NN P-dimers and **b)** iDiD P-dimers.

Chemical shift datasets were reconstructed from the first three non-noise components, which represents 99.97% of the data in both titrations. The denoised datasets are practically identical to the raw datasets with a rmsd value of 0.007 ppm, corresponding to a maximum error of 3.52 Hz (Euclidean distances) far below the experimentally determined significance threshold of 7.88 Hz (cf. 3.3.3). ^{1}H-^{15}N CSPs were calculated from the denoised dataset as Euclidean distances in Hz according to Supplementary Equation (6). Only amino acids showing CSPs above the threshold of 7.88 Hz (measured at the highest methyl α-L-fucopyranoside concentration) were considered for further analysis.

2.3.8 Covariance analysis and hierarchical clustering of binding isotherms for the titration of NN P-dimers with methyl α-L-fucopyranoside

Binding isotherms resulting from the titration of methyl α-L-fucopyranoside to NN P-dimers exhibited visibly different shapes. Therefore, we grouped similar shapes in individual clusters, allowing separate analysis. As clustering by visual inspection is rather error prone we performed a covariance analysis, followed by hierarchical clustering.

Denoised CSPs (cf. 3.3.7) for each amino acid were arranged in an array $\underline{\Delta\nu}_i = \{\Delta\nu_{i,1}, \Delta\nu_{i,2}, \ldots, \Delta\nu_{i,n}\}$, with *n* being the titration points, corresponding to increasing methyl α-L-fucopyranoside concentrations, and *i* being the amino acid number. For *m* assigned amino acids showing significant CSPs a *m* x *n* matrix of CSPs is obtained. The cross-correlation between two residues *i* and *j* was calculated as the Pearson´s correlation coefficients (r_{ij}), which describes the covariance of two arrays $\underline{\Delta\nu}_i$ and $\underline{\Delta\nu}_j$ (cov_{ij}) normalized to the product of their standard deviations ($s_i s_j$) (Supplementary Equation (10)). The use of Supplementary Equation (10) has been described,[8] and here we explain its application to our case.

$$r_{ij} = \frac{\mathrm{cov}_{ij}}{s_i s_j} = \frac{\sum_{k=1}^{n}(\Delta v_{ik} - \overline{\Delta v}_i)(\Delta v_{jk} - \overline{\Delta v}_j)}{\sqrt{\sum_{k=1}^{n}(\Delta v_{ik} - \overline{\Delta v}_i)^2 \sum_{k=1}^{n}(\Delta v_{jk} - \overline{\Delta v}_j)^2}} \tag{10}$$

with $\overline{\Delta v}_i = \left(\frac{1}{n}\right)\sum_{k=1}^{n}\Delta v_{ik}$ and $\overline{\Delta v}_j = \left(\frac{1}{n}\right)\sum_{k=1}^{n}\Delta v_{jk}$ being the average values of $\Delta\nu_i$ and $\Delta\nu_j$, respectively, and *n* being the number of titration points. It is important to note that due to the normalization of the covariance with respect to the standard deviation product, the method is insensitive to the magnitude of the CSPs. As a result, the method allows to efficiently correlate not only the large CSPs observed close to ligand saturation (end of the titration curve), but also the small but critical CSPs that define the beginning of the titration curve and, therefore, its slope. As a logical consequence correlation coefficients (r_{ij}) decrease when the dataset contains large amounts of noise. This becomes important for the first points of the binding isotherm, representing small CSPs, close to the noise level, at low ligand concentrations. Therefore, the use of a denoised dataset was crucial for the analysis. Based on the distribution of p-values we set a threshold of α = 0.01 to exclude uncorrelated r_{ij} coefficients.

Using a threshold of 0.08, two clusters were identified *via* hierarchical agglomerative complete linkage clustering (Supplementary Figure 13). The shapes of individual curves can be inspected in Supplementary Figure 13. Cluster analysis was impossible for iDiD P-dimers due to much weaker CSPs and due to the inability to reach ligand saturation even at a concentration of 220 mM methyl α-L-fucopyranoside.

2.3.9 Non-linear least squares global fitting yields dissociation constants K_D

For NN P-dimers, we only considered the major cluster (cluster 1 in Supplementary Figure 13), comprising a total of 83 amino acids and, therefore, the same number of titration curves (Euclidean distances). K_D was determined by global non-linear least squares fitting of Supplementary Equation (11) to the dataset:

$$O = \frac{K_D + [L]_T + [P]_T - \sqrt{(K_D + [L]_T + [P]_T)^2 - 4[L]_T[P]_T}}{2[P]_T} O_{max} \quad (11)$$

with O being the observed CSP at each ligand concentration, $[P]_T$ and $[L]_T$ are the total protein and ligand concentrations, respectively, and O_{max} is the CSP at saturation with ligand.

For iDiD P-dimers, titration curves of all amino acids exhibiting significant CSPs were subjected to non-linear least squares fitting of Supplementary Equation (11). Fitting was done using Origin2016G (OriginLab). Results are summarized in Supplementary Table 8. Reduced χ^2-values are smaller than 1 because of the use of a denoised dataset. Compare also Fig. 4 of the main text.

**Protein form**	**Ligand**	***K*D (mM)**	**R^2**	**χ^2**
NN P-dimers	Methyl α-L-fucopyranoside	22.3 ± 0.04	0.9996	0.0948
iDiD P-dimers	Methyl α-L-fucopyranoside	219.8 ± 4.5	0.9995	0.0470

Supplementary Table 8: Dissociation constants K_D.

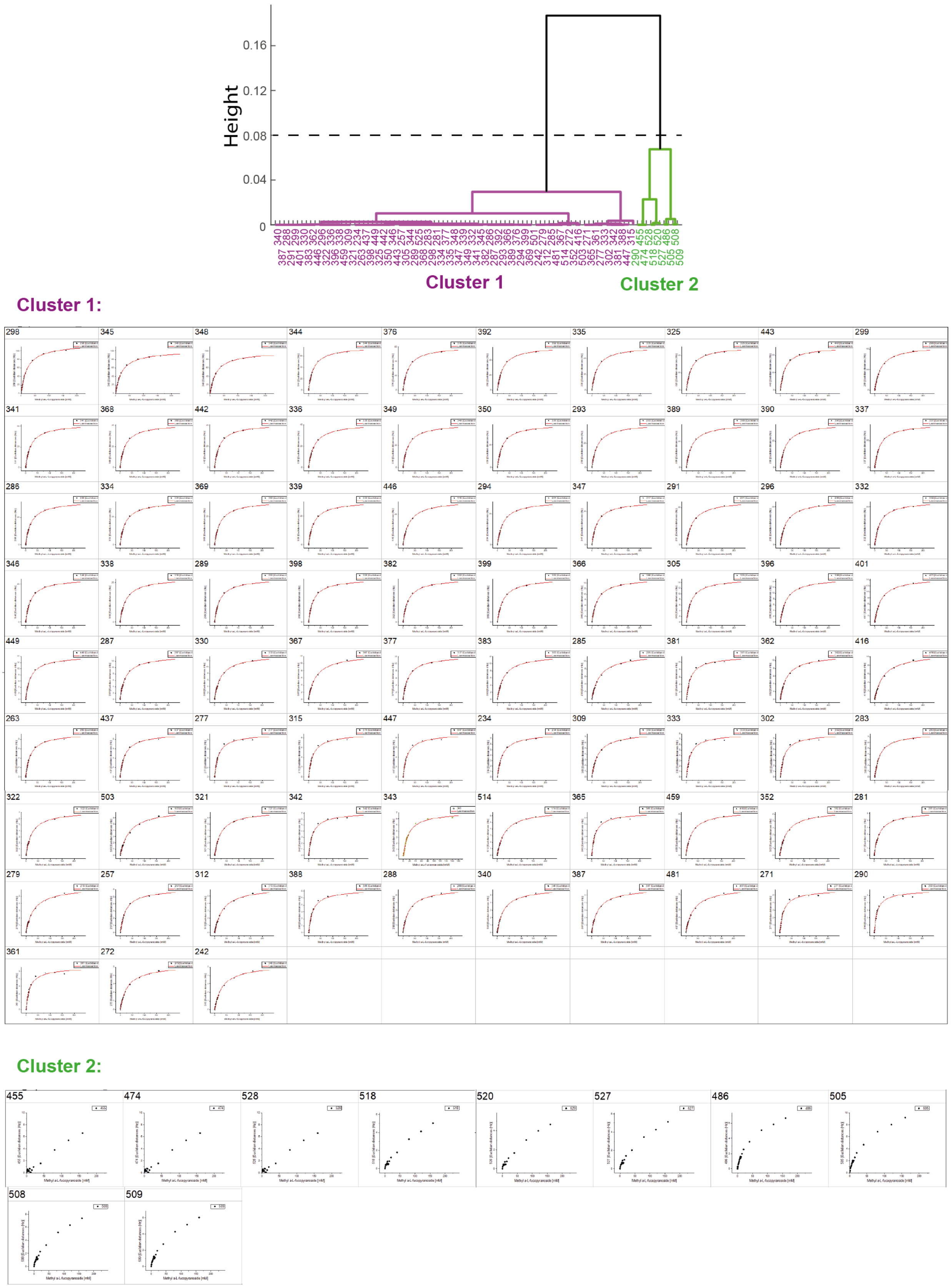

Supplementary Figure 13: Shape of binding isotherms for clusters 1 and 2 identified by covariance analysis and hierarchical clustering.

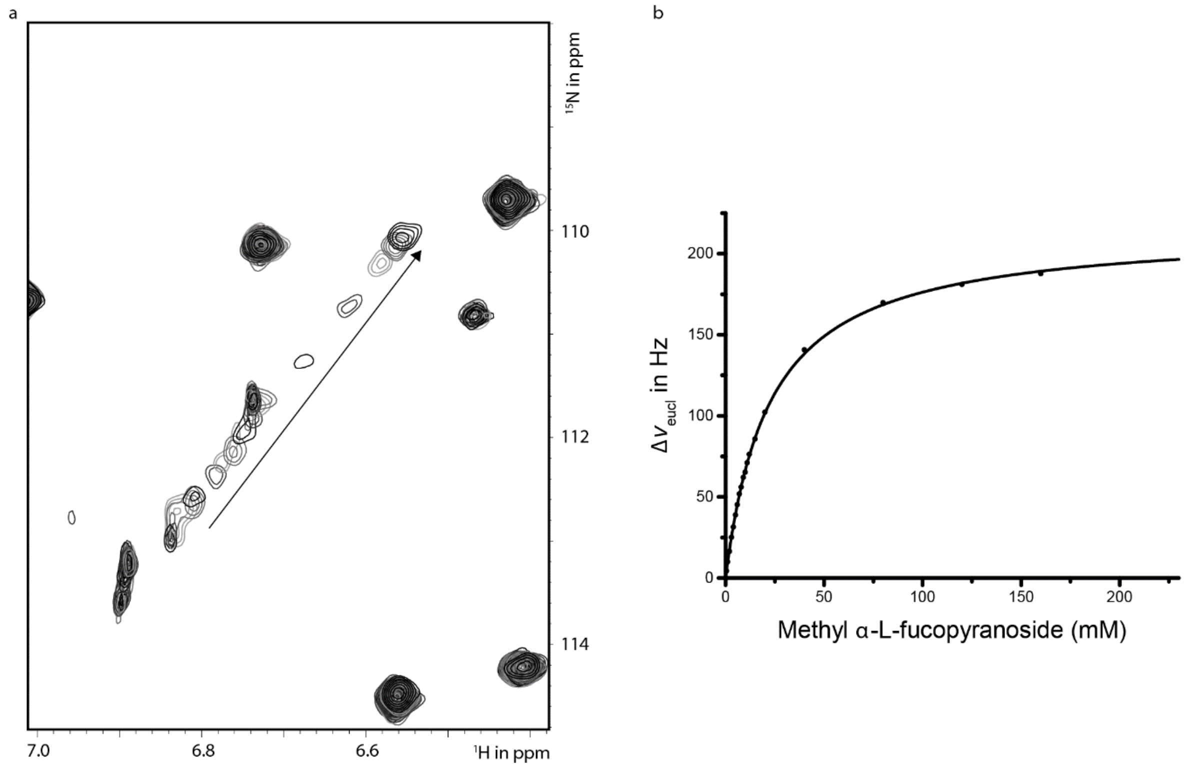

Supplementary Figure 14: a) Overlay of ^{1}H,^{15}N TROSY HSQC spectra of *GII.4* Saga NN P-dimers upon titration with methyl α-L-fucopyranoside showing chemical shift perturbations of the tentatively assigned 373N side chain signal. **b)** Fitting of the corresponding binding isotherm yields a K_D of 22 mM which is in perfect agreement with the results from global fitting (Fig. 4 of the main text and Supplementary Table 8).

2.3.10 Reevaluation of previously published titration data of methyl α-L-fucopyranoside binding to [U-^{2}H,^{15}N] Saga P-dimers

With the assignment of the backbone NH signals for wildtype and deamidated species in hand we could conduct a more specific SVD analysis. First, the assignment had to be transferred to the old dataset. In our current study a buffer optimized for the acquisition of triple resonance 3D NMR spectra (20 mM sodium phosphate pH 7.30) was used whereas the old study employed a Tris buffer (25 mM Tris pH 7.30 and 300 mM NaCl). Comparison of the corresponding ^{1}H,^{15}N TROSY HSQC spectra allowed unambiguous assignment of 187 out of 210 NH signals. Of note, in the old sample the protein was not refolded, meaning that around 30% of the signals are not visible in these spectra. Finally, only NH backbone signals corresponding to the non-deamidated species were subjected to SVD for noise reduction as described above (cf. 3.3.7).

The new SVD analysis afforded three non-noise components, as shown from the autocorrelation factors for $\mathbf{v}_i$. Compared to our previous analysis[18] it is immediately apparent that components 1, 2, and 3 conserve a similar shape (Supplementary Figure 15, shape of $\mathbf{v}_i$ vectors), strongly suggesting that they capture the same events. However, $\mathbf{v}_4$ dramatically alters its smooth shape acquiring a jagged aspect, indicating random noise (as can be seen from the low autocorrelation observed for $\mathbf{v}_4$ in Supplementary Figure 15). Taking all together, these results suggest that the 4th component obtained in our first SVD analysis was associated to the small percentage of deamidated protein formed over time during the titration.

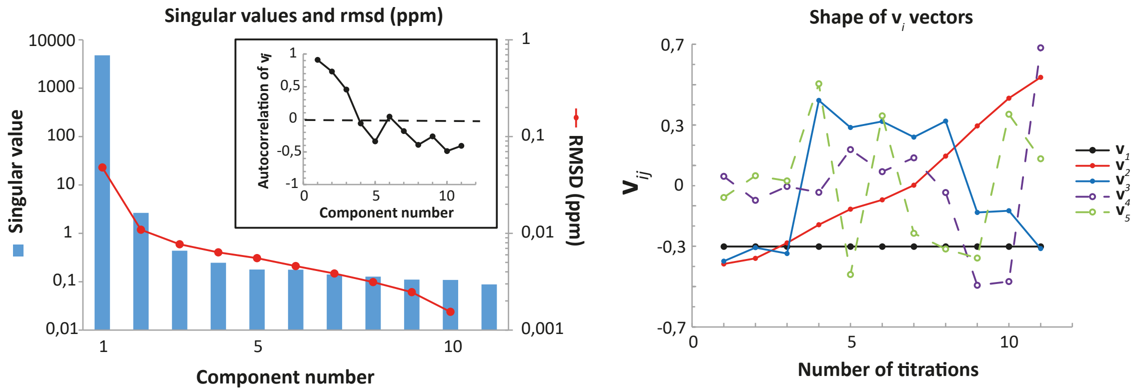

Supplementary Figure 15: SVD analysis for peaks corresponding to NH backbone signals of the Saga P-dimers from the wildtype species. Size and autocorrelation of individual components (left). Shape of component vectors (right).

Data were denoised by reconstructing the spectra from the first 3 SVD components, which represents 99.97% of the raw data and introduces an error below 0.008 ppm in the reconstructed data. CSPs were calculated from the denoised dataset according to Supplementary Equation (6). Only amino acids showing CSPs larger than 2σ at the highest ligand concentration were selected for data analysis. This left titration curves for 12 amino acids, which were subjected to non-linear least squares fitting of Supplementary Equation (11), furnishing a dissociation constant K_D of 16.2 ± 0.3 mM (R^2 = 0.997, χ^2 = 1.37, Supplementary Figure 16).

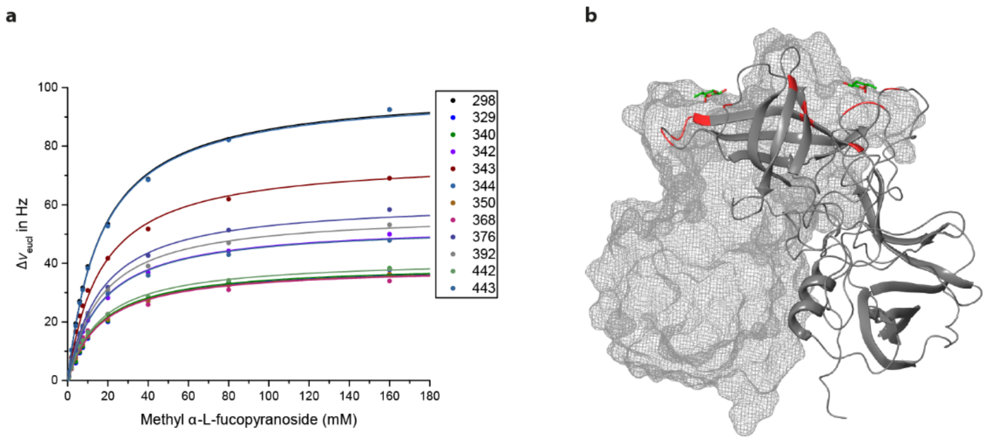

Supplementary Figure 16: Binding isotherms and location of amino acids exhibiting CSPs larger than 2σ. Data were from a previously published dataset for the titration of methyl α-L-fucopyranoside to a sample containing NN P-dimers with an unknown percentage of deamidated P-dimers (see main text for details).

3 Supplementary note 3: Sequence and structural alignment of P-domains of GII.4 norovirus strains

To collect the NoV VP1 sequences for the structural alignment we ran a PSI-BLAST search against the Saga VP1 amino acid sequence ([Norovirus Hu/GII-4/Saga1/2006/JP], GenBank accession number BAG70515.1) in the NCBI database for non-redundant protein sequences (August 2018).

(https://blast.ncbi.nlm.nih.gov/Blast.cgi?CMD=Web&PAGE=Proteins&PROGRAM=blastp&RUN_PSIBLAST=on). The search was restricted to the Norovirus group (taxid: 142786) and to a protein length between 500 to 600 amino acids, using a PSI-BLAST threshold of 0.001. Sequences containing more than 20% ambiguous amino acids were removed. Remaining sequences (3020) were structurally aligned using the online PROMALS3D server[19] (http://prodata.swmed.edu/promals3d/promals3d.php). Aligned sequences were classified according to their headers into the different genogroups and genotypes using an in-house Matlab R2016b script. Finally, genogroup *GII.4* (1746 sequences) was inspected at positions 372, 373 and 374 (numbering referenced to Saga1/2006).

GII.4 hNoV	
**Position**	**Most frequent amino acids**
372	D 42.9%, E 25.5%, N 17.4%, S 6.8%
373	**N 66.8%**, H 24.7%, D 4.6%, R 2.5%
374	D 99.8%

Supplementary Table 9: Amino acid abundance at positions 372, 373 and 374 (referenced to Saga1/2006) observed for *GII.4* hNoV from a structural alignment of 1746 sequences.

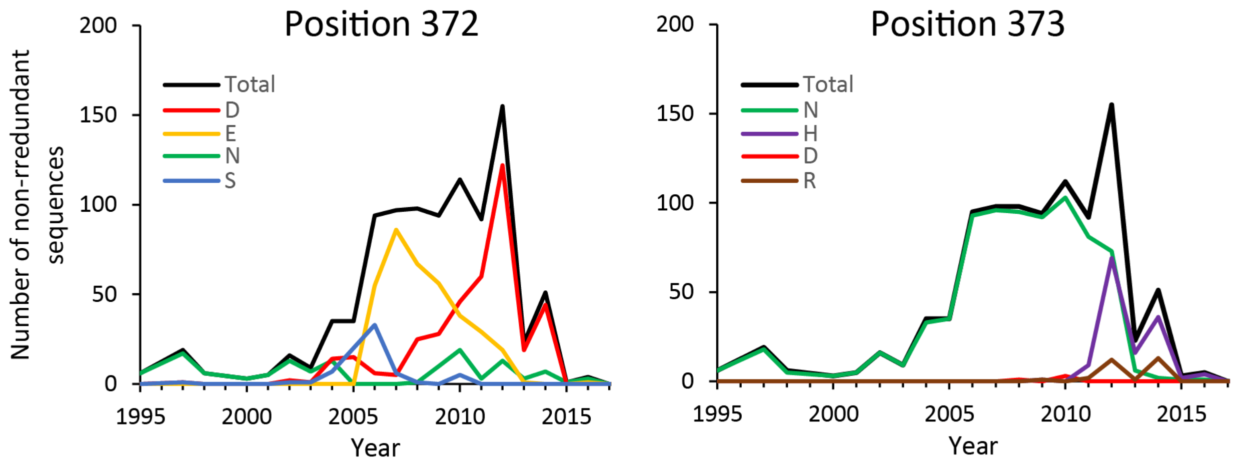

Supplementary Figure 17: Diagrams showing the evolution of positions 372 and 373 in hNoV *GII.4* (referenced to Saga1/2006) based on non-redundant sequences obtained from the NCBI database for proteins. Only sequences displaying in the header the year of collection were used to construct the graphics (978 sequences).

3.1 *Screening the Protein Data Bank for N373 in the reactive conformation*

The first step in the deamidation of Asn and isomerization of Asp under physiological conditions is the formation of a short-lived tetrahedral intermediate, which quickly transforms into a semi-stable succinimide intermediate, to finally hydrolyze into iso-Asp and/or Asp. The formation of the tetrahedral intermediate is believed to be the rate-limiting step at near-neutral pH. This tetrahedral intermediate is formed through the attack of the backbone nitrogen from the i+1 residue to the C^{γ} atom of the Asn or Asp, and is favored when the side chain adopts conformations that minimize the nucleophilic attack distance (d_{NuA}). The side-chain and backbone dihedral angles χ and ψ, corresponding to C_i'-C_i^{α}-C_i^{β}-C_i^{γ} and N_i-C_i^{α}-C_i'-N_{i+1}, have the highest impact on d_{NuA}. Two reactive conformations have been described corresponding to combinations of χ and ψ of either χ ~ 60° and ψ ~ 180°, or χ ~ -60° and ψ ~ 0°. [20]

An in-house Matlab R2016b script was used to screen the complete PDB for crystal structures of Norovirus P-dimers containing Asn amino acids in reactive conformations located in the flexible loop adjoining the HBGA binding site (positions 372 and 373 in Saga1/2006 P-dimers). To account for amino acid inclusions or deletions between different genogroups, we used the structural alignment described above to identify the equivalent positions through the different sequences. Of 108 crystal structures available in the PDB (August 2018), 19 crystal structures belonging to six different *GII.4* strains showed enough electron density at positions 372 and 373 to determine the side chain conformation of N373 (Supplementary Figure 18, Supplementary Table 10).

We also compared the corresponding loop conformations of the four strains, for which we studied deamidation using IEX (see 5.2 below) and MS (see 7.1 below). The structures of P-dimers of *GII.4* Saga (pdb 4x06, green), *GII.10* Vietnam 026 (pdb 3q38, blue), and *GII.17* Kawasaki 308 (pdb 5f40, orange) have been superimposed (Supplementary Figure 19), showing very similar backbone conformations in the loop region comprising positions 370 to 376. We propose that the formation of this loop is an important prerequisite for deamidation.

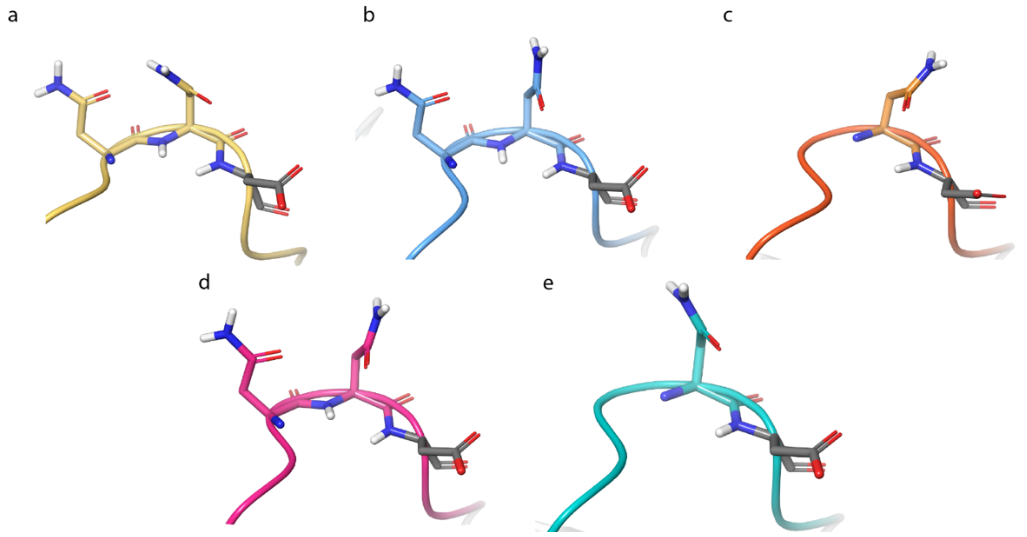

Supplementary Figure 18: Representative crystal structures from each *GII.4* NoV strain containing Asn residues at position 372 and/or 373 in a reactive conformation. **a)** VA387 (yellow, PDB code 2obt), **b)** CHDC2094 1974 (blue, PDB code 5iyq), **c)** NL 2004 (orange, PDB code 3sld), **d)** Farmington Hills 2004 (pink, PDB code 4oov), and **e)** Saga 2006 (turquoise, PDB code 4wzl). Asp 374 is always depicted in grey.

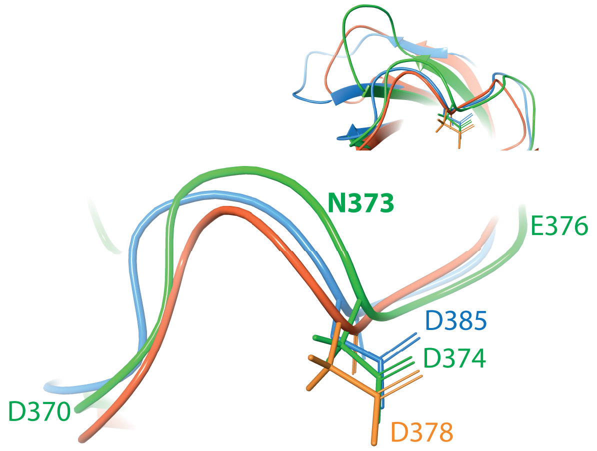

Supplementary Figure 19: Superimposition of crystal structures of *GII.4* Saga (pdb 4x06, green), *GII.10* Vietnam 026 (pdb 3q38, blue), and *GII.17* Kawasaki 308 (pdb 5f40, orange) P-dimers showing the loop region (D370-E376) containing D374 and N373 in Saga P-dimers. The amino acid in the position equivalent to N373 in Saga P-dimers is a glutamate (E384) in Vietnam 026, and an aspartate (D377) in Kawasaki 308 P-dimers. The backbone conformations of this loop are rather similar for the different genotypes *GII.4*, *GII.10*, and *GII.17*, as this is also observed when comparing different strains of one genotype, *GII.4* (Supplementary Fig. 18). Based on our deamidation studies with *GII.4* Saga, *GII.4* MI001 (no crystal structure available yet), *GII.10* Vietnam 026, and *GII.17* Kawasaki 308 (see Supplementary Figs. 20-22) and on the analysis of available crystal structure data it seems that the loop conformation is conserved whether or not asparagine is present. We suggest that the conformation of the loop itself is crucial and deamidation of an asparagine residue preceding the highly conserved aspartate residue (D378, D374, D385) can be expected to be a common phenomenon.

Identifier	PDB code/resolution (Å)	i, i+1	Chain	χ°	ψ°	d_{NuA} (Å)
***GII.4* VA387**	**2obt/2.00**	**$N^{372}N$**	**A**	**-62**	**18**	**3.866**
		**$N^{373}D$**	**A**	**-67**	**-5**	**3.543**
***GII.4* CHDC2094 1974**	**5iyq/1.41**	**$N^{372}N$**	**A**	**-63**	**19**	**3.974**
		**$N^{373}D$**	**A**	**-60/-58**	**-13/-11**	**3.435/3.586**
		$N^{372}N$	B	-62	12	3.892
		$N^{373}D$	B	-51	1	3.623
	5iyn/1.56	$N^{372}N$	B	-63	11	3.848
		$N^{373}D$	B	-43	15	3.731
	5iyp/1.27	$N^{372}N$	A	-64	17	3.980
		$N^{373}D$	A	-63	-6	3.656
		$N^{372}N$	B	-61	11	3.853
		$N^{373}D$	B	-60	1	3.732
	5iyr/1.54	$N^{373}D$	A	-65	-9	3.474
		$N^{372}N$	B	-60	14	3.885
	5iyw/1.41	$N^{372}N$	A	-63	18	3.988
		$N^{373}D$	A	-63/-65	-13/-4	3.478/3.693
		$N^{373}D$	B	-63	0	3.756
***GII.4* NL 2004**	**3sld/2.68**	**$N^{373}D$**	**J**	**-50**	**-7**	**3.239**
		$N^{373}D$	C	-71	0	3.822
		$N^{373}D$	E	-45	2	3.605
	3sej/3.04	$N^{373}D$	B	-76	0	3.734
	3skb/3.22	$N^{373}D$	B	-69	9	3.799
		$N^{373}D$	F	-71	-15	3.413
	3sln/2.84	$N^{373}D$	C	-70	-9	3.683
***GII.4* Farmington Hills 2004**	**4oov/1.53**	**$N^{372}N$**	**A**	**-62**	**8**	**3.813**
		**$N^{373}D$**	**A**	**-57**	**1**	**3.640**
		$N^{373}D$	B	-59	0	3.566
	4ops/1.76	$N^{372}N$	A	-60	14	3.858
		$N^{373}D$	A	-63	-11	3.448
		$N^{372}N$	B	-60	17	3.891
		$N^{373}D$	B	-61	-16	3.341
	4opv/1.85	$N^{372}N$	A	-63	6	3.763
		$N^{373}D$	A	-59	1	3.695
		$N^{373}D$	B	-57	-2	3.529
	4x05/1.98	$N^{372}N$	A	-76	6	3.832
		$N^{373}D$	A	-65	-5	3.587
		$N^{372}N$	B	-69	11	3.884
		$N^{373}D$	B	-67	-6	3.573
		$N^{372}N$	C	-79	5	3.913
		$N^{373}D$	C	-62	-1	3.636
		$N^{372}N$	D	-68	9	3.846
		$N^{373}D$	D	-66	-6	3.599
***GII.4* Saga 2006**	**4wzl/1.70**	**$N^{373}D$**	**A**	**-79**	**-7**	**3.700**
		$N^{373}D$	B	-78	-7	3.674
	4wzk/1.49	$N^{373}D$	A	-76	-3	3.721

	$N^{373}D$	B	-77	-3	3.728
4x06/1.22	$N^{373}D$	A	-78	-4	3.668
	$N^{373}D$	B	-78	-9	3.685
4x07/1.46	$N^{373}D$	A	-72	-1	3.769
	$N^{373}D$	B	-73	0	3.767
4oox/1.20	$N^{373}D$	A	-65	-8	3.506

[a]Values reported after the forward slash indicate secondary amino acid conformations in the reactive conformation. [b]Entries in bold are depicted in Supplementary Figure 18 as representative structures.

Supplementary Table 10: Values of conformational and chemical order parameters for *GII.4* NoV P-dimer crystal structures from the PDB containing Asn residues in the reactive conformation located in the flexible loop comprising amino acid residues 372 and 373 (adapted to the numbering of amino acids of the Saga1/2006 P-dimer structures).

4 Supplementary note 4: Deamidation of P-dimers

4.1 Rate of deamidation of GII.4 Saga P-dimers

We followed deamidation of *GII.4* Saga P-dimers over 48 h at 310 K using ion exchange chromatography (IEX). Using IEX the different forms, NN, iDN, and iDiD (cf. main text) can be separated (Supplementary Figure 20). From fitting an exponential decay curve to the decaying amounts of NN P-dimers their half-life is estimated as 1.6 d (Supplementary Figure 21).

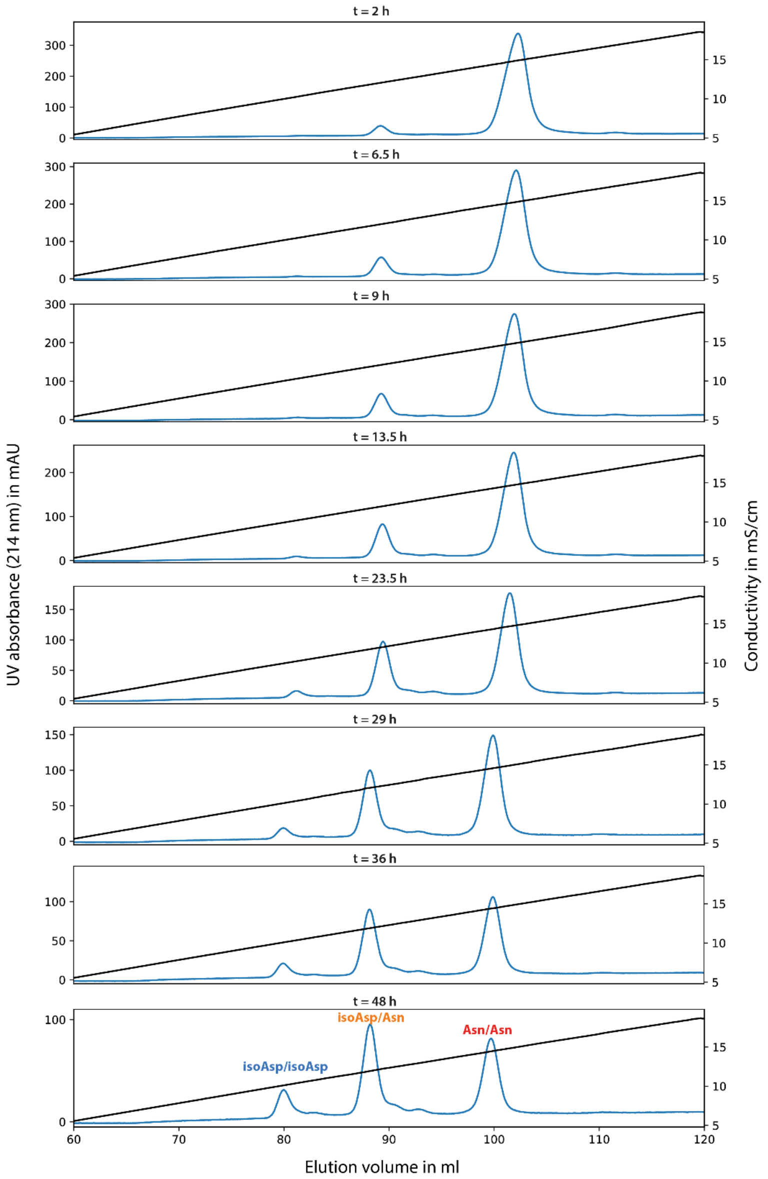

Supplementary Figure 20: Ion exchange chromatograms of eight P-dimer aliquots that were incubated up to 48 h at 310 K. UV absorbance has been measured at 214 nm (blue). Protein species elute with increasing conductivity (black). Three major fractions were identified, corresponding to fully (isoAsp/isoAsp, iDiD), asymmetrically (isoAsp/Asn, iDN) and non-deamidated protein species (Asn/Asn, NN), respectively. Note that the overall absorbance decreases due to some protein precipitation at prolonged incubation times. Precipitates were removed by centrifugation prior to analysis.

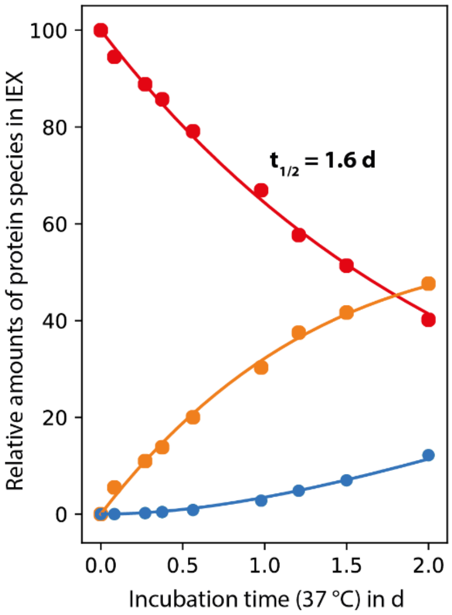

Supplementary Figure 21: Deamidation rate of P-dimers incubated in 75 mM sodium phosphate buffer, 100 mM NaCl, pH 7.3 for up to 48 h at 310 K. Relative amounts of the different protein species were estimated from their respective peak integrals in ion exchange chromatograms (Supplementary Figure 20): fully (iDiD, blue), asymmetrically (iDN, orange), and non-deamidated (NN, red) protein species. An exponential decay model was fitted to the decrease of non-deamidated protein yielding a half-life of 1.6 d. Note that this analysis could be biased due to protein precipitation during incubation.

4.2 Deamidation of P-dimers of strains GII.4 MI001, GII.10 Vietnam 026, and GII.17 Kawasaki 308

To test whether deamidation of N373 is also relevant for other strains than *GII.4* Saga we looked into deamidation of three other strains, *GII.4* MI001, *GII.10* Vietnam 026, and *GII.17* Kawasaki 308. For *GII.4* MI001 P-dimers no crystal structure is available yet but as the P-domains exhibit 92% sequence identity with *GII.4* Saga and as the critical loop region ($STDTSND^{374}$) is almost identical with the corresponding sequence in *GII.4* Saga ($STDTEND^{374}$) it is likely that deamidation occurs. The other two strains have no asparagine residue preceding the critical aspartate but the overall loop conformations are rather similar to the one in *GII.4* Saga (cf. Supplementary Figure 19). The sequences in that loop region are $STWETQD^{385}$ (*GII.10* Vietnam 026) and $RISDNDD^{378}$ (*GII.17* Kawasaki 308). For *GII.10* a glutamine precedes the critical aspartate. Deamidation of glutamine residues has also been described but should be slower. The observations match our predictions: *GII.4* MI001 deamidates whereas for the other two strains no deamidation was observed under the conditions chosen (Supplementary Figure 22).

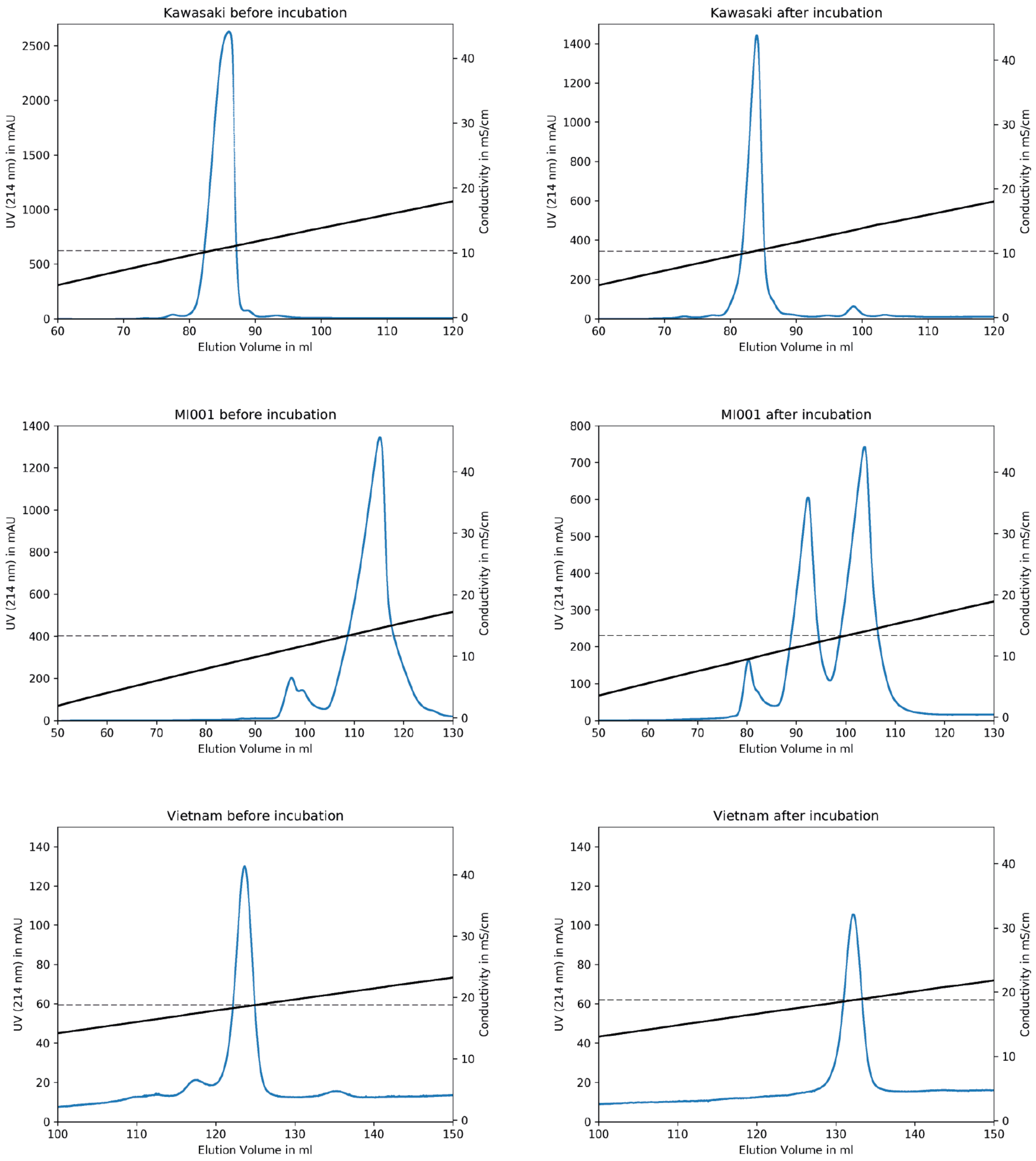

Supplementary Figure 22: Ion exchange chromatograms of *GII.17* Kawasaki 308, *GII.10* Vietnam O26 and *GII.4* MI001 P-dimers before and after incubation at elevated temperatures. UV absorbance has been measured at 214 nm (blue). The dashed line indicates the conductivity where the major peak elutes. Buffer batches changed over the course of the incubations and, therefore, elution volumes cannot be compared directly across the samples. The major peaks were separated and incubated at elevated temperatures to promote a potential deamidation reaction. Incubations conditions were as follows: *GII.4* MI001 and *GII.10* Vietnam O26 P-dimers were incubated in 25 mM Tris, 300 mM NaCl (pH 7.3) at 25 °C for 3 weeks. *GII.17* Kawasaki 308 P-dimers were incubated for 48 h at 37 °C in 25 mM Tris, 500 mM NaCl (pH 7.3). Protein samples taken after the incubation were tested for deamidation by MS (cf. Supplementary Table 12). In the case of *GII.4* MI001, protein samples from both the first (deamidated fraction in Supplementary Fig. 25) and the last peak (wildtype fraction in Supplementary Fig. 25) were tested.

5 Supplementary note 5: Crystallography

5.1 Ligand interaction diagram

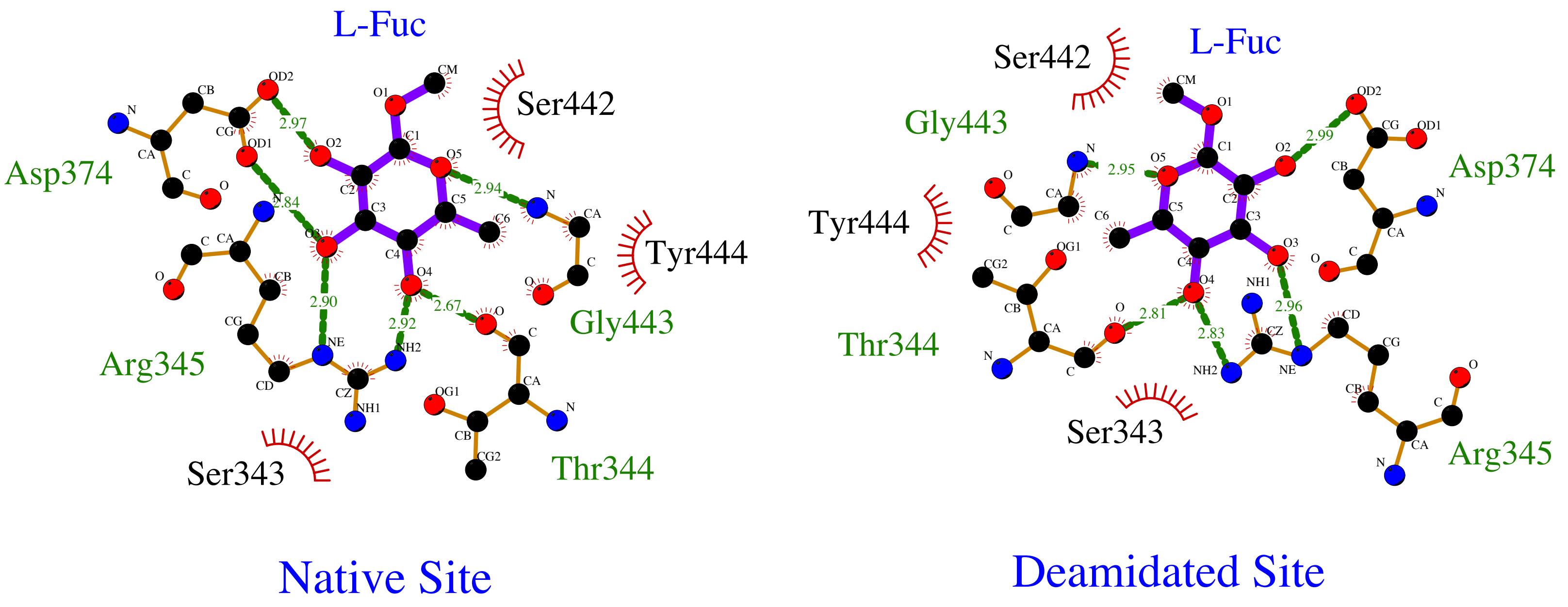

Supplementary Figure 23: Ligand interaction diagrams for methyl α-L-fucopyranoside bound to *GII.4* Saga P-dimers (pdb 6h9v) in the native and in the deamidated binding site. In the native site a bivalent hydrogen bond is formed between the side chain carboxyl group of Asp374 and the hydroxyl groups at positions C2 and C3 of methyl α-L-fucopyranoside. In the deamidated site, the Asp374 side chain carboxyl group makes only one hydrogen bond with the C2 hydroxyl group of methyl α-L-fucopyranoside. Plots were produced using the program LigPlot+ [21].

5.2 *Data collection and refinement statistics*

	**Deamidated Saga GII-4 P-dimer with methyl α-L-fucopyranoside**
Beamline	SLS X06SA
Wavelength [Å]	1.542
Resolution range* [Å]	39.58 - 1.52 (1.56 - 1.52)
Space group	P 1 2_1 1
Unit cell dimensions	a=54.29 Å, b=83.95 Å, c=65.13 Å<br>β=95.78°
Measured reflections	408121 (31035)
Unique reflections	88343 (6538)
Redundancy	4.62 (4.75)
Completeness [%]	99.0 (99.5)
I/σ(I)	11.18 (0.94)
CC1/2	0.999 (0.316)
Wilson B-factor [$Å^2$]	21.52
Reflections used in refinement	88339
Reflections used for R_{free}	4418
Number of non-hydrogen atoms	5656
macromolecules	5006
ligands	34
solvent	616
protein residues	614
Average B-factor [$Å^2$]	27.01
macromolecules	25.74
ligand (methyl fucose)	35.94
ligand ($Mg^{2+)}$	32.26
water	36.64
R_{work}	0.147 (0.305)
R_{free}	0.182 (0.349)
Rmsd bond length [Å]	0.008
Rmsd bond angles [°]	0.932
Ramachandran favored [%]	98.84
Ramachandran allowed [%]	1.16
Rotamer outliers [%]	0.36
Clashscore	2.33
Number of TLS groups	10

*Statistics for the highest-resolution shell are shown in parentheses.

Supplementary Table 11: Data collection and refinement statistics.

6 Supplementary note 6: Mass spectrometry

6.1 Identification of deamidation sites

a

Sequence	Peptide position	Charge	Theoretical m/z (wt)	Observed m/z	Deamidation position probability for N373	Deamidated fraction in %
STDTENDFETHQ	368-379	2	712.284	712.7761	1	100
STDTENDFETHQNTKFTPVG	368-387	3	756.6732	757.0012	0.708	96.6
STDTENDFETHQNTKFTPVG	368-387	2	1134.506	1134.998	0.998	98.1

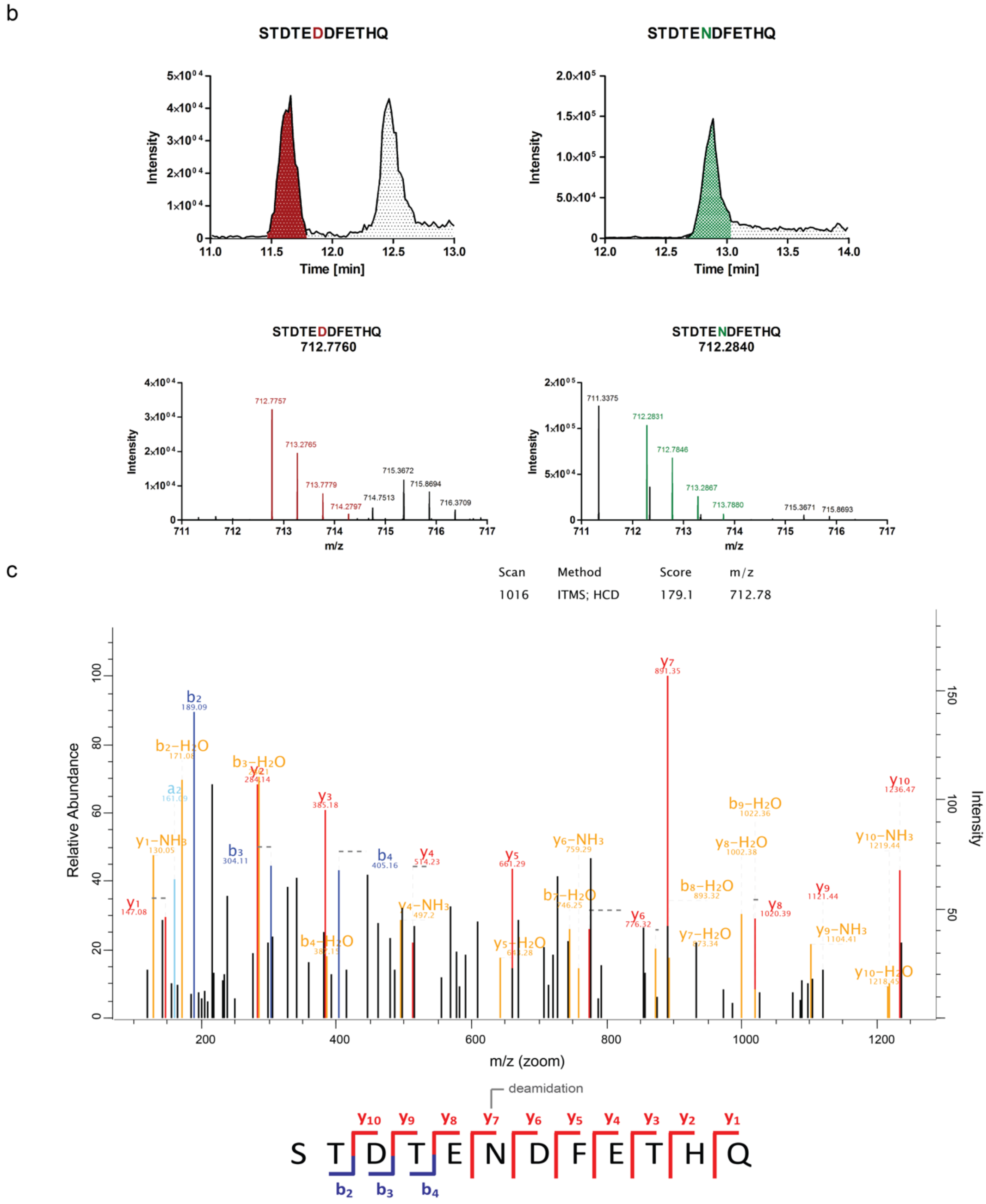

Supplementary Figure 24: a) Deamidated peptides identified by LC-MS/MS in the deamidated *GII.4* Saga P-dimer sample. Peptide identification and deamidation probability calculation for N373 was done using MaxQuant software. **b)** Chromatogram and spectrum of peptide STDTENDFETHQ (*z*=2) in both the deamidated (red) and wildtype (green) protein sample. The theoretical monoisotopic mass is indicated below the peptide sequence in the lower panel. The peptide shown here was omitted from the H/D analysis due to weak signal. **c)** Fragmentation spectrum of peptide STDTENDFETHQ in the deamidated sample. The fragment y7 (and all upstream y-fragments) shows a characteristic mass shift which clearly indicates the presence of the deamidation at N373.

Sample	Sequence	Peptide position	Charge	Theoretical *m/z* (wt)	Observed *m/z*	Deamidation site and probability	Fraction %
***GII.4* MI001 IEX-separated wildtype fraction**	STDTS**N**DFETGQNTRFTPVG	368-387	2	1087.4852	1087.4844	-	100
	FRSTMPGCSGYP**N**MNL	434-449	2	887.8891	887.8887	-	96
					888.3805	N446 (0.876)	4
	FFRSTMPGCSGYP**N**MNL	433-449	2	961.4233	961.4228	-	96
					961.9153	N446 (0.5)	4
***GII.4* MI001 IEX-separated deamidated fraction**	STDTS**N**DFETGQNTRFTPVG	368-387	2	1087.4852	1087.9762	N373 (0.971)	100
	FRSTMPGCSGYPNM**N**L	434-449	2	887.8891	887.8887	-	86
					888.3811	N448 (0.546)	14
	FFRSTMPGCSGYPNM**N**L	433-449	2	961.4233	961.4228	-	83
					961.9147	N448 (0.58)	17
	RSTMPGCSGYP**N**MNL	435-449	2	814.3549	814.3545	-	86
					814.8469	N446 (0.688)	14
***GII.17* Kawasaki not incubated**	VNLRISDNDDF	369-379	2	654.3149	654.3139	-	100
	LRISDNDDF	371-379	2	547.7593	547.7589	-	100
	LRISDNDDFQ	371-380	2	611.7885	611.7877	-	100
***GII.17* Kawasaki incubated**	VNLRISDNDDF	369-379	2	654.3149	654.3144	-	100
	LRISDNDDF	371-379	2	547.7593	547.7593	-	100
***GII.10* Vietnam incubated**	STWETQDVSSGQPTKFTPVG	379-398	2	1076.5133	1076.5128	-	100
	STWETQDVSSGQPTKFTPVGL	379-399	3	755.706	755.7054	-	100
	STWETQDVSSGQPTKFTPVGLA	379-400	2	1168.5739	1168.5732	-	100
	STWETQDVSSGQPTKFTPVGLAS	379-401	2	1212.0899	1212.0891	-	100

Supplementary Table 12: Deamidated peptides identified by LC-MS/MS for different strains. Samples originate from experiments in Supplementary Figure 22. Peptide identification and deamidation probability calculation was done using MaxQuant software. In addition to N373 deamidation, a second deamidation site was identified in *GII.4* MI001 at either N446 or N448. The asparagine with the highest deamidation probability for the specific peptide is marked in bold.

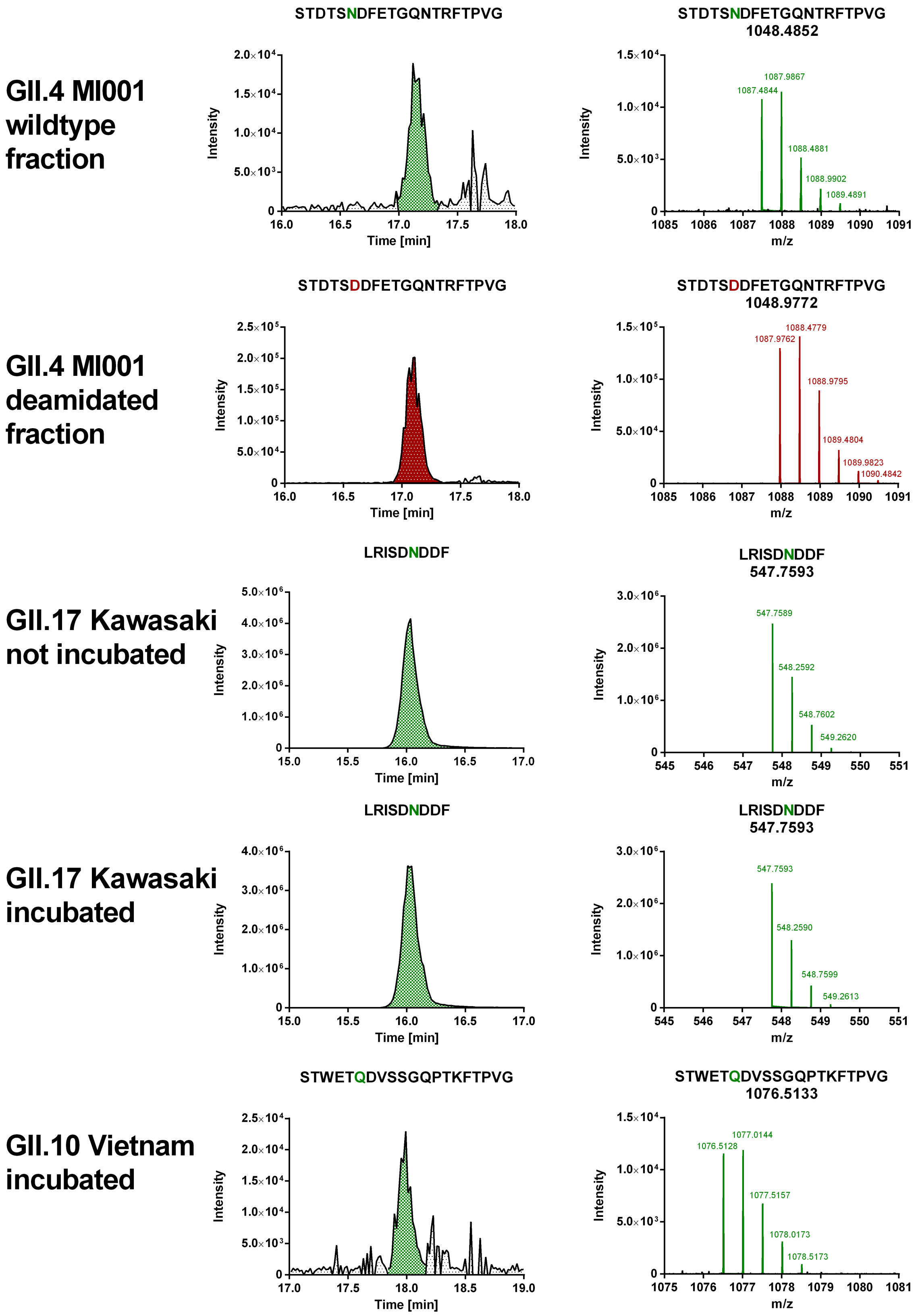

Supplementary Figure 25: Chromatograms and mass spectra of peptides (z=2) covering the potentially deamidated loops in *GII.4* MI001, *GII.17* Kawasaki and *GII.10* Vietnam P dimers (see also Supplementary Table 12 and Supplementary Figure 22). The theoretical monoisotopic mass is indicated below the peptide sequence in the mass spectrum.

6.2 *P-domain peptide coverage*

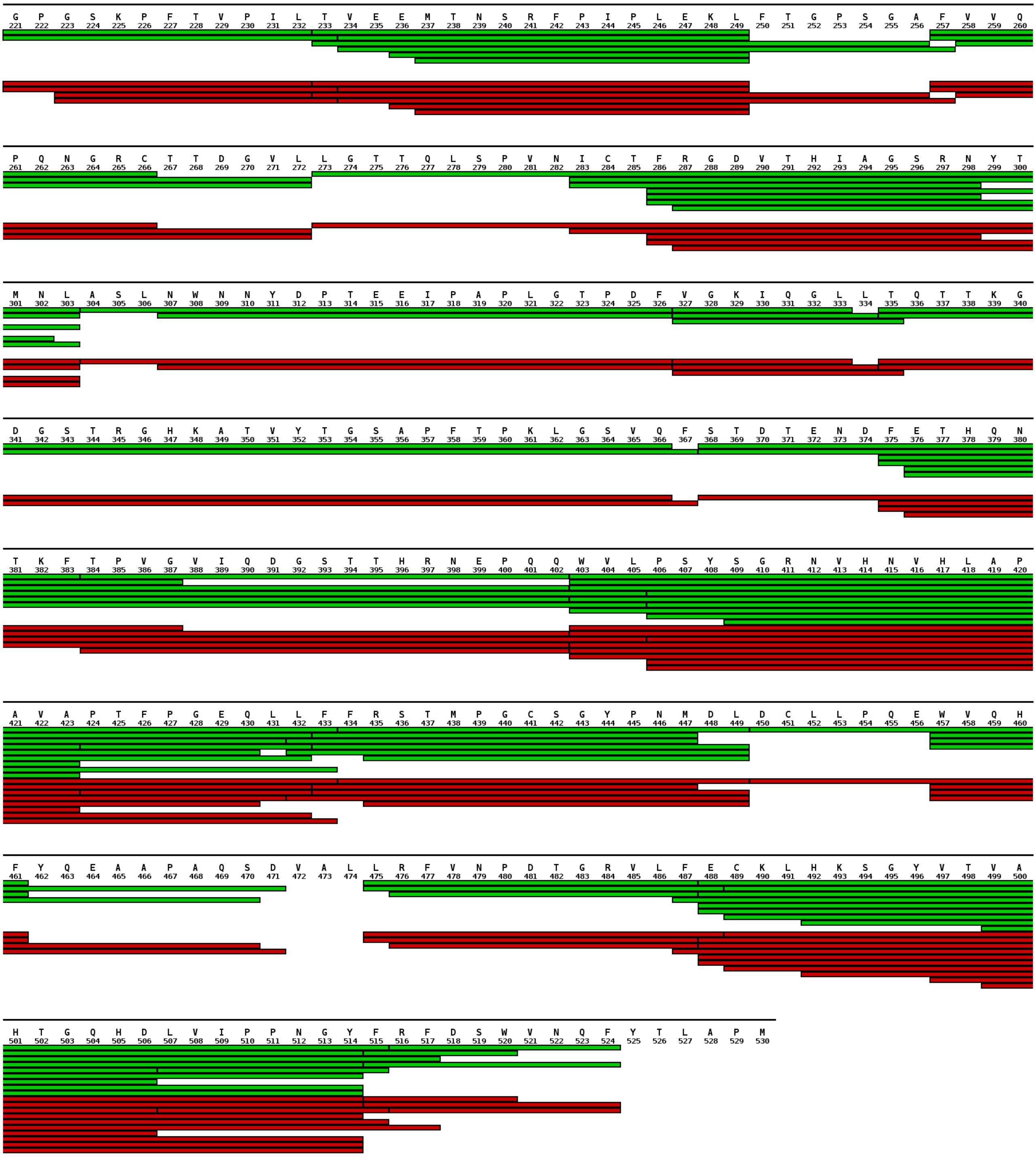

Supplementary Figure 26: *GII.4* Saga P-domain peptide coverage map for the wildtype (green) and deamidated protein (red). Peptides for the two missing regions 472-474 and 525-530 were identified in the non-deuterated sample, but data quality in the deuterated samples was insufficient to be included in the HDX MS analysis.

6.3 *HDX data summary tables*

**Dataset 1**	**WILDTYPE (unbound)**	**10 mM B trisaccharide**
HDX reaction details	20 mM Tris buffer, pH= 7.4, 150 mM NaCl, 25°C	20 mM Tris buffer, pH= 7.4, 150 mM NaCl, 25°C
HDX time course (min)	1, 10, 60, 480	1, 10, 60, 480
HDX control samples	-	-
Back-exchange (mean / IQR)	mean 37%	mean 37%
# of Peptides	68	68
Sequence coverage	97%	97%
Average peptide length / Redundancy	18.8 / 4.12	18.8 / 4.12
Replicates (biological or technical)	3 (technical)	3 (technical)
Repeatability	0.083 (average standard deviation)	0.111 (average standard deviation)
Significant differences in HDX (delta HDX > X D)	T-test with α=0.01 was used to compare significant differences between both states at a given time point	

**Dataset 2**	**DEAMIDATED (unbound)**	**10 mM B trisaccharide**
HDX reaction details	20 mM Tris buffer, pH= 7.4, 150 mM NaCl, 25°C	
HDX time course (min)	1, 10, 60, 480	
HDX control samples	-	
Back-exchange (mean / IQR)	mean 37%	
# of Peptides	65	
Sequence coverage	97%	
Average peptide length / Redundancy	18.6 / 3.87	
Replicates (biological or technical)	3 (technical)	
Repeatability	0.126 (average standard deviation)	0.06 (average standard deviation)
Significant differences in HDX (delta HDX > X D)	T-test with α=0.01 was used to compare significant differences between both states at a given time point	

Supplementary Table 13: HDX data summary table according to the community-based recommendations. HDX data as well as all mass spectrometry raw data have been deposited to the ProteomeXchange Consortium via the PRIDE[22] partner repository with the dataset identifier PXD011914.

Dataset 3	WILDTYPE (unbound)	DEAMIDATED (unbound)
HDX reaction details	20 mM Tris buffer, pH= 7.4, 150 mM NaCl, 25°C	20 mM Tris buffer, pH= 7.4, 150 mM NaCl, 25°C
HDX time course (min)	1, 10, 60, 480	1, 10, 60, 480
HDX control samples	-	-
Back-exchange (mean / IQR)	mean 37%	mean 37%
# of Peptides	65	65
Sequence coverage	97%	97%
Average peptide length / Redundancy	18.6 / 3.87	18.6 / 3.87
Replicates (biological or technical)	3 (technical)	3 (technical)
Repeatability	0.081 (average standard deviation)	0.126 (average standard deviation)
Significant differences in HDX (delta HDX > X D)	combination of T-test with α=0.01 and delta HDX > 0.64 D (99% percentile calculated according to Arora et al.)	

Dataset 4	WILDTYPE (unbound)	10 mM B trisaccharide
HDX reaction details	20 mM Tris buffer, pH= 7.4, 150 mM NaCl, 25°C	20 mM Tris buffer, pH= 7.4, 150 mM NaCl, 25°C
HDX time course (min)	0.25, 1, 10, 60, 480	0.25, 1, 10, 60, 480
HDX control samples	-	-
Back-exchange (mean / IQR)	mean 37%	mean 37%
# of Peptides	72	72
Sequence coverage	99%	99%
Average peptide length / Redundancy	18.5 / 4.29	18.5 / 4.29
Replicates (biological or technical)	1 (technical)	1 (technical)
Repeatability	-	-
Significant differences in HDX (delta HDX > X D)	-	-
	**100 mM methyl-fucose**	**100 mM galactose**
HDX reaction details	20 mM Tris buffer, pH= 7.4, 150 mM NaCl, 25°C	20 mM Tris buffer, pH= 7.4, 150 mM NaCl, 25°C
HDX time course (min)	0.25, 1, 10, 60, 480	0.25, 1, 10, 60, 480
HDX control samples	-	-
Back-exchange (mean / IQR)	mean 37%	mean 37%
# of Peptides	72	72
Sequence coverage	99%	99%
Average peptide length / Redundancy	18.5 / 4.29	18.5 / 4.29
Replicates (biological or technical)	1 (technical)	1 (technical)
Repeatability	-	-
Significant differences in HDX (delta HDX > X D)	-	-

Supplementary Table 13: continued

Supplementary references:

1. Singh BK, Leuthold MM, Hansman GS. Human noroviruses' fondness for histo-blood group antigens. *J Virol* **89**, 2024-2040 (2015). DOI: 10.1128/JVI.02968-14

2. Dechavanne V*, et al.* A high-throughput protein refolding screen in 96-well format combined with design of experiments to optimize the refolding conditions. *Protein Expr Purif* **75**, 192-203 (2011). DOI:10.1016/j.pep.2010.09.008

3. Tan M, Jiang X. Norovirus gastroenteritis, carbohydrate receptors, and animal models. *PLoS Pathog* **6**, e1000983 (2010). DOI:10.1371/journal.ppat.1000983

4. Vranken WF, et al. The CCPN data model for NMR spectroscopy: development of a software pipeline. *Proteins* **59**, 687-696 (2005). DOI: 10.1002/prot.20449

5. Lee D, Hilty C, Wider G, Wuthrich K. Effective rotational correlation times of proteins from NMR relaxation interference. *J Magn Reson* **178**, 72-76 (2006). DOI: 10.1016/j.jmr.2005.08.014

6. Sklenar V, Bax A. Spin-Echo Water Suppression for the Generation of Pure-Phase Two-Dimensional NMR Spectroscopy. *J Magn Reson* **74**, 469-479 (1987). DOI: 10.1016/0022-2364(87)90269-1

7. Shi L, Kay LE. Tracing an allosteric pathway regulating the activity of the HslV protease. *Proc Natl Acad Sci U S A* **111**, 2140-2145 (2014). DOI: 10.1073/pnas.1318476111

8. Selvaratnam R, Chowdhury S, VanSchouwen B, Melacini G. Mapping allostery through the covariance analysis of NMR chemical shifts. *Proc Natl Acad Sci U S A* **108**, 6133-6138 (2011). DOI: 10.1073/pnas.1017311108

9. Aoto PC, Martin BT, Wright PE. NMR Characterization of Information Flow and Allosteric Communities in the MAP Kinase p38gamma. *Sci Rep* **6**, 28655 (2016). DOI: 10.1038/srep28655

10. Ruschak AM, Kay LE. Proteasome allostery as a population shift between interchanging conformers. *Proc Natl Acad Sci U S A* **109**, E3454-3462 (2012). DOI: 10.1073/pnas.1213640109

11. Ghasriani H*, et al.* Precision and robustness of 2D-NMR for structure assessment of filgrastim biosimilars. *Nat Biotechnol* **34**, 139-141 (2016). DOI: doi.org/10.1038/nbt.3474

12. Arbogast LW, Brinson RG, Marino JP. Mapping monoclonal antibody structure by 2D 13C NMR at natural abundance. *Anal Chem* **87**, 3556-3561 (2015). DOI: 10.1021/ac504804m

13. Kotz S, Nadarajah S. *Extreme value distributions. Theory and applications* (2000). DOI: 10.1142/p191

14. Schwarz G. Estimating the Dimension of a Model. *Ann Statist* **6**, 461-464 (1978). DOI: 10.1214/aos/1176344136

15. Baryshnikova OK, Williams TC, Sykes BD. Internal pH indicators for biomolecular NMR. *J Biomol NMR* **41**, 5-7 (2008). DOI: 10.1007/s10858-008-9234-6

16. Arai M, Ferreon JC, Wright PE. Quantitative analysis of multisite protein-ligand interactions by NMR: binding of intrinsically disordered p53 transactivation subdomains with the TAZ2 domain of CBP. *J Am Chem Soc* **134**, 3792-3803 (2012). DOI: 10.1021/ja209936u

17. Man PP, Bonhomme C, Babonneau F. Denoising NMR time-domain signal by singular-value decomposition accelerated by graphics processing units. *Solid State Nucl Magn Reson* **61-62**, 28-34 (2014). DOI: 10.1016/j.ssnmr.2014.05.001

18. Mallagaray A, Lockhauserbäumer J, Hansman GS, Uetrecht C, Peters T. Attachment of Norovirus to Histo Blood Group Antigens: A Cooperative Multistep Process. *Angew Chem Int Ed* **54**, 12014-12019 (2015). DOI:10.1002/anie.201505672

19. Pei J, Kim BH, Grishin NV. PROMALS3D: a tool for multiple protein sequence and structure alignments. *Nucleic Acids Res* **36**, 2295-2300 (2008). DOI: 10.1093/nar/gkn072

20. Plotnikov NV, Singh SK, Rouse JC, Kumar S. Quantifying the Risks of Asparagine Deamidation and Aspartate Isomerization in Biopharmaceuticals by Computing Reaction Free-Energy Surfaces. *J Phys Chem B* **121**, 719-730 (2017). DOI: 10.1021/acs.jpcb.6b11614

21. Laskowski RA, Swindells MB. LigPlot+: multiple ligand-protein interaction diagrams for drug discovery. *Journal of chemical information and modeling* **51**, 2778-2786 (2011). DOI: 10.1021/ci200227u

22. Vizcaino JA*, et al.* 2016 update of the PRIDE database and its related tools. *Nucleic Acids Res* **44**, D447-456 (2016). DOI: 10.1093/nar/gkv1145
