# Supplementary material for: A post-translational modification of human Norovirus capsid protein attenuates glycan binding
Source: Nat Commun. 2019 Mar 21;10:1320. doi: 10.1038/s41467-019-09251-5 (PMC6428809; doi:10.1038/s41467-019-09251-5)
Supplement: Supplementary file 3 — Description of Supplementary Files [file 41467_2019_9251_MOESM3_ESM.docx]

**Description of Additional Supplementary Files**

**File Name: Supplementary Data 1**

**Description:** Deuterium uptake plots for the wildtype P-dimers (grey) with 10 mM HBGA B Trisaccharide (blue). All time points (1 min, 10 min, 1 h, 8 h) were performed in triplicate. Error bars indicate the standard deviation (SD) of each triplicate analysis and time points with significant deuteration differences ΔD (p < 0.01) are marked with an asterisk.

**File Name: Supplementary Data 2**

**Description:** Deuterium uptake plots for the deamidated P-dimers (grey) with 10 mM HBGA B trisaccharide (blue). All time points (1 min, 10 min, 1 h, 8 h) were performed in triplicate. Error bars indicate the standard deviation (SD) of each triplicate analysis. No time point showed significant (p < 0.01) deuteration differences DD.

**File Name: Supplementary Data 3**

**Description:** Deuterium uptake plot comparison for the wildtype (grey) and deamidated P-dimers (red) without any ligand. All time points (1 min, 10 min, 1 h, 8 h) were performed in triplicate. Error bars indicate the standard deviation (SD) of each triplicate analysis and time points with significant deuteration differences DD (p < 0.01) are marked with an asterisk. Smaller deuteration differences can also be detected in the N-terminal domain loop (residues 221- 272). However, these are not considered significant according to the used significance definition.

**File Name: Supplementary Data 4**

**Description:** Deuterium uptake plots for the wildtype P-dimers with 10 mM HBGA B trisaccharide (blue), 100 mM methyl α-L-fucopyranoside (red) and 100 mM galactose (green, negative control). All time points (15 s, 1 min, 10 min, 1 h, 8 h) represent single measurements, but deuterium levels are well in line with the triplicate measurements presented in Supplementary Data 1.pdf.

**File Name: Supplementary Data 5**

**Description:** HDX MS data for non-deamidated and deamidated GII.4 Saga Pdimers in the absence and in the presence of blood group B trisaccharide, a-L-methyl fucopyranoside, and D-galactose.
