# Supplementary figures and images for: A post-translational modification of human Norovirus capsid protein attenuates glycan binding
Source: Nat Commun. 2019 Mar 21;10:1320. doi: 10.1038/s41467-019-09251-5 (PMC6428809; doi:10.1038/s41467-019-09251-5)

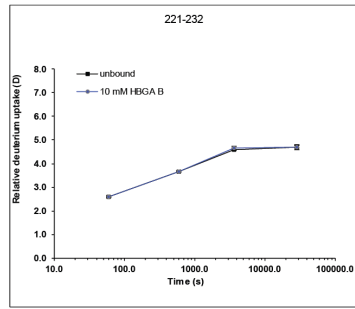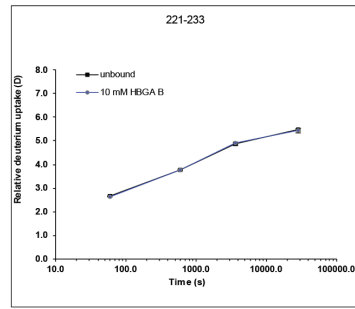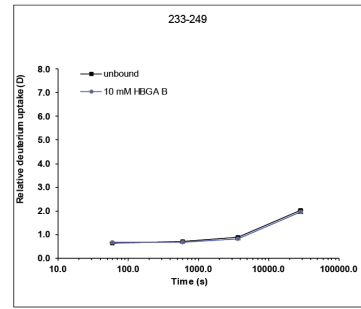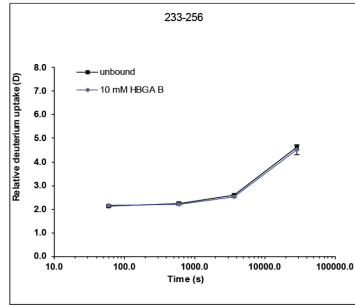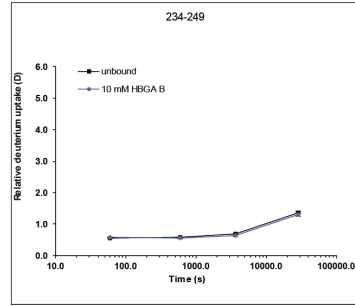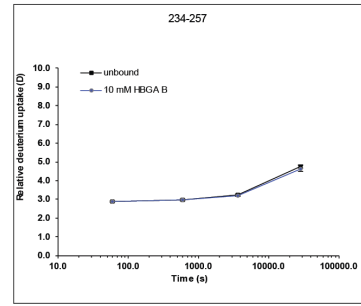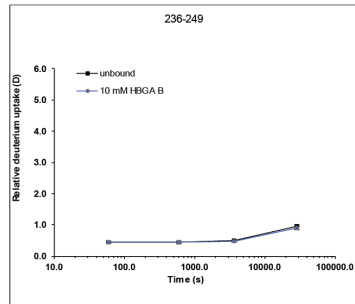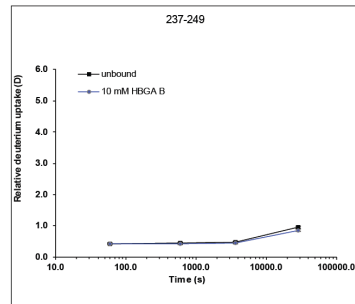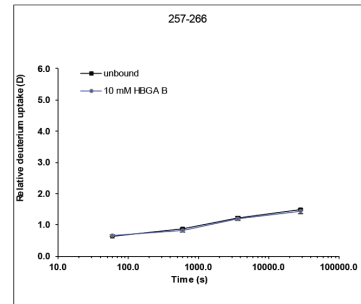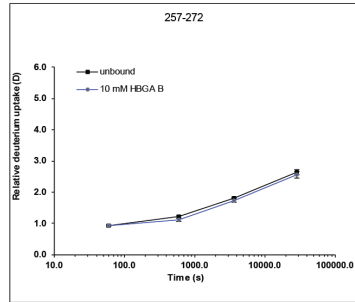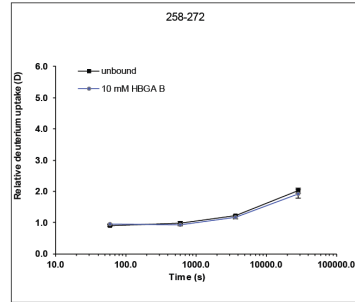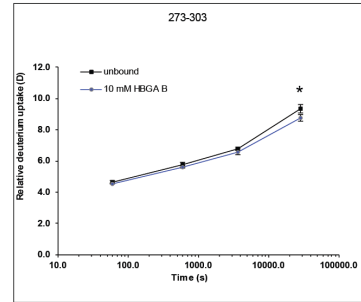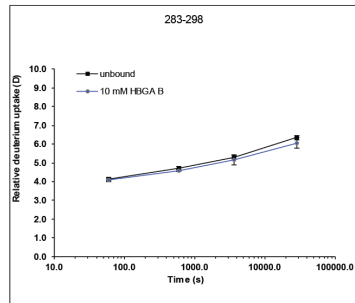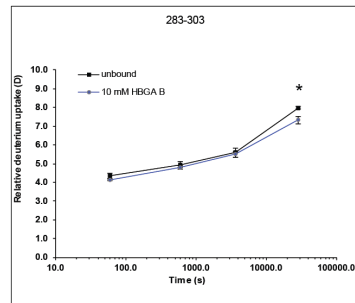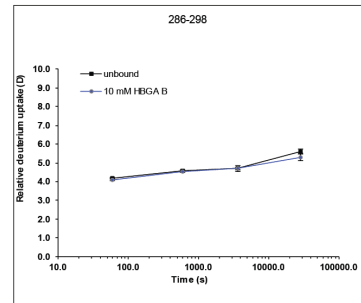

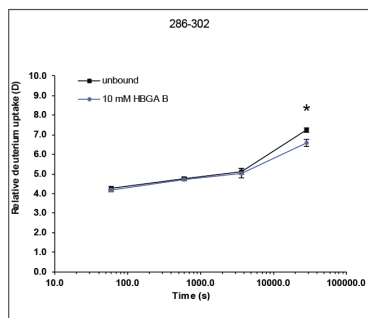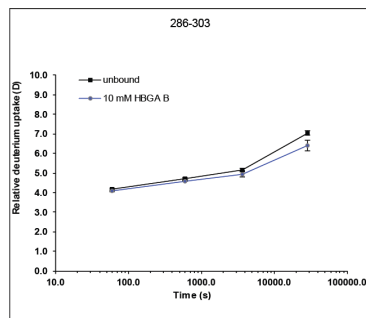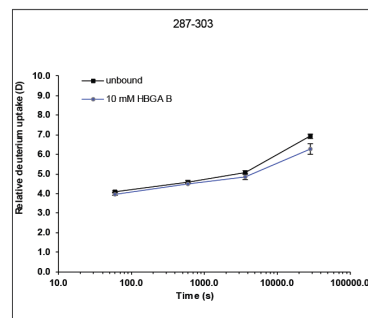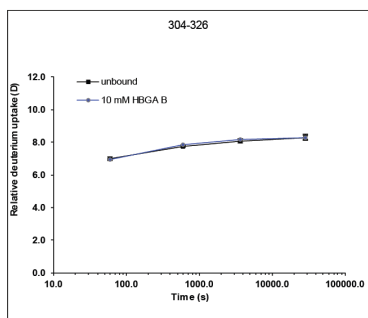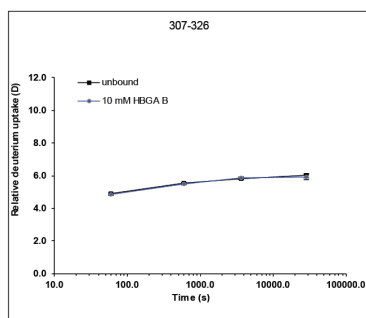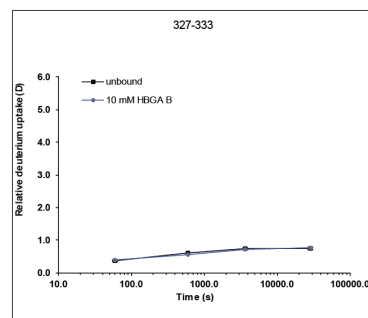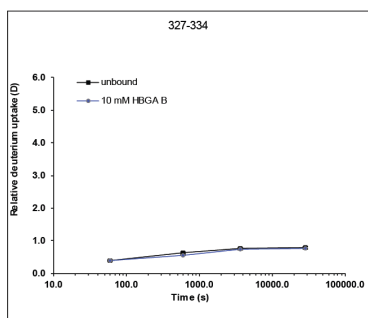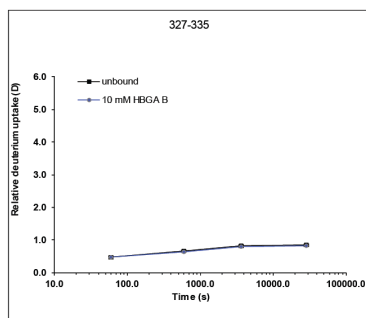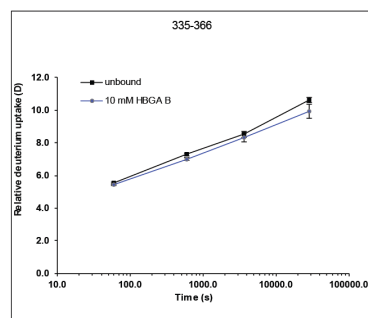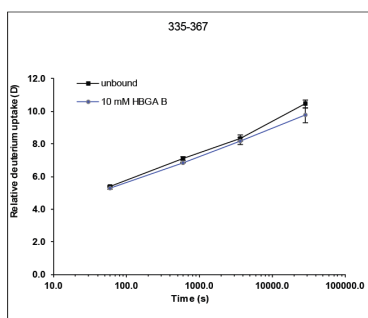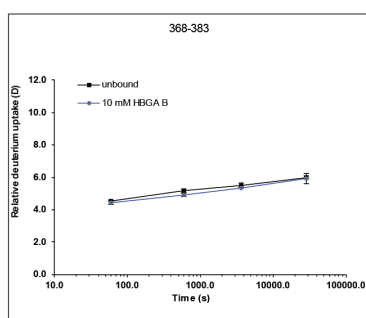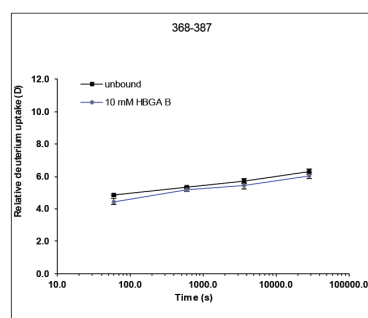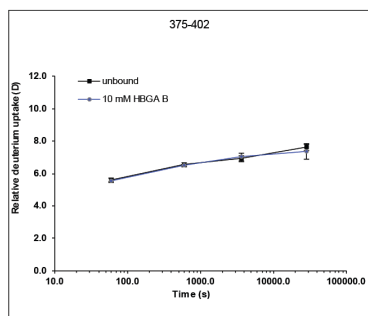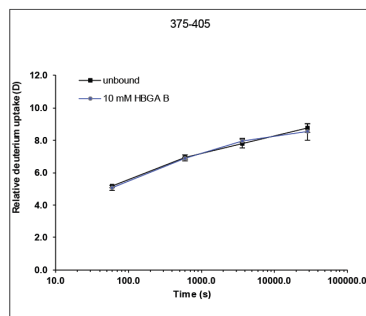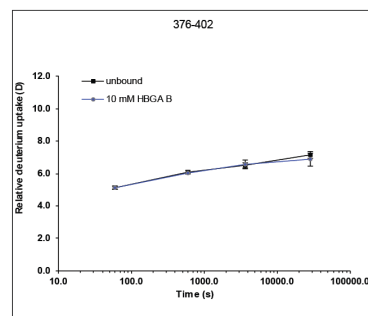

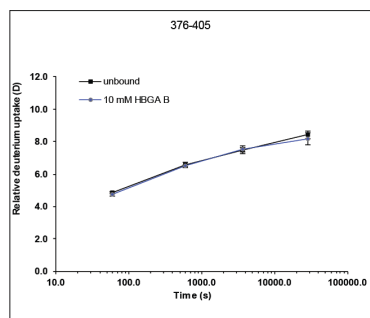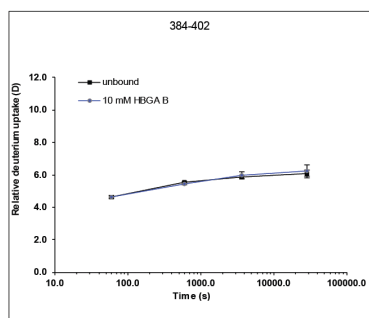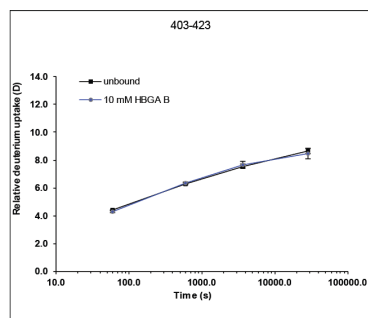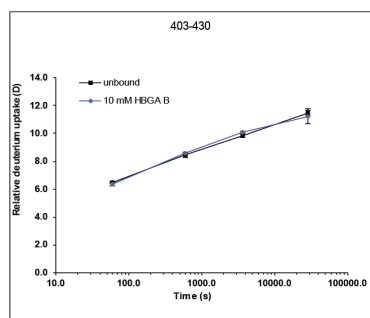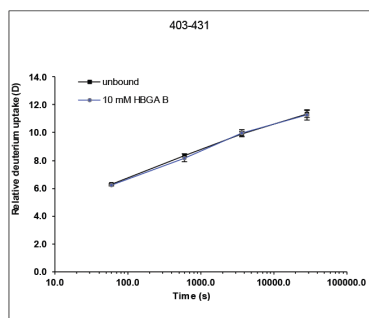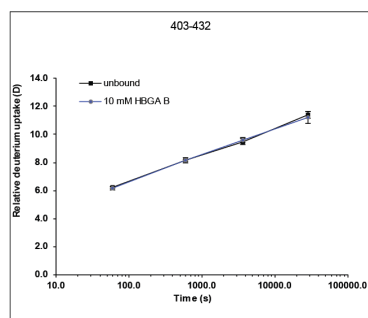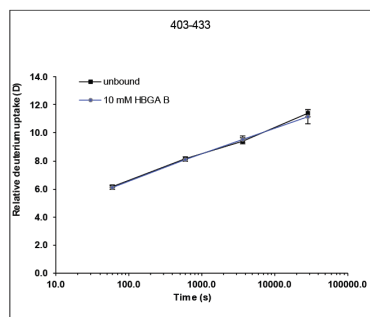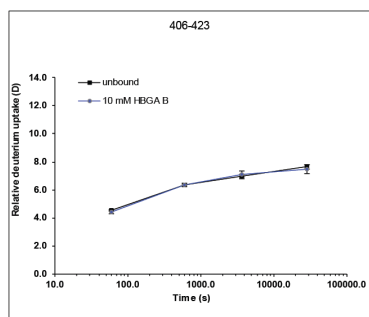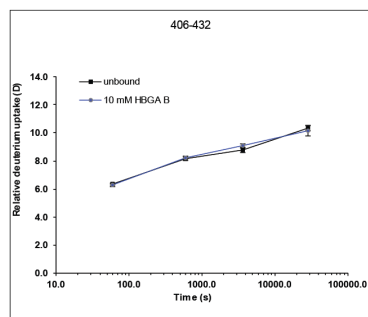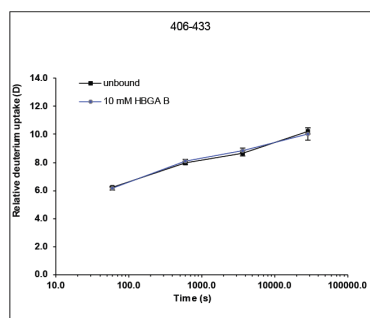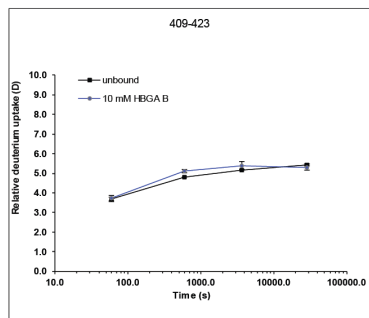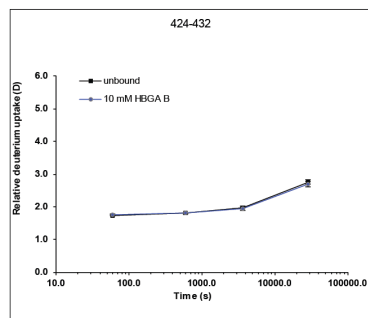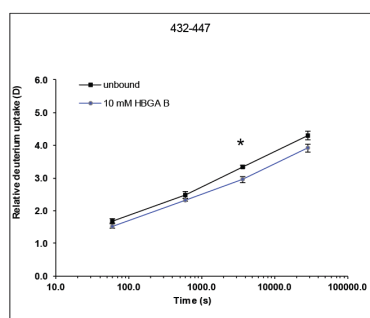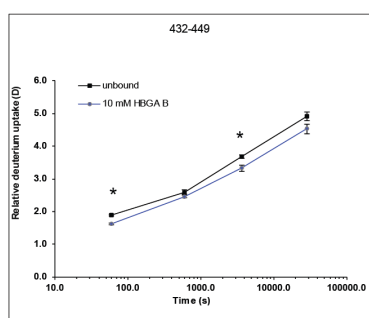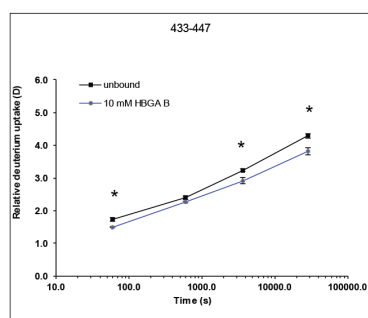

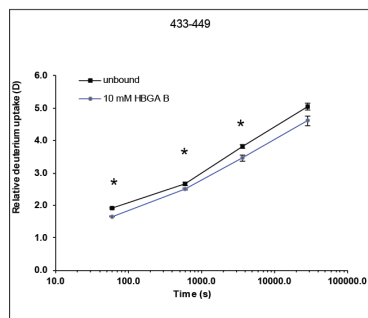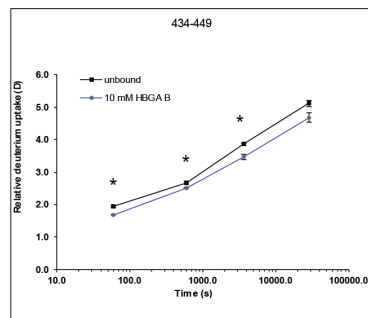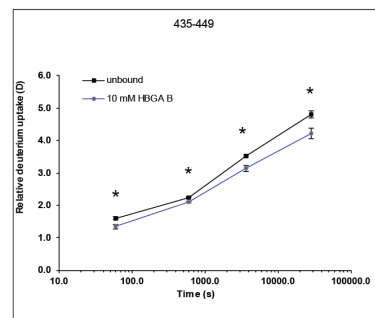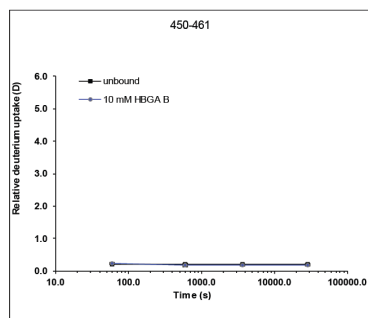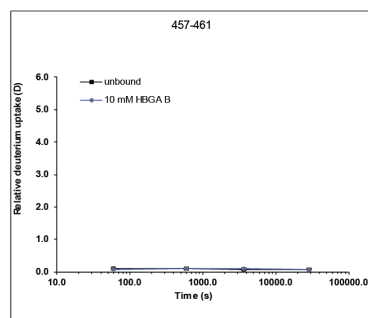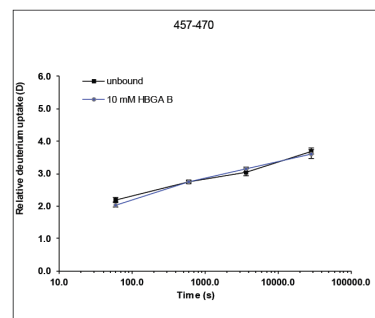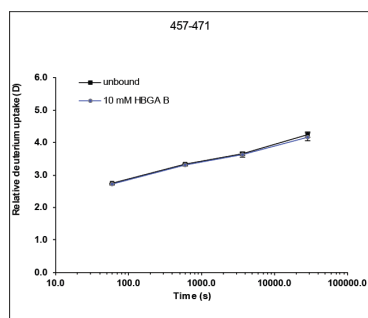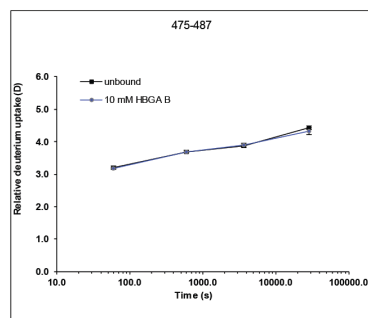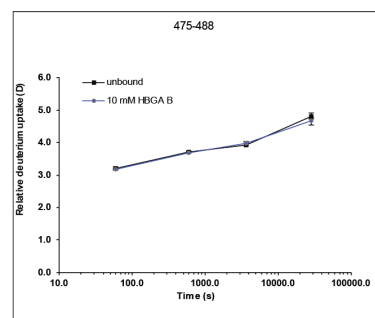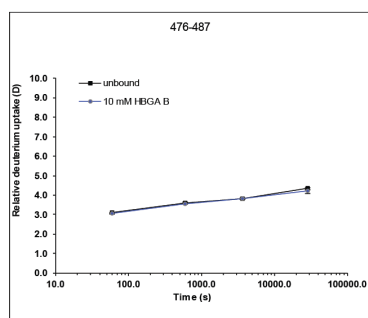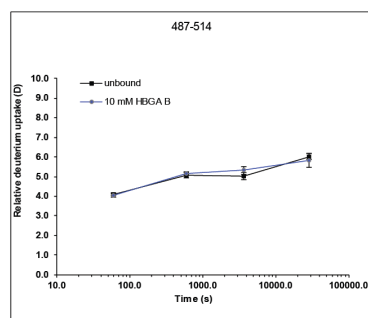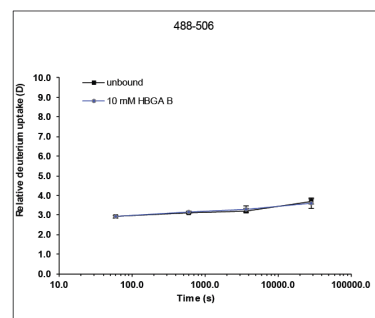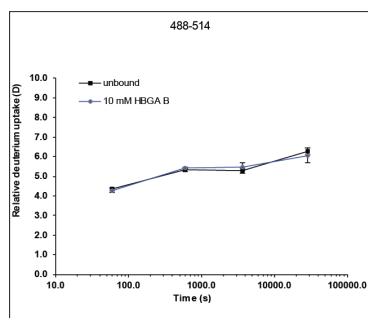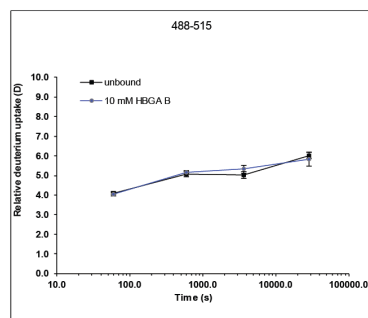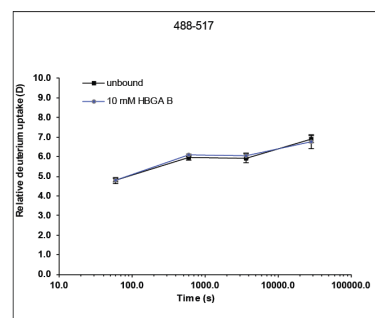

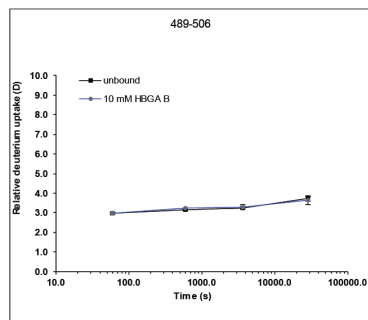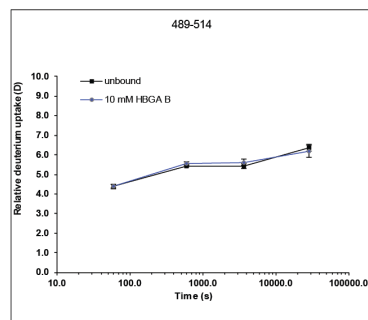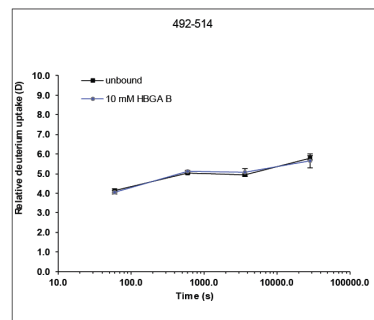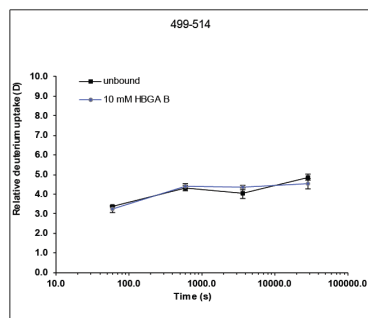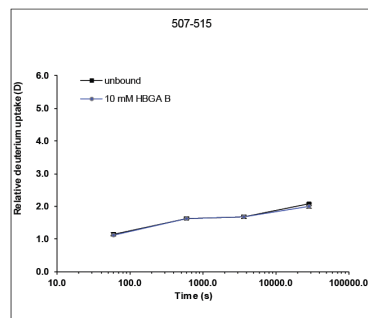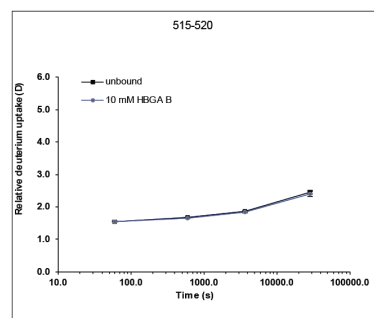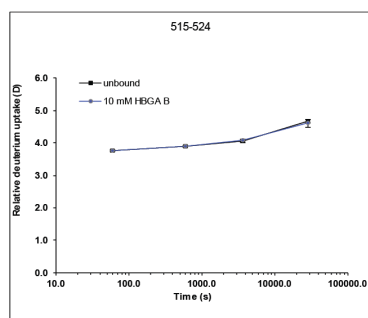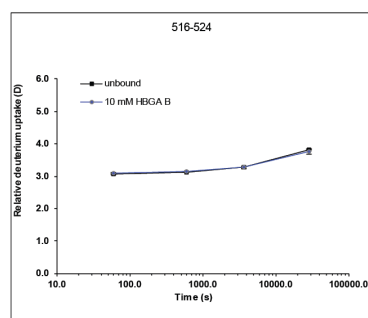

Supplement: Supplementary file 4 — Supplementary Data 1 [file 41467_2019_9251_MOESM4_ESM.pdf]

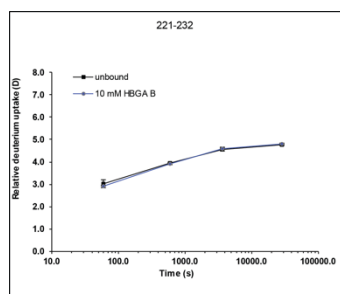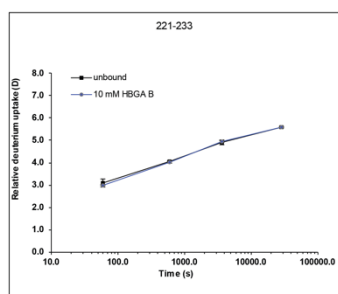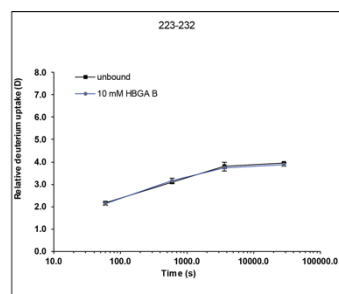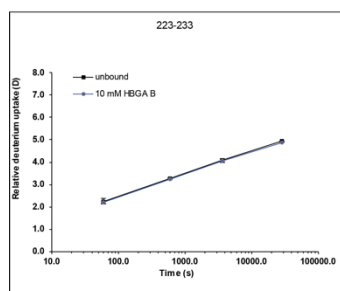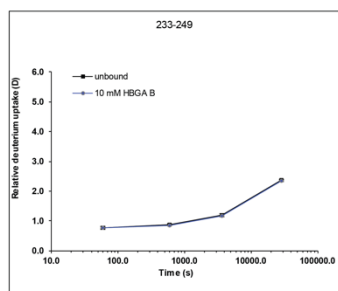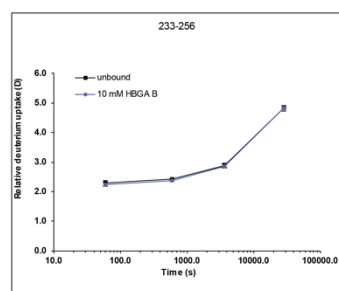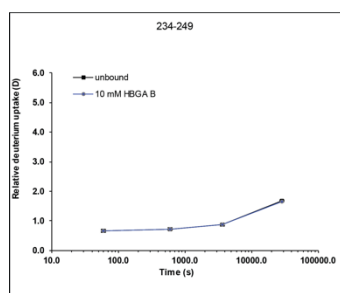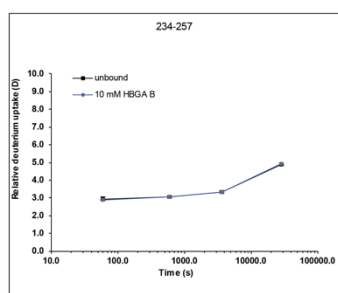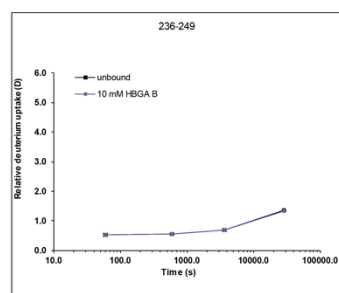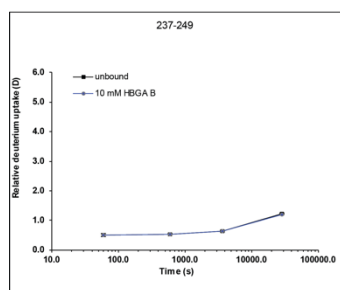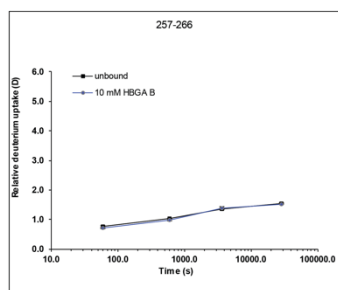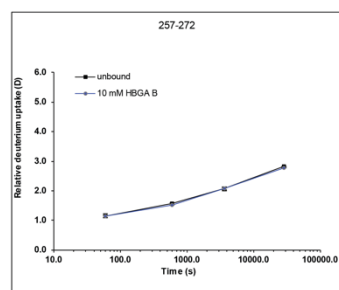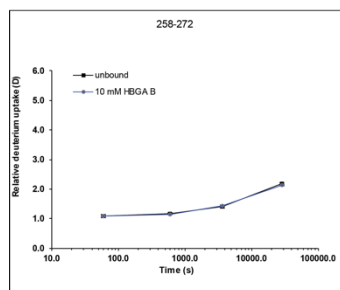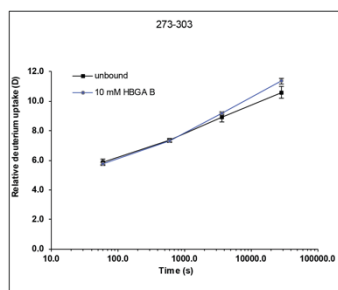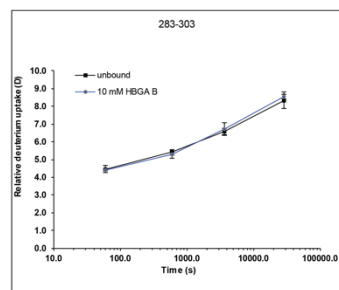

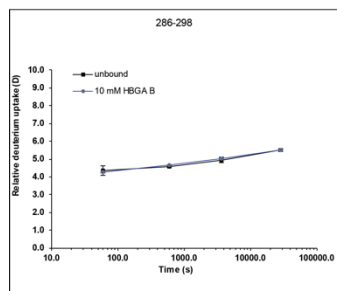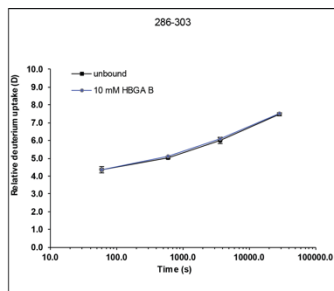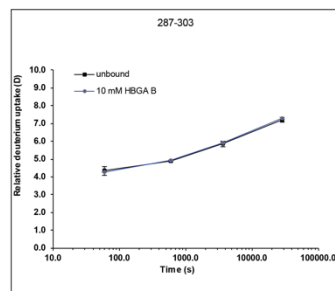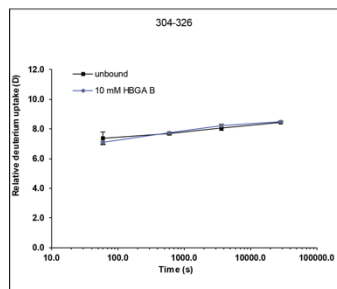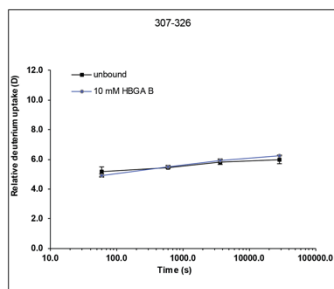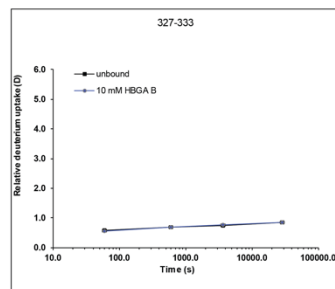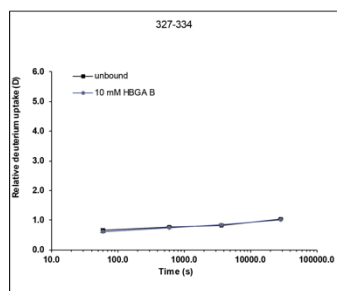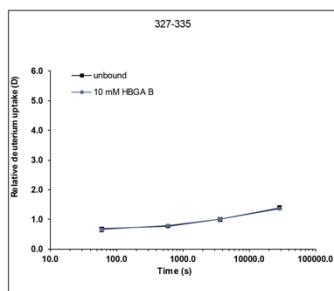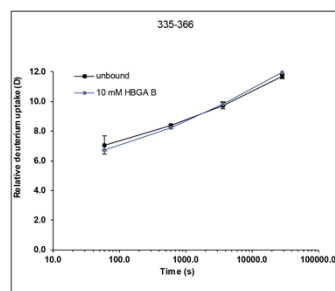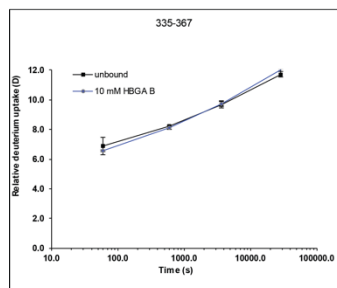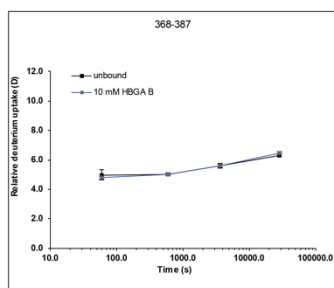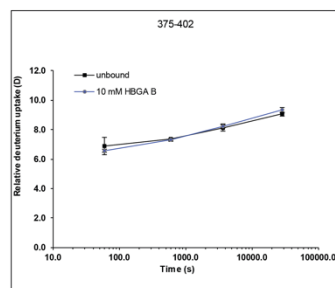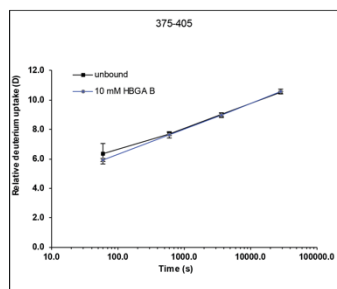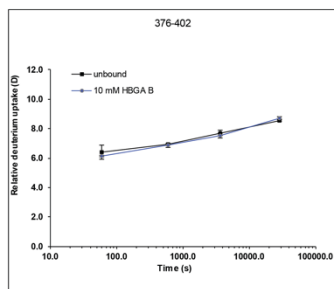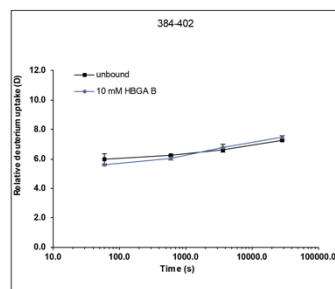

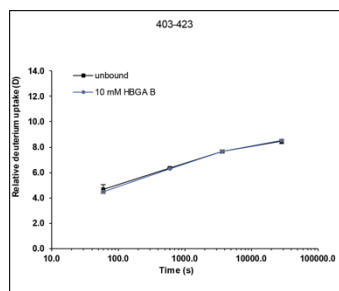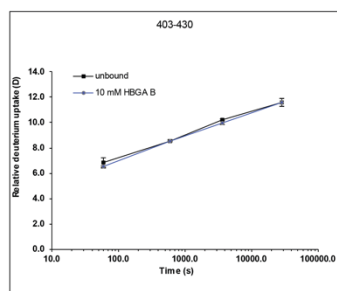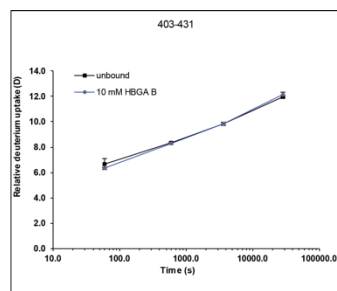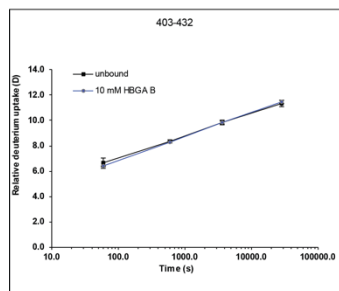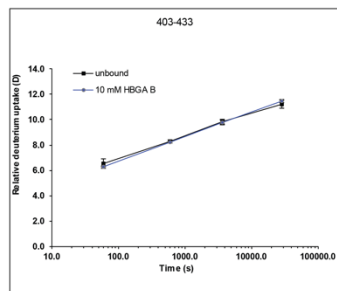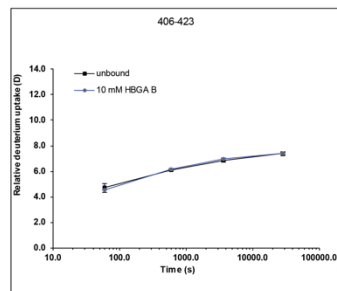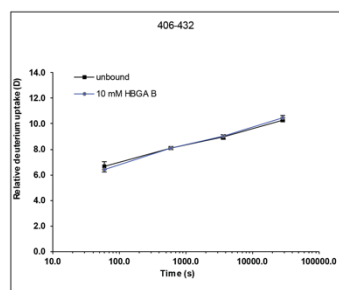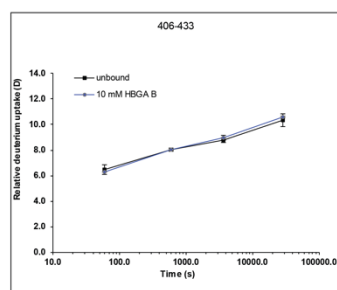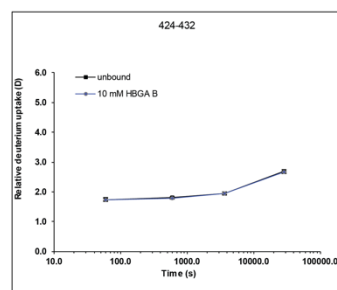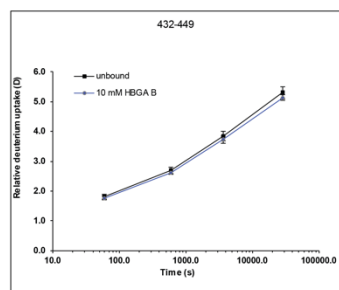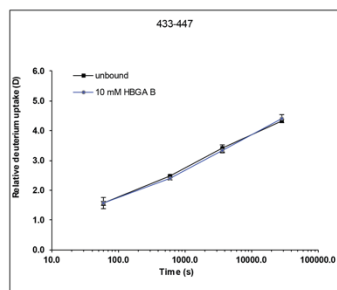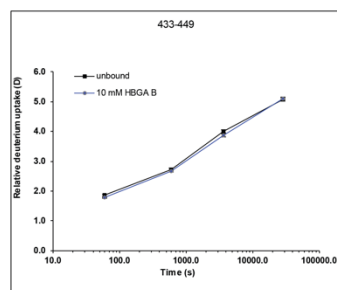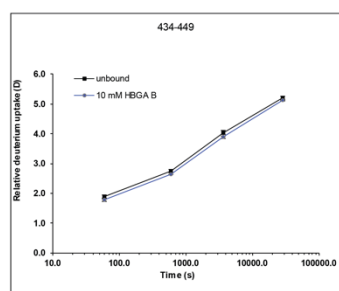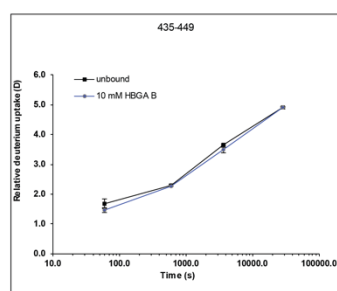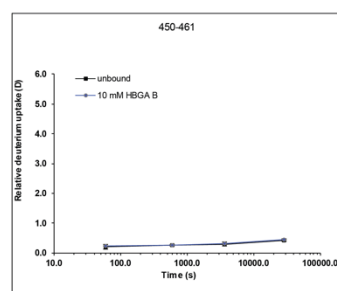

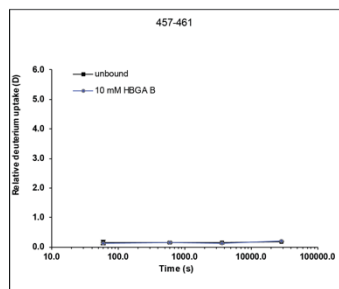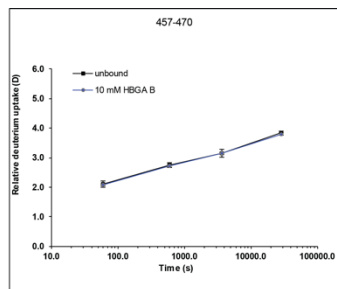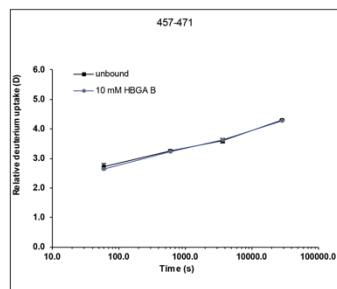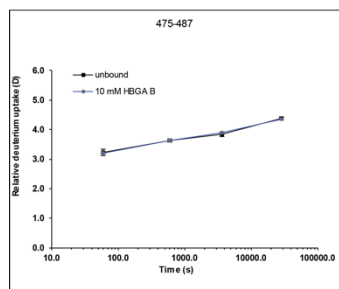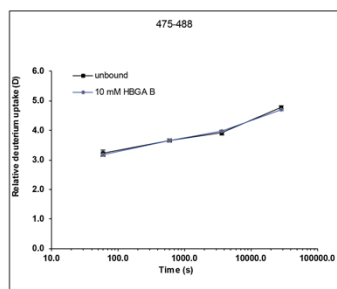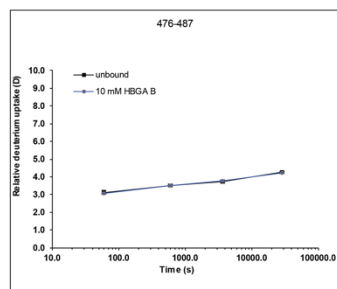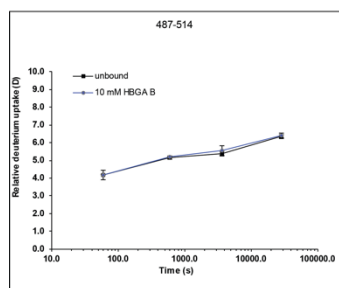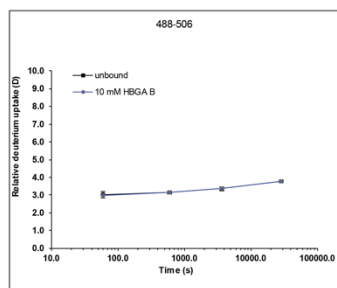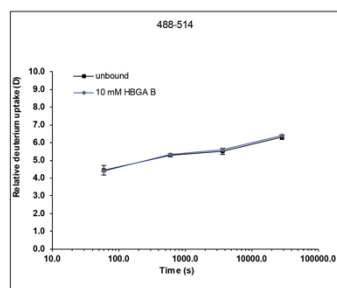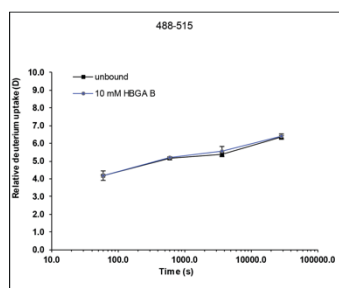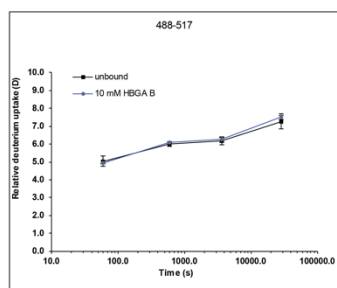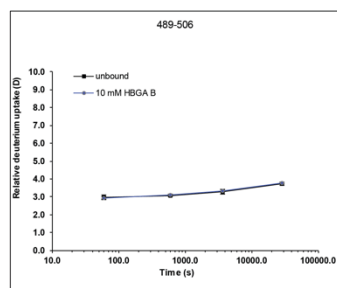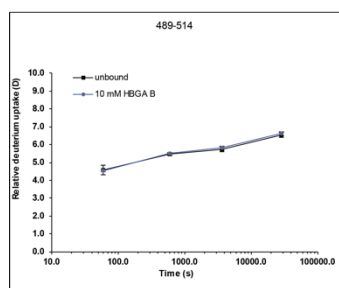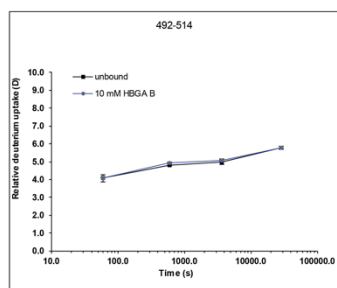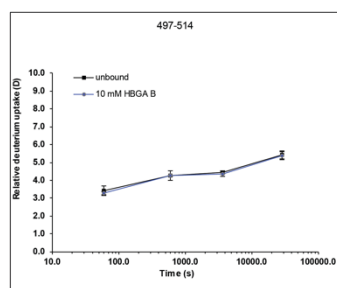

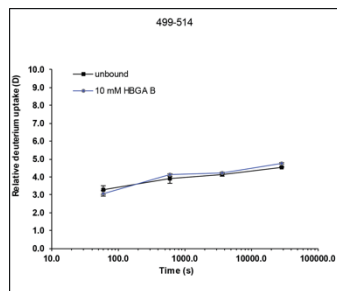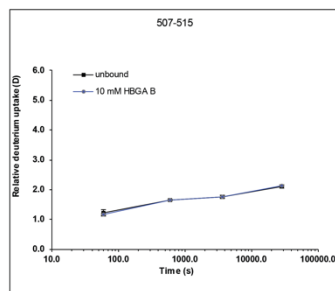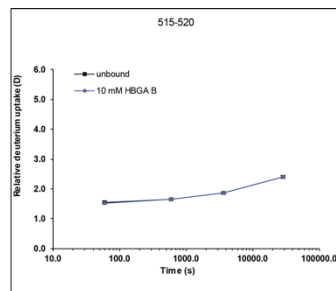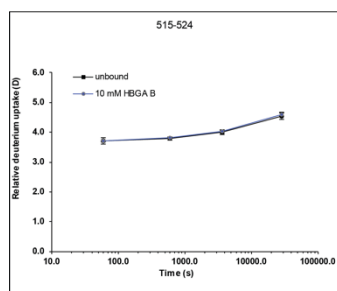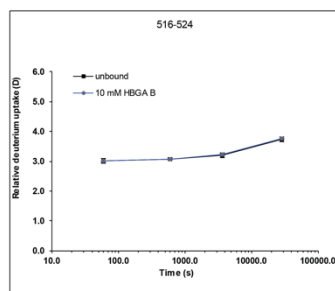

Supplement: Supplementary file 5 — Supplementary Data 2 [file 41467_2019_9251_MOESM5_ESM.pdf]

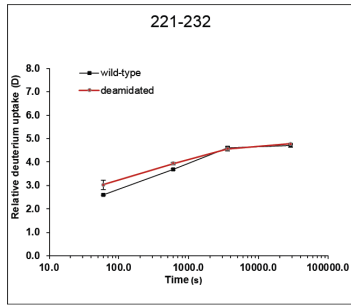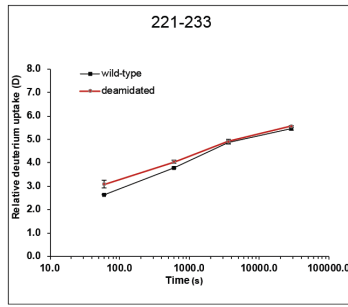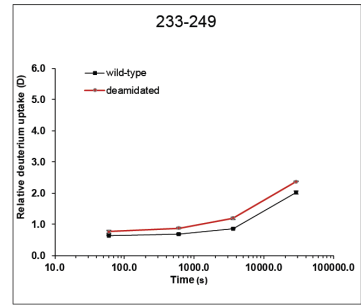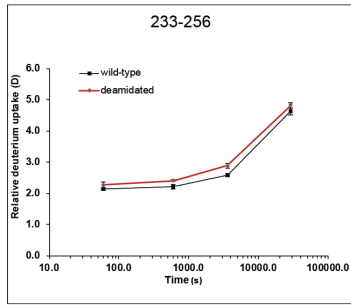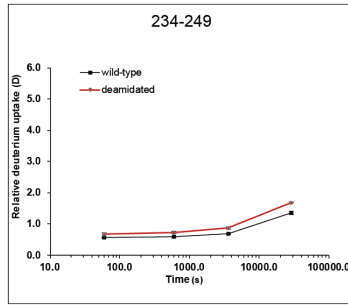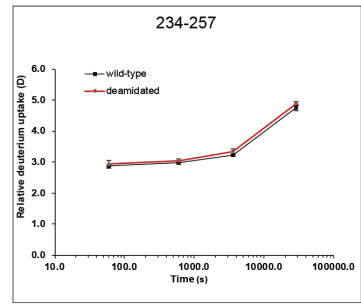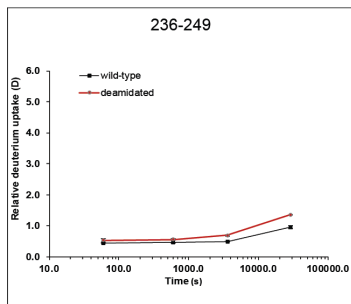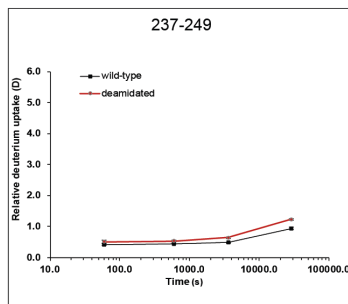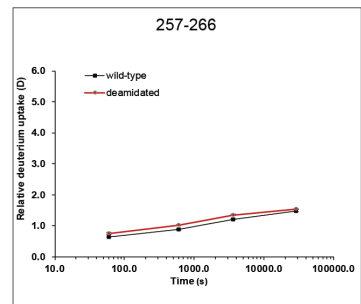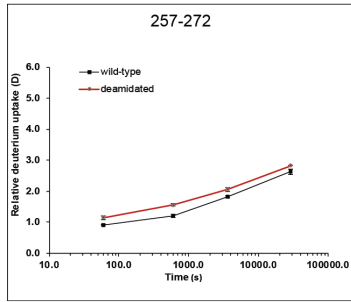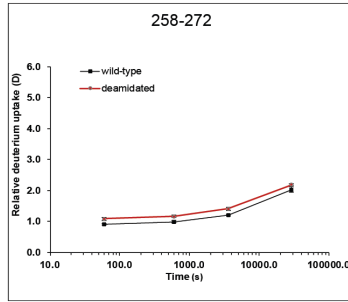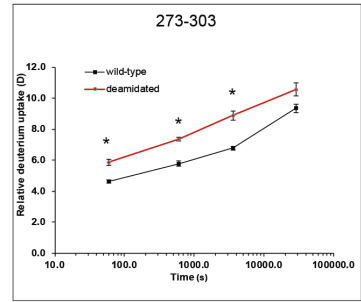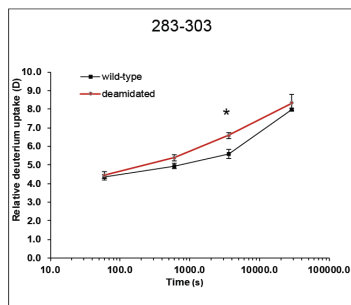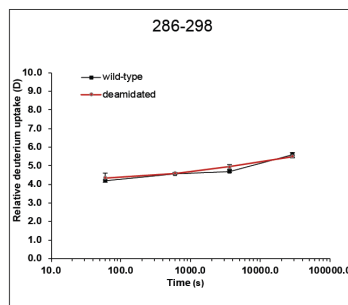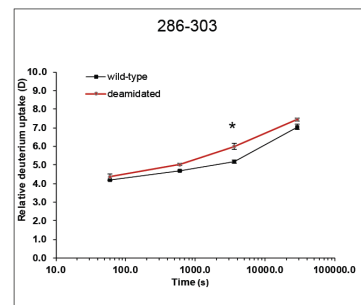

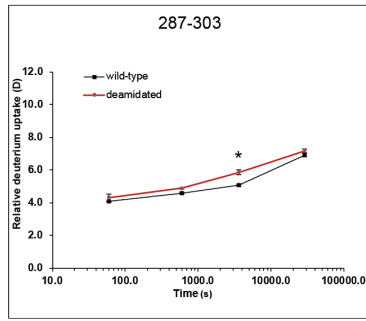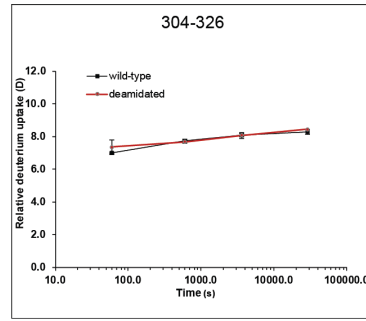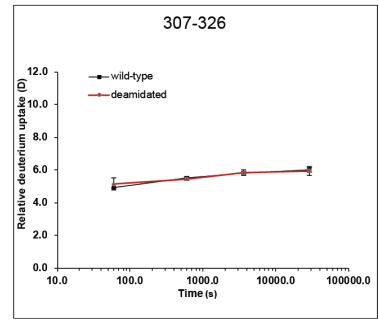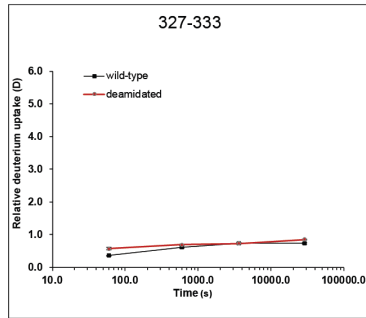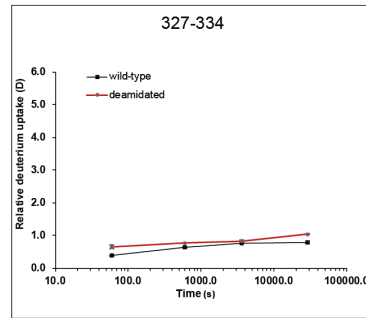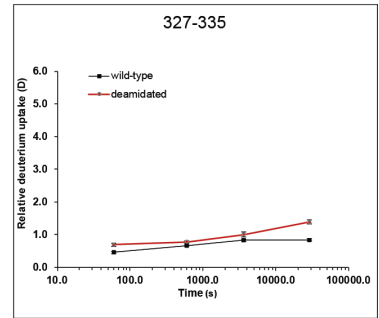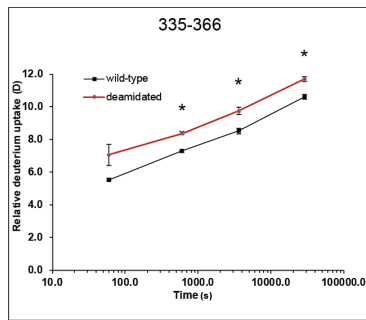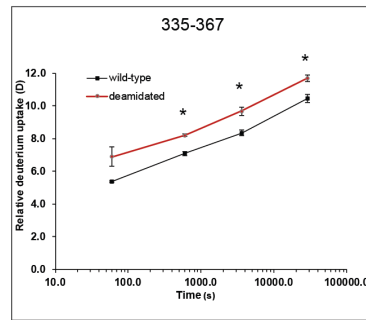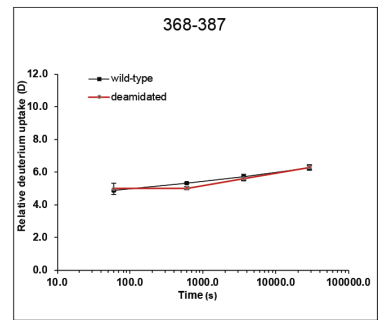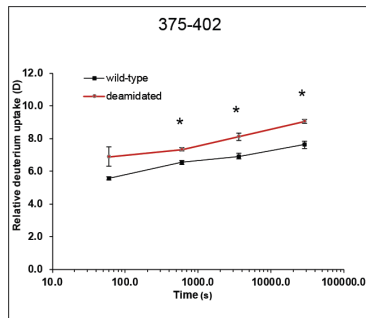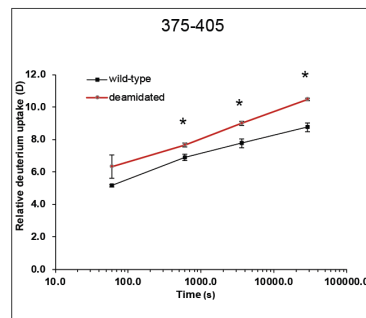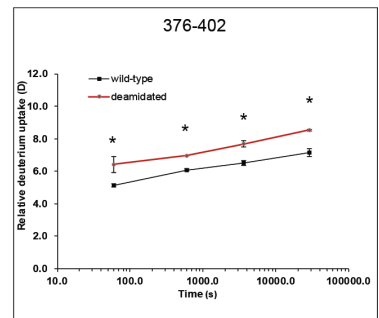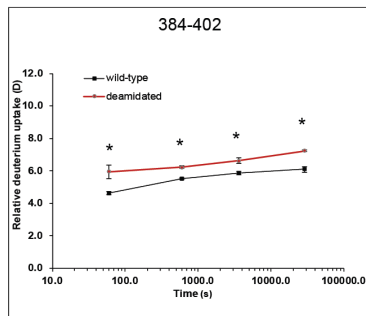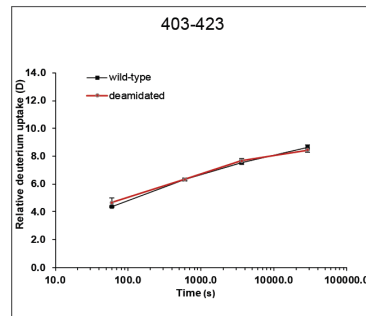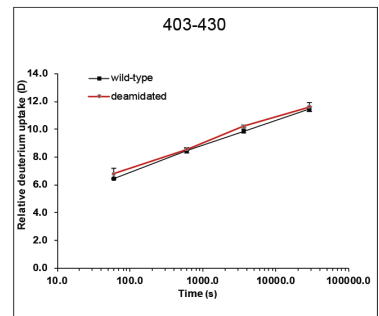

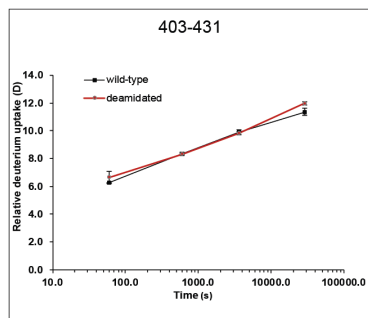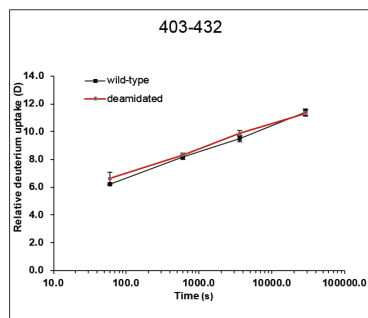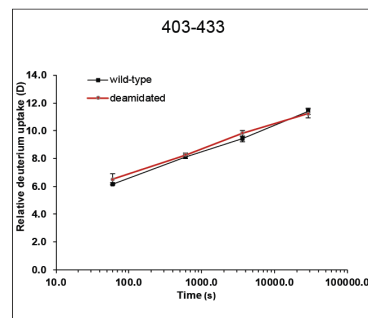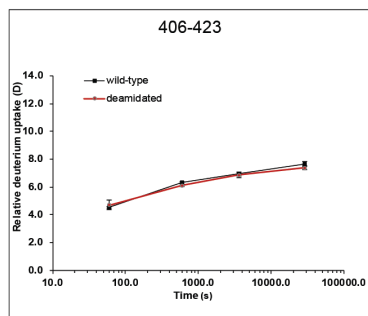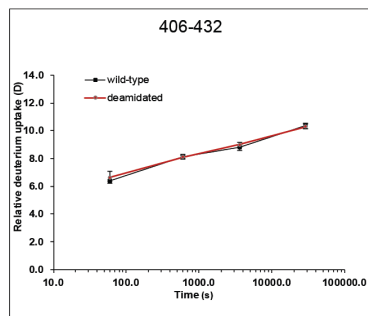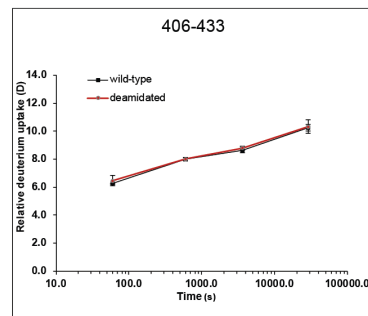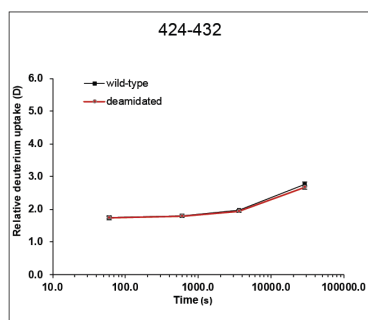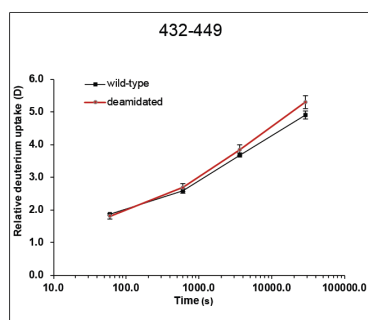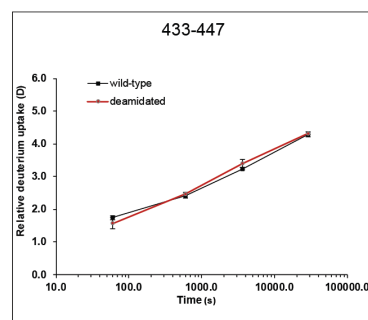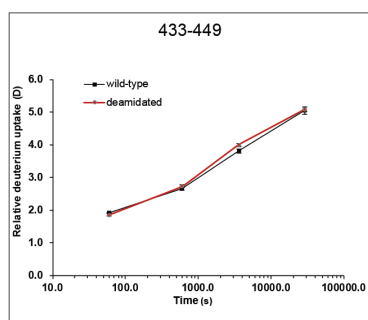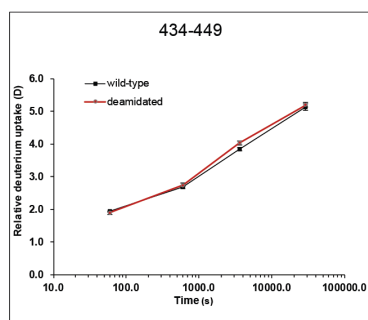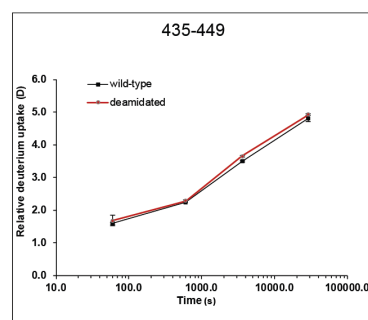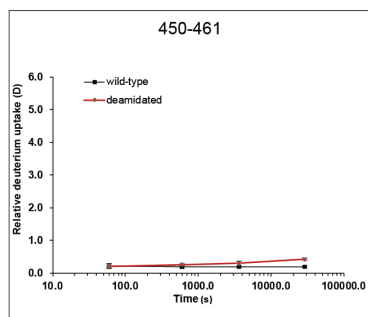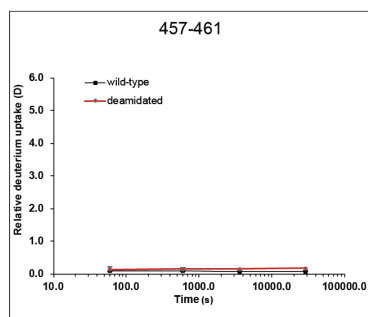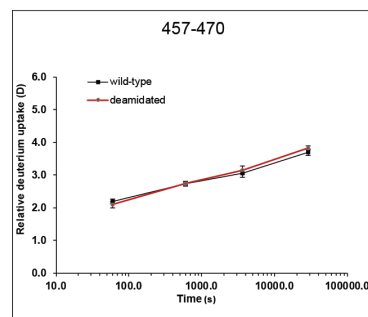

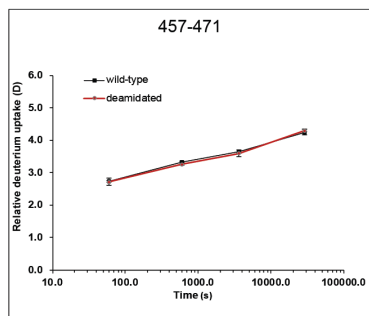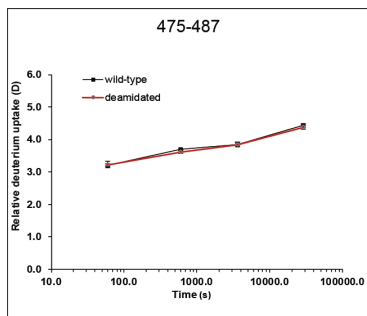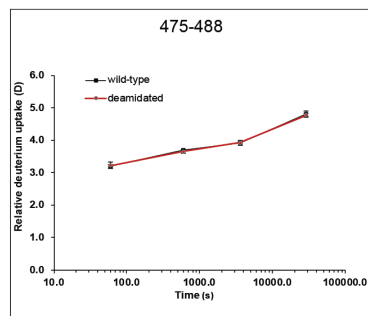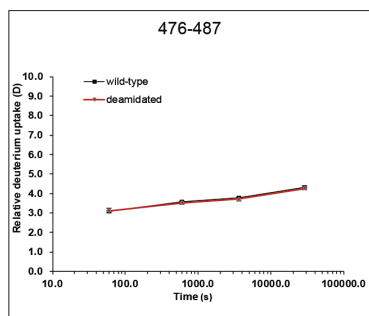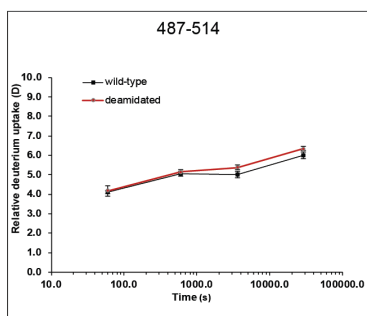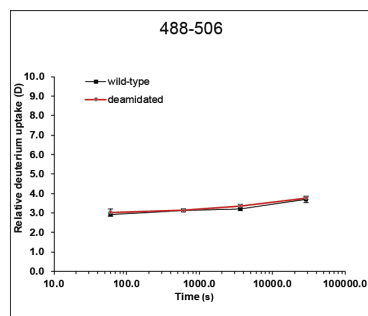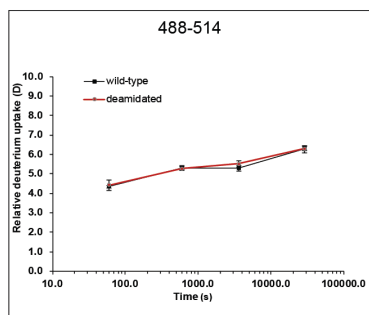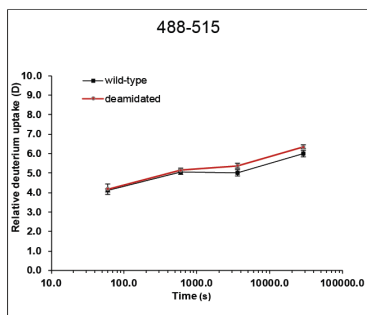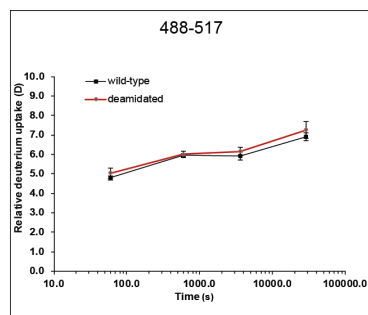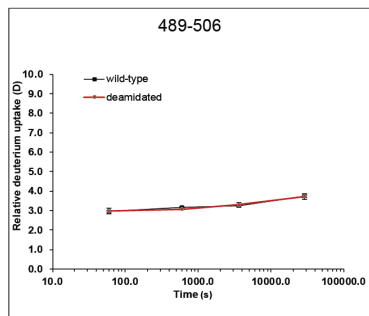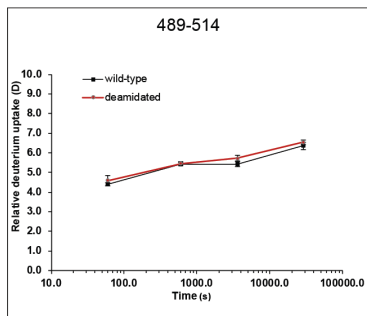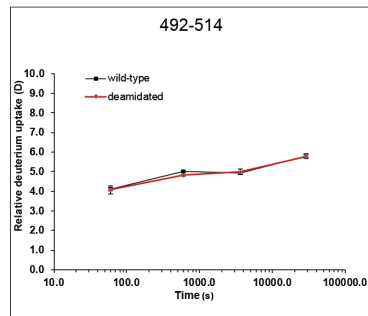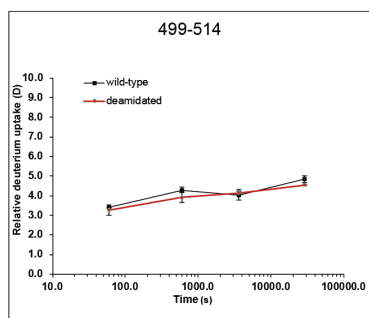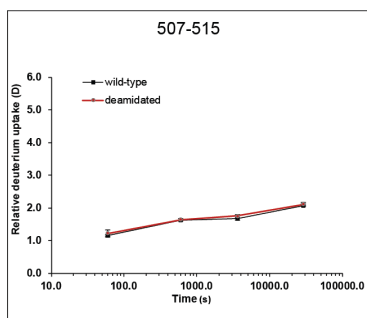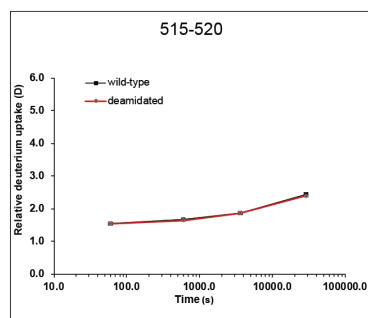

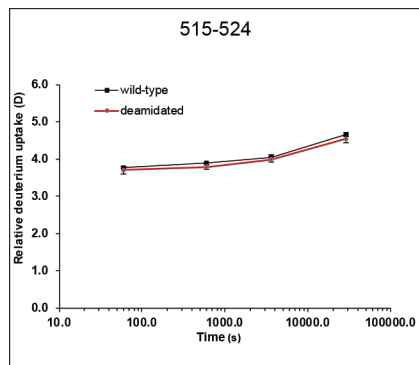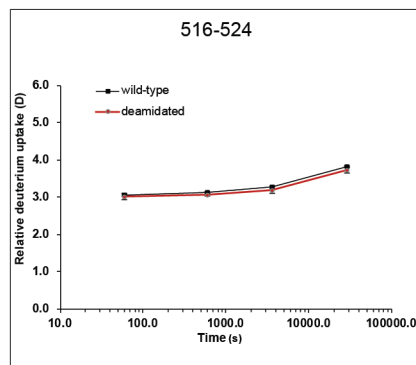

Supplement: Supplementary file 6 — Supplementary Data 3 [file 41467_2019_9251_MOESM6_ESM.pdf]

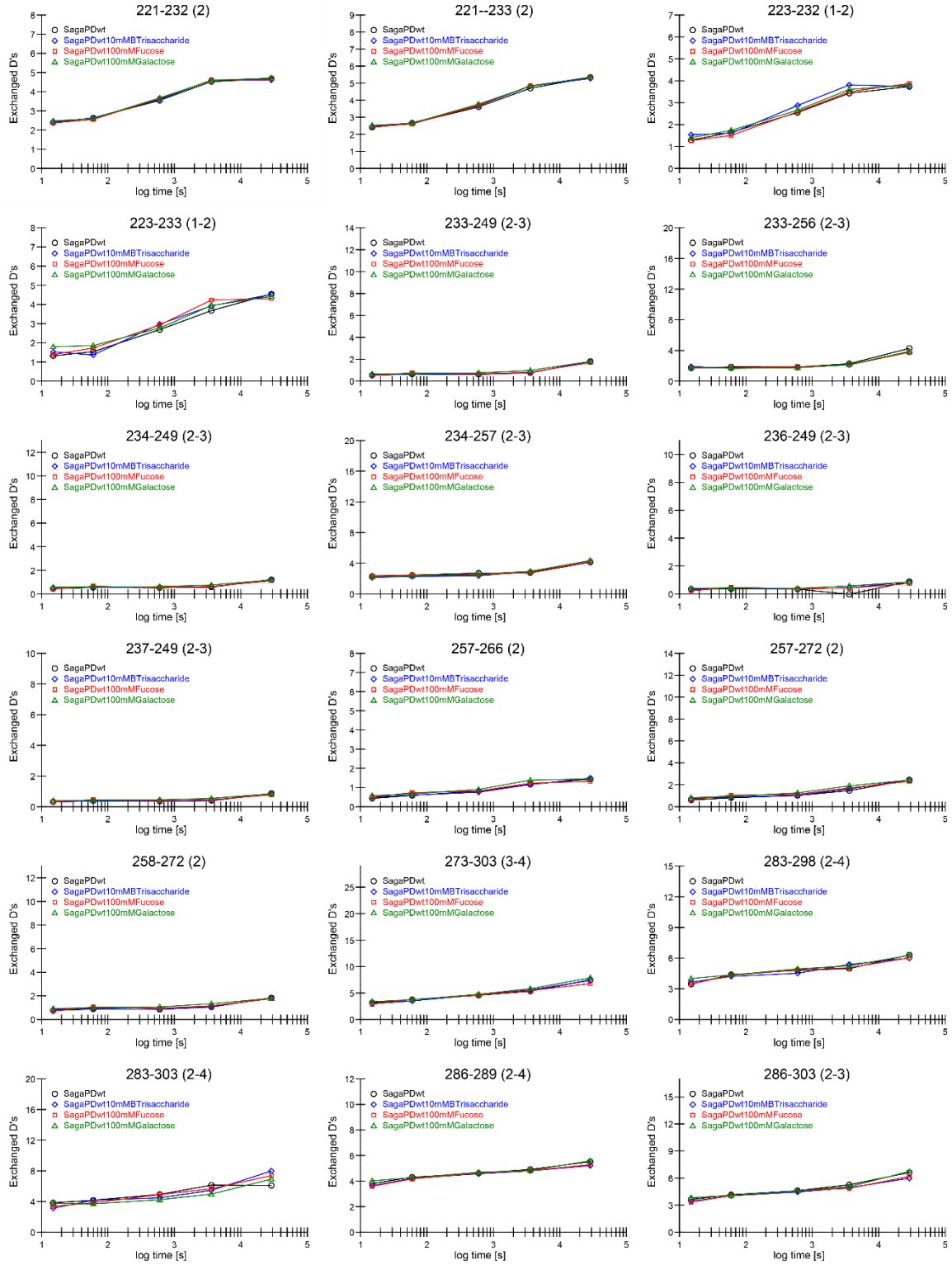

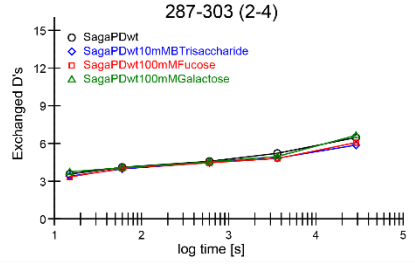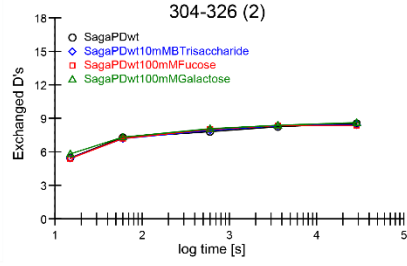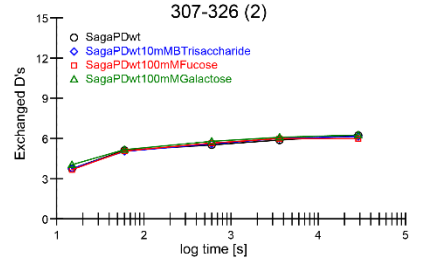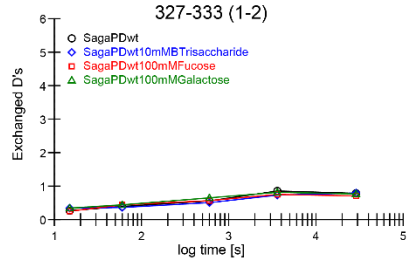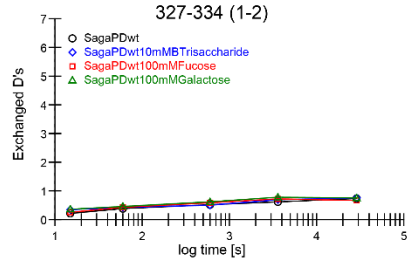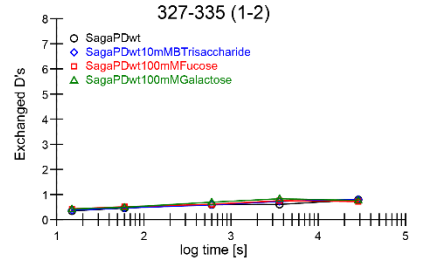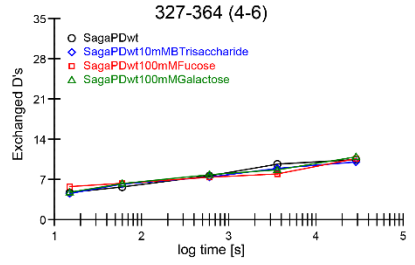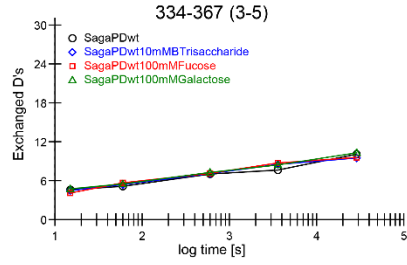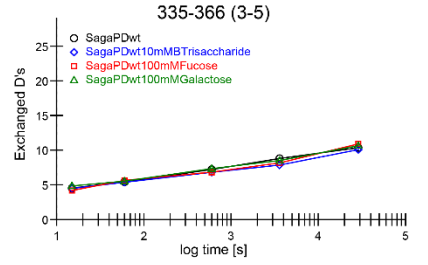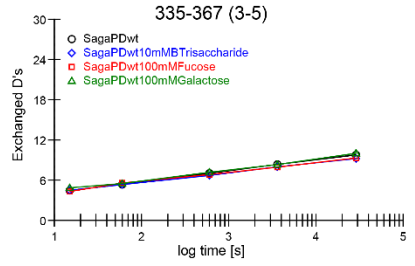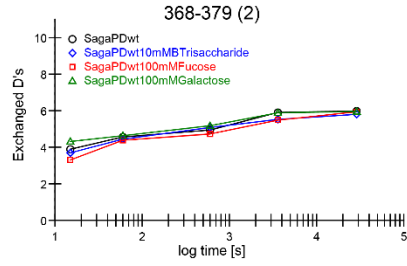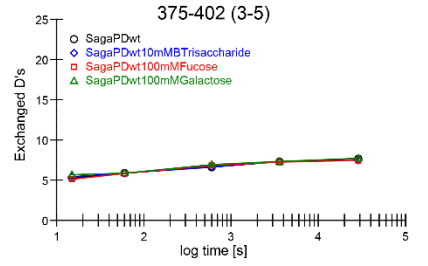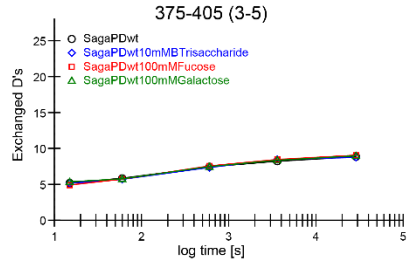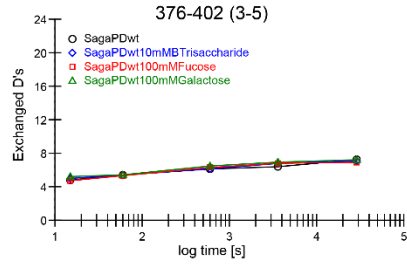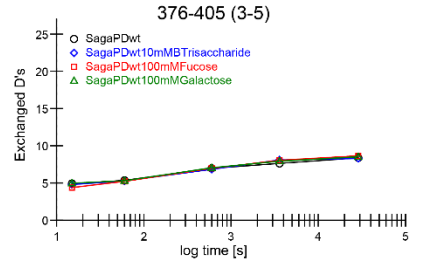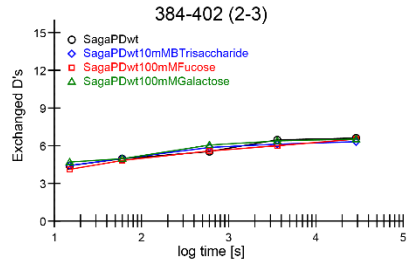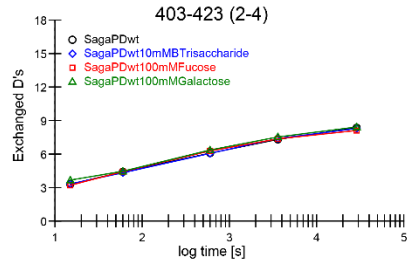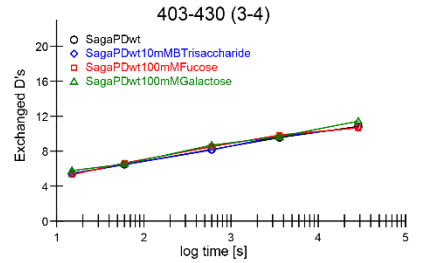

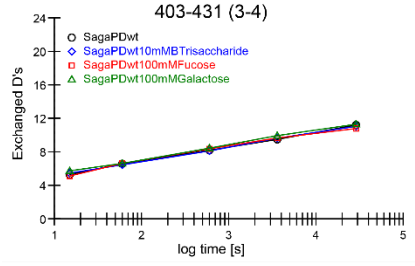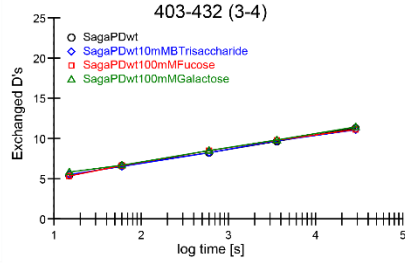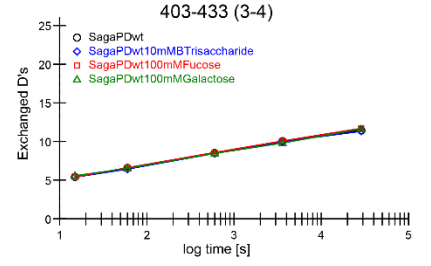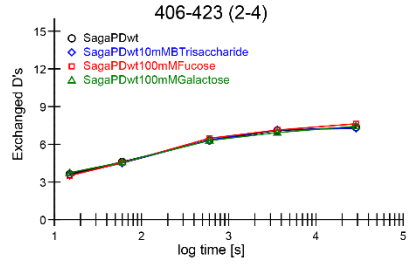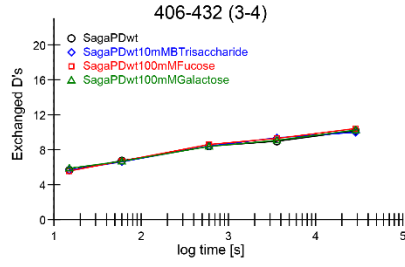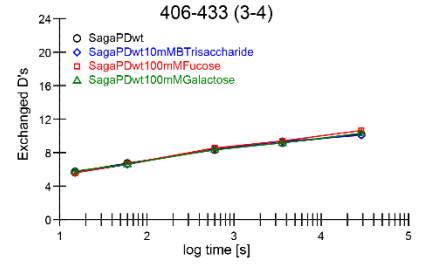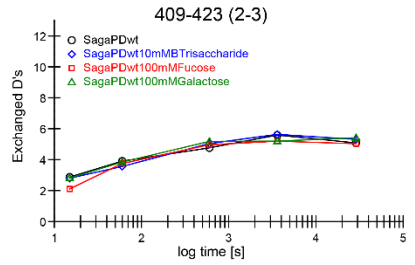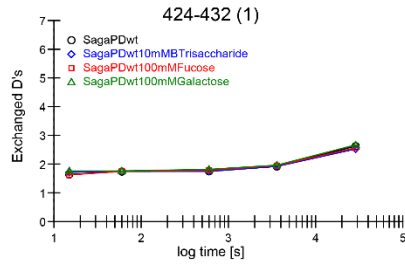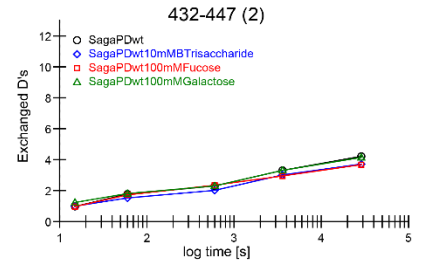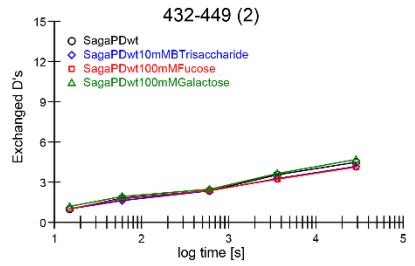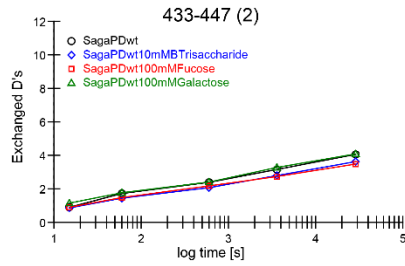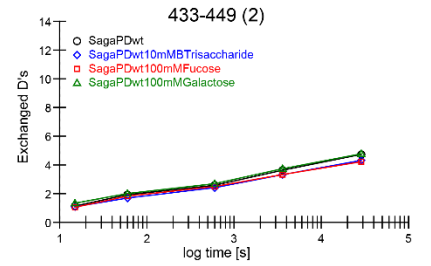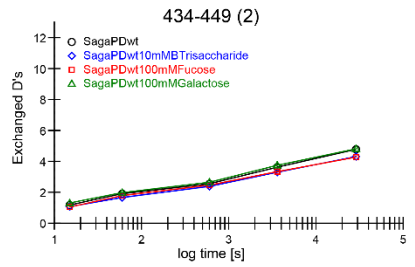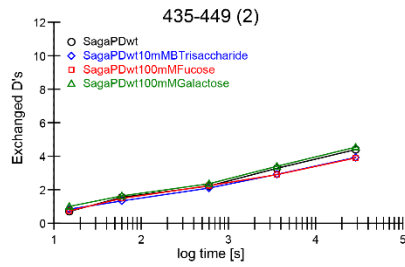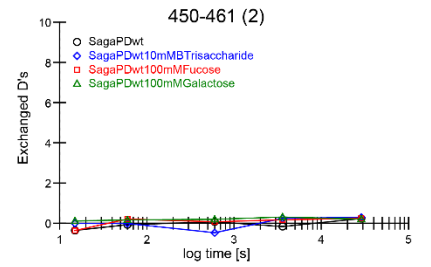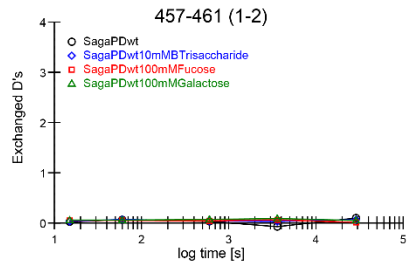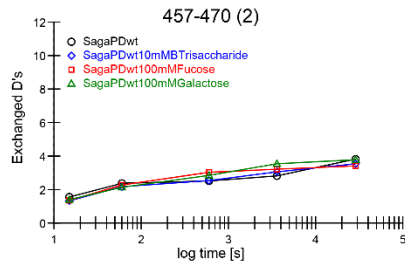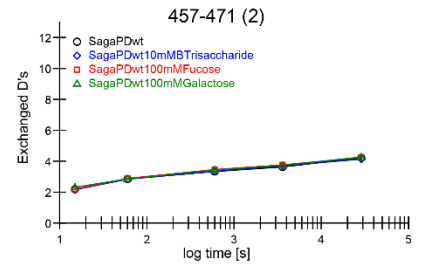

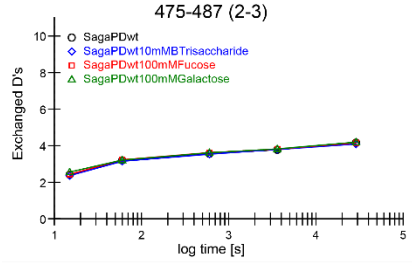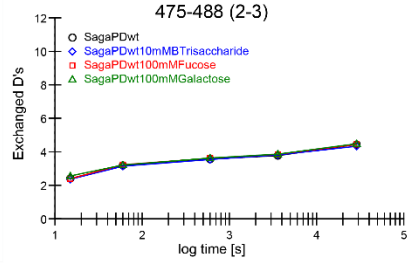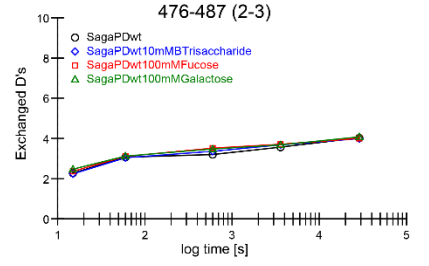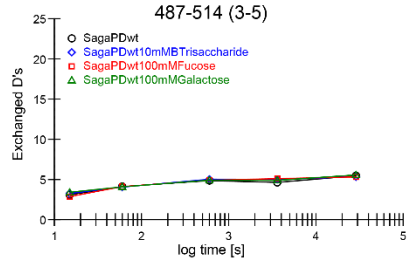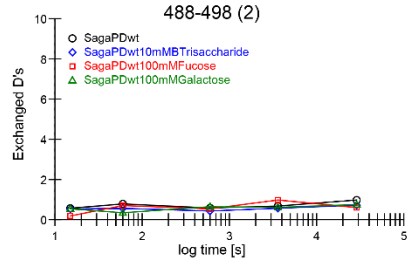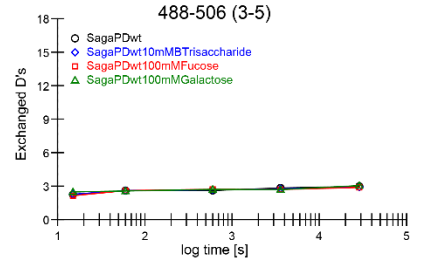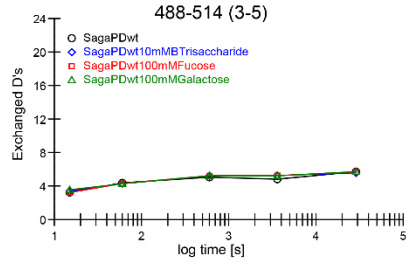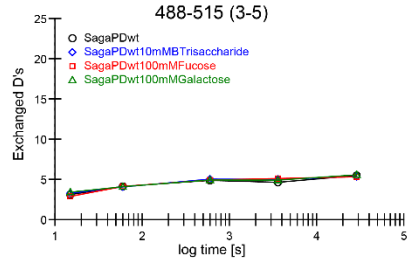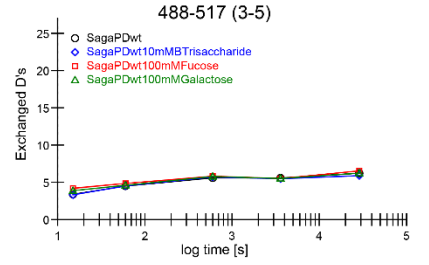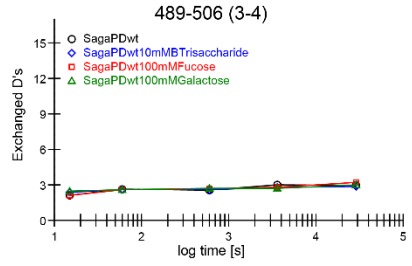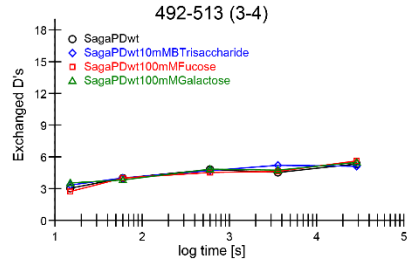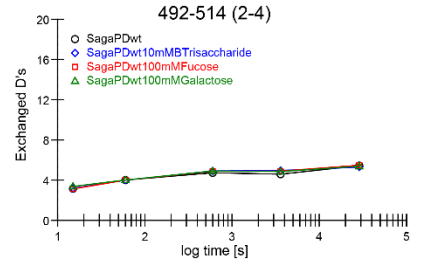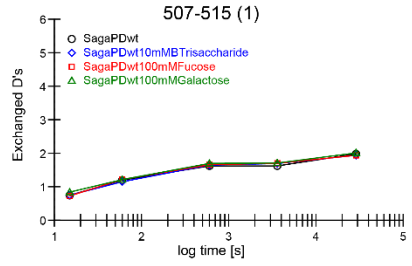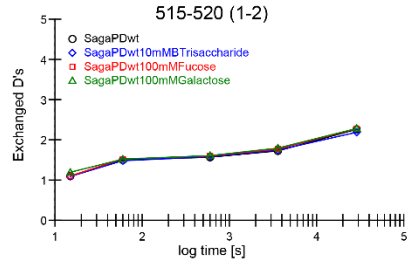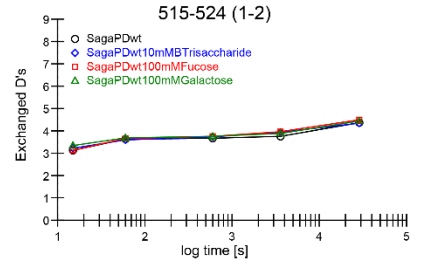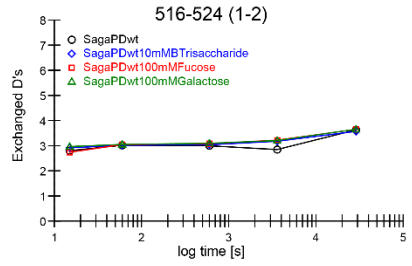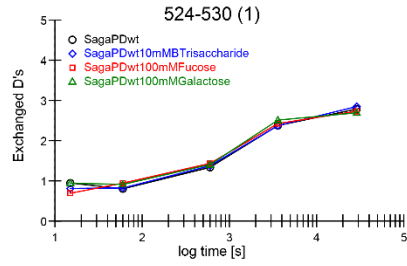

Supplement: Supplementary file 7 — Supplementary Data 4 [file 41467_2019_9251_MOESM7_ESM.pdf]
