# Supplementary material for: A post-translational modification of human Norovirus capsid protein attenuates glycan binding
Source: Nat Commun. 2019 Mar 21;10:1320. doi: 10.1038/s41467-019-09251-5 (PMC6428809; doi:10.1038/s41467-019-09251-5)
Supplement: Supplementary file 9 — Reporting Summary [file 41467_2019_9251_MOESM9_ESM.pdf]

nature research

Corresponding author(s): NCOMMS-18-28263

Reporting Summary

Nature Research wishes to improve the reproducibility of the work that we publish. This form provides structure for consistency and transparency in reporting. For further information on Nature Research policies, see Authors & Referees and the Editorial Policy Checklist.

Statistical parameters

When statistical analyses are reported, confirm that the following items are present in the relevant location (e.g. figure legend, table legend, main text, or Methods section).

n/a	Confirmed	
☐	☒	The exact sample size (*n*) for each experimental group/condition, given as a discrete number and unit of measurement
☐	☒	An indication of whether measurements were taken from distinct samples or whether the same sample was measured repeatedly
☐	☒	The statistical test(s) used AND whether they are one- or two-sided<br>*Only common tests should be described solely by name; describe more complex techniques in the Methods section.*
☒	☐	A description of all covariates tested
☒	☐	A description of any assumptions or corrections, such as tests of normality and adjustment for multiple comparisons
☐	☒	A full description of the statistics including central tendency (e.g. means) or other basic estimates (e.g. regression coefficient) AND variation (e.g. standard deviation) or associated estimates of uncertainty (e.g. confidence intervals)
☒	☐	For null hypothesis testing, the test statistic (e.g. *F*, *t*, *r*) with confidence intervals, effect sizes, degrees of freedom and *P* value noted<br>*Give P values as exact values whenever suitable.*
☒	☐	For Bayesian analysis, information on the choice of priors and Markov chain Monte Carlo settings
☒	☐	For hierarchical and complex designs, identification of the appropriate level for tests and full reporting of outcomes
☒	☐	Estimates of effect sizes (e.g. Cohen's *d*, Pearson's *r*), indicating how they were calculated
☐	☒	Clearly defined error bars<br>*State explicitly what error bars represent (e.g. SD, SE, CI)*

Our web collection on statistics for biologists may be useful.

Software and code

Policy information about availability of computer code

Data collection	NMR data acquisition: TopSpin 3.5 (Bruker)
Data analysis	NMR data analysis: TopSpin 3.5, CCPNMR 2.4.2, Maestro 11.8.012 (Schrödinger), Matlab 2016b, Matlab scripts; Origin 2016G, MS data analysis: MaxQuant, Xcalibur, DeutEx, GraphPad Prism, Excel

For manuscripts utilizing custom algorithms or software that are central to the research but not yet described in published literature, software must be made available to editors/reviewers upon request. We strongly encourage code deposition in a community repository (e.g. GitHub). See the Nature Research guidelines for submitting code & software for further information.

Data

Policy information about availability of data

All manuscripts must include a data availability statement. This statement should provide the following information, where applicable:

- Accession codes, unique identifiers, or web links for publicly available datasets
- A list of figures that have associated raw data
- A description of any restrictions on data availability

Crystal structure data will be available with the pdb accession code 6H9V; NMR chemical shifts will be available from BioMagRes bank with the accession code 27445. HDX data tables as well as MS raw data have been deposited to the ProteomeXchange Consortium via the PRIDE partner repository (dataset identifier

PXD011914). Figures 1, 2, and 5 contain raw data (NMR spectra). All figures except for Fig. 3 are based on experimental data. There are no restrictions on data availability. All data are available from the authors upon request.

Field-specific reporting

Please select the best fit for your research. If you are not sure, read the appropriate sections before making your selection.

☒ Life sciences ☐ Behavioural & social sciences ☐ Ecological, evolutionary & environmental sciences

For a reference copy of the document with all sections, see nature.com/authors/policies/ReportingSummary-flat.pdf

Life sciences study design

All studies must disclose on these points even when the disclosure is negative.

Sample size	Isotope labeled protein samples were subjected to multidimensional NMR spectroscopy. The assignment process does not require repeated data acquisition. For chemical shift perturbation experiments it is common to use one data set. HDX MS experiments have been performed in triplicate.
Data exclusions	No data were excluded.
Replication	HSQC NMR experiments of different protein batches yield the same spectra. MS HDX experiments have been performed in triplicate.
Randomization	Randomization does not apply to these studies
Blinding	Blinding also does not apply to these studies

Reporting for specific materials, systems and methods

Materials & experimental systems

n/a	Involved in the study	
☒	☐	Unique biological materials
☒	☐	Antibodies
☒	☐	Eukaryotic cell lines
☒	☐	Palaeontology
☒	☐	Animals and other organisms
☒	☐	Human research participants

Methods

n/a	Involved in the study	
☒	☐	ChIP-seq
☒	☐	Flow cytometry
☒	☐	MRI-based neuroimaging
